# Genome-wide association studies and genomic selection assays made in a large sample of cacao (*Theobroma cacao* L.) germplasm reveal significant marker-trait associations and good predictive value for improving yield potential

**DOI:** 10.1371/journal.pone.0260907

**Published:** 2022-10-06

**Authors:** Frances L. Bekele, Gillian G. Bidaisee, Mathilde Allegre, Xavier Argout, Olivier Fouet, Michel Boccara, Duraisamy Saravanakumar, Isaac Bekele, Claire Lanaud

**Affiliations:** 1 Cocoa Research Centre, The University of The West Indies, St. Augustine, Trinidad and Tobago; 2 Centre de Coopération Internationale en Recherche Agronomique pour le Développement (CIRAD), UMR AGAP Institute, Montpellier, France; 3 AGAP Institute, CIRAD, INRAE, Institut Agro, University of Montpellier, Montpellier, France; 4 Department of Food Production, Faculty of Food and Agriculture, The University of the West Indies, St Augustine, Trinidad and Tobago; 5 Faculty of Food and Agriculture, The University of the West Indies, St Augustine, Trinidad and Tobago; CSIR-Institute of Himalayan Bioresource Technology, INDIA

## Abstract

A genome-wide association study (GWAS) was undertaken to unravel marker-trait associations (MTAs) between SNP markers and phenotypic traits. It involved a subset of 421 cacao accessions from the large and diverse collection conserved *ex situ* at the International Cocoa Genebank Trinidad. A Mixed Linear Model (MLM) in *TASSEL* was used for the GWAS and followed by confirmatory analyses using GAPIT FarmCPU. An average linkage disequilibrium (*r*^*2*^) of 0.10 at 5.2 Mb was found across several chromosomes. Seventeen significant (*P* ≤ 8.17 × 10^−5^ (–log10 (p) = 4.088)) MTAs of interest, including six that pertained to yield-related traits, were identified using *TASSEL* MLM. The latter accounted for 5 to 17% of the phenotypic variation expressed. The highly significant association (*P* ≤ 8.17 × 10^−5^) between seed length to width ratio and TcSNP 733 on chromosome 5 was verified with FarmCPU (*P ≤* 1.12 × 10^−8^). Fourteen MTAs were common to both the *TASSEL* and FarmCPU models at *P* ≤ 0.003. The most significant yield-related MTAs involved seed number and seed length on chromosome 7 (*P* ≤ 1.15 × 10−^14^ and *P* ≤ 6.75 × 10−^05^, respectively) and seed number on chromosome 1 (*P* ≤ 2.38 × 10^−05^), based on the *TASSEL* MLM. It was noteworthy that seed length, seed length to width ratio and seed number were associated with markers at different loci, indicating their polygenic nature. Approximately 40 candidate genes that encode embryo and seed development, protein synthesis, carbohydrate transport and lipid biosynthesis and transport were identified in the flanking regions of the significantly associated SNPs and in linkage disequilibrium with them. A significant association of fruit surface anthocyanin intensity co-localised with MYB-related protein 308 on chromosome 4. Testing of a genomic selection approach revealed good predictive value (genomic estimated breeding values (GEBV)) for economic traits such as seed number (GEBV = 0.611), seed length (0.6199), seed width (0.5435), seed length to width ratio (0.5503), seed/cotyledon mass (0.6014) and ovule number (0.6325). The findings of this study could facilitate genomic selection and marker-assisted breeding of cacao thereby expediting improvement in the yield potential of cacao planting material.

## Introduction

Cacao, *Theobroma cacao* L., Malvaceae *sensu lato* [1], is an important Neotropical, perennial crop, on which the thriving global cocoa and chocolate industry is based. The World Cocoa Foundation, in 2012, reported that 40–50 million people worldwide depend on cocoa for their livelihood. The global export value of cocoa beans has been fluctuating between 8 and 10.5 billion USD over the past decade and the global chocolate market was valued at USD 106.6 billion in 2019 [2]. *T*. *cacao* is a diploid (2n = 20), allogamous species. Its genome is small, reported by Argout et al. [3] to span 411–494 Mb. Its putative centre of genetic diversity is at the headwaters of the Amazon River, South America [4], and it is indigenous to the Amazon and Orinoco rainforests.

In the current context of climate change and disease evolution, it is important to acquire a comprehensive understanding of the genetic architecture of traits of interest such as disease and pest resistance, high yield, desirable flavour, favourable flavanol (antioxidant) content, self-compatibility, and other traits of economic relevance or with potential health benefits [5–8]. Of these, yield and disease resistance traits are mainly polygenic [9, 10] and tagging such traits with molecular markers will facilitate early selection for the desired genotypes and formulation of breeding strategies to create new, adapted varieties. The considerable progress made in cacao genomics in the last decade [7, 11–34], along with the increase in the number of available markers, will help to accelerate progress in improvement of cacao planting material.

Genome-wide association studies (GWAS) have been used widely in breeding to detect significant population-wide associations between genetic markers, such as Single Nucleotide Polymorphisms (SNPs), and causal polymorphisms, with functional roles in controlling traits of interest like yield potential [35, 36]. The search for genotype-phenotype correlations is conducted in unrelated individuals [37, 38], and is based on non-random association of alleles in the population and linkage disequilibrium (LD) [39, 40]. Robust GWAS models, using population structure and kinship matrices as co-variates, facilitate the identification of mainly non-spurious associations [36].

This application of GWAS in *T*. *cacao* L. is facilitated by the mapping of the cacao genome and several previous studies. Allegre et al. [33] and Fouet et al. [32] identified and mapped SNPs and microsatellite (SSR) markers useful as expressed sequence tags (ESTs) and constructed a high-density genetic map for *T*. *cacao*. Both kinds of markers are co-dominant and thus powerful for genetic analysis. Allegre et al. [33], mapped 851 SNP markers corresponding to genes with putative functions and displaying a distinct polymorphic pattern across a selection of cacao germplasm. These SNPs are located within a gene expressed sequence and thus valuable for identifying candidate genes with functional roles in cacao. The data are available at http://tropgenedb.cirad.fr.

The sequencing of the cacao genome [3, 29] should facilitate the identification of candidate genes for traits of interest, which are co-localized with marker-trait associations (MTAs). Version 2.0 of the Criollo genome of Argout et al. [29] has 99% of the assembly anchored to the 10 chromosomes of the *T*. *cacao L*. genome. This resource was utilized in this study.

The objectives of this research were to exploit the naturally occurring genetic variation in a large collection of cacao trees of diverse origin, conserved *ex situ*, to perform GWAS to identify SNP markers significantly associated with phenotypic traits, examine linkage disequilibrium patterns on the chromosomes, and locate putative annotated candidate genes that encode yield-related and other traits. A genomic selection approach was then used to establish predictive values for phenotypic traits of interest in order to examine the efficiency of this breeding strategy to improve cacao yield.

## Materials and methods

### Plant material

The germplasm studied consisted of four hundred and twenty-one (421) cacao accessions, including 263 wild genotypes (collected in the Amazon Basin [41]), which are conserved at the International Cocoa Genebank Trinidad (ICGT). Complete phenotypic data were collated for 346 of these accessions and incomplete data were accrued for 75 accessions (S1 Table http://dx.doi.org/10.13140/RG.2.2.16179.71202 and S2 Table http://dx.doi.org/10.13140/RG.2.2.29601.48484). The accessions represent 23 “accession groups”, as described by Bekele et al. [41], as well as most of the genetic groups defined by Motamayor et al. [42], Wild cacao types such as those of the AMAZ, GU, IMC, MO, NA, PA, POUND, RB, SCA and SPEC (1–54) accession groups [41], which have evolved over a long period of time, were included to improve the power of detection of associations between SNP markers and phenotypic traits of interest as recommended by Stack et al. [40].

Plant material was collected from the 421 accessions at the ICGT. Sample collection spanned the period 1992–2012. The ICGT is situated at the University Cocoa Research Station, Centeno, North-East Trinidad at an altitude of 15 m above sea level. Shade is provided by trees of *Erythrina* sp. and bananas (*Musa* sp.) planted 6 m and 4 m apart, respectively. Each plot typically had 16 trees of a specific accession, which are planted 1.8 m apart. The soil type is Cunupia fine sandy clay with restricted internal drainage.

Over a 30-year period from 1981, the average temperature in this region was 26.3ºC. It was 26ºC for the period 1961 to 1991. This satisfied the optimal temperature requirements for growing cacao. The mean annual rainfall for 1981 to 2011 was 1945.2 mm, lower than the 2,392 mm recorded for 1961 to 1991 (Trinidad and Tobago Meteorological Office https://www.metoffice.gov.tt). The trees received irrigation as necessary during the dry season (January-June) each year. The plots were maintained with standard production practices regarding weeding and pruning. However, no pesticides or fungicides were applied to allow monitoring of the natural reactions of the trees to pest and diseases and yield parameters. Fertilizers were applied at planting and regularly for only young trees and the trees were maintained within a low input system.

### Phenotypic evaluation

The cacao accessions were assessed in terms of 27 flower, fruit and seed traits (Table 1) as described by Bekele et al. and Bekele and Butler [41, 43]. The traits studied were found to be the most discriminative and taxonomically useful descriptors, which avoid redundancy. They were also selected for ease of observation, reliability of scoring, and, in the case of seed characters, agronomic and economic value [44–46]. When possible, the full complement of samples was collected for each accession at a given time, but in many cases, samples had to be collected over multiple years to obtain the full sample size. In these cases, sampling was conducted during the same season to preclude the effect of seasonality on phenotypic trait expression. The fruits characterized were the products of open pollination. These data are available online in the International Cocoa Germplasm Database (ICGD) (http://www.icgd.rdg.ac.uk/).

**Table 1:** Descriptors and sample sizes used for phenotypic characterisation.

Descriptor/trait	State [sample size for each trait]
1. Flower, anthocyanin intensity in column of pedicel (FL_PCOL_C)	1 = green, 2 = reddish, 3 = red [n = 10].
2. Flower, sepal length (mm) (FL_SEP_LEN)	[n = 10]
3. Flower, anthocyanin intensity on ligule (FL_LIG_COL)	0 = absent, 3 = slight, 5 = intermediate, 7 = intense [n = 10]
4. Flower, ligule width (mm) (FL_LIG_WID)	[n = 10]
5. Flower, anthocyanin intensity in filament (FL_FIL_COL)	0 = absent, 3 = slight, 5 = intermediate, 7 = intense [n = 10]
6. Flower, style length (mm) (FL_STY_LEN)	[n = 10]
7. **Flower, ovule number (FL_OV_NUM)**	[n = 10]
8. Fruit, shape (FR_POSH)	1 = oblong, 2 = elliptic, 3 = obovate, 4 = orbicular], 5 = oblate or other (specified). [n = 10
9. Fruit, basal constriction (FR_PBC)	0 = absent, 1 = slight, 2 = intermediate, 3 = strong, 4 = wide shoulder [n = 10]
10. Fruit, apex form (FR_PAF)	1 = attenuate, 2 = acute, 3 = obtuse, 4 = rounded, 5 = mammillate, 6 = indented [n = 10]
11. Fruit, surface texture (rugosity or degree of wartiness) (FR_PST)	0 = absent, 3 = slight, 5 = intermediate, 7 = intense [n = 10]
12. Fruit, surface anthocyanin intensity in mature ridges (FR_PRCM)	0 = absent, 3 = slight, 5 = intermediate, 7 = intense [n = 10]
13. Fruit, ridge disposition (FR_PFD)	1 = equidistant, 2 = paired [n = 10]
14. Fruit, primary ridge separation (FR_PFS)	1 = slight, 2 = intermediate, 3 = wide [n = 10]
**15. Fruit, pod wall hardness [n = 10] (FR_HH (qualitative); FR_QHH (quantitative))**	3 = ≤ 1.6 megapascals (MPa), 5 = > 1.6 MPa ≤ 2.0, MPa, 7 = > 2.0 MPa
16. Fruit, length (cm) (FR_POL)	[n = 10]
17. Fruit, width (cm) (FR_POW)	[n = 10]
18. Fruit length to width ratio (FR_PODL_W)	
**19. Seed, number (FR_BEN)**	[n = 10]
20. Seed, shape (FR_BES)	1 = oblong 2 = elliptic 3 = ovate
21. Seed, cotyledon colour (FR_BEC)	1 = white, 2 = grey, 3 = light purple, 4 = medium purple, 5 = dark purple, 6 = mottled [n = 40]
22. Wet bean mass (total) (g) (FR_WABW)	[n = 10]
**23. Cotyledon length (cm) (FR_BL)**	[n = 20]
**24. Cotyledon width (cm) (FR_BEW)**	[n = 20]
**25. Cotyledon mass (g) (FR_BW)**	[n = 20]
**26. Cotyledon length to width ratio (FR_BENL_W)**	
**27. Pod index**	The number of pods/fruits required to produce 1 kg of dried cocoa (1000/ (average dried cotyledon mass × average seed or bean number) [n = 10]

Traits of economic importance are highlighted in bold.

### Genotyping by sequencing

SNP markers that provide good coverage of the cacao genome [33] were employed in this study. The selection of 836 SNPs in coding sequences, which displayed significant similarity with known protein sequences, as described by Argout et al. [34], was carried out by Allegre et al. [33]. Illumina SNP genotyping was performed with the Illumina BeadArray platform at the French National Genotyping Centre (CNG, CEA-IG, Evry, France), according to the GoldenGate Assay manufacturer’s protocol. The genotype calling of each marker was verified using reference genotypes and filtered, as described by Argout et al. [34] and Allegre et al. [33]. The QualitySNP pipeline was used for detection of SNPs in the unigenes. All of the SNPs employed for genotyping have been identified in orthologous genes or gene families and this facilitates reference to genetic information, made available via the genome browser, CocoaGen DB (http://cocoa-genome-hub.southgreen.fr/jbrowse) and UniProtKB (https://www.uniprot.org/uniprot/).

### Statistical analyses

#### Phenotypic data analysis

Qualitative data such as fruit shape classes were first converted to binary form. The quantitative traits that were found to deviate from normality were log-transformed. Tests of normality, log transformation of data that were not normally distributed, derivation of descriptive statistics, ANOVA (General Linear Model for unbalanced data) of log-transformed data, and correlation analysis of the collated quantitative phenotypic data were performed using Minitab Version 18. The correlation matrices for the quantitative yield-related and anthocyanin pigmentation traits studied were calculated using command cor() and visualised with the ‘corrplot’ package in *R* [47]. Pearson and Spearman correlation coefficients were derived for the continuous and interval data, respectively.

#### Determining associations between molecular polymorphisms and quantitative traits

*Population structure*. Population structure was determined to allow estimation of marker-trait associations without including spurious associations [48]. This was necessary to satisfy the independence assumption under the null hypothesis on which the marker-trait association was based [49]. The Bayesian clustering software, *STRUCTURE*, [49–53] was employed for this purpose. It defined the inferred ancestry of individuals, studied as coefficients of the individuals across sub-populations. Individuals with coefficients of membership of less than 0.7 were classified as admixed. The allele frequencies correlated model used Markov Chain Monte Carlo (MCMC) simulations to estimate the group (cluster, *K*) membership of each individual studied, assuming Hardy-Weinberg and linkage equilibrium within groups, random mating within populations and free recombination between loci [49].

Multi-locus genotype data for 200 SNPs, distributed over all 10 chromosomes, with minor allele frequency (MAF) greater than 0.05 and low missing values (less than 10%) were analysed in *STRUCTURE* to describe and visualize population structure, based on allele frequencies of the data. The optimum *K* value that best defined the population structure was identified using the admixture model of ancestry, assuming correlated allele frequencies for *K* = 2 to 15 with 150,000 iterations during the burn-in period, 150,000 Markov Chain Monte Carlo repetitions and 10 independent runs for each genetic sub-population (*K*2- *K*15).

The *STRUCTURE* outputs were analysed to infer optimal *K* based on the method described by Pritchard [49]. The optimal *K* was chosen by plotting the log probability of the data, Pr (X | *K*), against a range of *K* values and selecting the one after which the curve formed a plateau, while also considering the consistency of the groupings across multiple runs with the same *K*. Runs for which the variance was not homogeneous with variances of the other runs with the same *K* value were excluded.

The population structure of the 421 accessions studied was visualized using DARwin (Dissimilarity Analysis and Representation for Windows) version 6 (http://darwin.cirad.fr) [54]. DARwin was used to estimate pairwise Jaccard’s genetic dissimilarity indices using the 200 SNP markers employed in *STRUCTURE* Analysis. A tree was constructed by clustering accessions, based on a dissimilarity matrix using the Unweighted Pair Group Method with Arithmetic Mean (UPGMA). Clade strength in the dendrogram was tested using 1000 bootstraps. This tree was rendered with iTOL (https://itol.embl.de/).

*Genome-wide Association Studies (GWAS)*. *TASSEL* [55], version 5.2.50 for Windows, was employed to conduct GWAS and the results for yield-related traits were compared with those derived with GAPIT FarmCPU (Fixed and random model for circulating probability unification) [56]. In *TASSEL*, the Mixed Linear model (MLM) tested fixed effects to determine associations between genetic sites and phenotypes. FarmCPU was employed to verify the results generated with *TASSEL* MLM since it caters for random as well as fixed effects in the MLM, and removes false positive and negative associations [56].

MLM in *TASSEL* was used to correct covariances due to relatedness at the population level between genotypes (due to population structure) as well as co-ancestry (kinship) or identity by descent. The inclusion of the *K* (kinship) matrix allowed the inclusion of multiple backgrounds QTL as a random factor in the mixed linear model, as explained by Henderson [57]. The scaled, centred ‘IBS’ method, described by Endelman and Jannink [58], was used to generate the Kinship matrix of relationships among genotypes using *TASSEL*.

The statistical model used for the TASSEL MLM is as follows:

Y=Xβ+Zu+e

Where Y is the vector of observations, β an unknown vector of fixed effects including genetic marker and population structure (Q); u is an unknown vector of random additive genetic effects from multiple background QTL for the individuals; ***X*** and ***Z*** are the known design matrices; and ***e*** is the unobserved vector of random residuals.

Each marker allele was fit as a separate class with heterozygotes fit as an additional class. The minor allele frequency (MAF) was set to > 0.05. SNPs with more than 14% missing values were removed and imputation was performed to replace any other missing values using the Euclidean distance measure in the *k*-nearest-neighbour algorithm [59] generated for the 10 nearest neighbours [55]. Phenotypic data for 75 accessions were imputed based on their genetic profiles.

The stringent Bonferroni correction test to cater for multiple testing [55] was applied to the derived *P* values to test the significance of the associations between traits and markers. Manhattan plots, generated in *R* [47], were also used to check for evidence of *P* value inflation and to identify significant MTAs based on *TASSEL* MLM.

Simple M in *R* [47] was used to determine the number of independent markers that should be used in testing to avoid the penalty of the stringent Bonferroni correction test, as prescribed by Gao et al. [60]. There were 664 SNP markers that were independent.

The LD pattern was tested with 100,000 permutations in *TASSEL* to obtain *P* values for the tests. The correlation between alleles at two loci, *r*^*2*^, and the standardized disequilibrium coefficient for determining whether recombination or homoplasy had occurred between a pair of alleles, *D*^*2*^, were derived. Fisher’s exact test was calculated to compare alleles at any two loci. LD heatmap was used to scan for high linkage disequilibrium within chromosomes based on *r*^*2*^ values. High LD was characterized by many red squares in the heatmap generated. Many red blocks together would be inferred as corresponding to haplotype blocks [61, 62].

Loess regression was performed in *R* [47] to investigate LD decay by plotting the *r*^2^ values as a function of genetic distance in megabases (Mb). The decay was plotted to show the points representing the distance on each chromosome, at which the mean value of *r*^*2*^ decreased to half of the maximum value.

Quantile-quantile plots were also generated in *R* [47] to search for evidence of bias in the GWAS, such as due to genotyping artifacts, and to discern the extent to which the observed distribution of the test statistic followed the expected (null) distribution. The proportion of phenotypic variance explained by a marker was determined by the square of the partial correlation coefficient (R^2^).

#### Genomic prediction

The predictive breeding value (GEBV) (along with the predictive error variance (PEV)) for each phenotypic trait was derived using ridge regression in *TASSEL* 5.2.50 software, which performed multiple correlation tests to compute correspondence between genotype and phenotype and accounted for collinearity to avoid bias. The genotypic data (737 SNPs in alpha format) were loaded, and kinship analysis was performed to generate an Identity by Descent (IBD) file. The phenotypes and *K* matrix were selected, and the genomic selection routine was run with 5-fold cross validation with 100 iterations.

#### Detecting candidate genes located within marker-trait association zones

The identification of putative candidate genes was done using the latest cocoa genome sequence (*T*. *cacao* Criollo genome version 2) [29] available on the Cocoa Genome hub (https://cocoa-genome-hub.southgreen.fr/jbrowse). The biological functions of genes/transcripts close to the significant SNPs were determined by searching the SNP sequences with proteins related to seed development on the cocoa genome after referring to *UniProtKB* 2021 to identify such proteins. Flanking regions upstream and downstream of stable MTAs were selected to locate putative candidate genes. The lengths of the flanking regions were determined based on the linkage disequilibrium decay intervals on the respective chromosomes. For chromosomes 3, 5, 6 and 8, this was over a distance of 2.5 Mb upstream or downstream of the significant MTAs. It was over a distance of 5 Mb for chromosome 1, 1.6 Mb for chromosome 4, 0.87 Mb for chromosome 7 and 0.86 Mb for chromosome 9. A physical map, showing the position of annotated putative candidate genes that co-localised with SNP markers associated with traits of interest (MTAs), was then constructed using *SpiderMap* (Rami, 2007 unpublished, Spidermap v1.7.1, free software, CIRAD).

## Results

### Phenotypic data analysis

The phenotypic data for the fully characterized accessions are presented in S2 Table (http://dx.doi.org/10.13140/RG.2.2.29601.48484). The tests of normality results are presented in S3 Table (http://dx.doi.org/10.13140/RG.2.2.22890.59849). Tests of normality indicated significant deviation from normality for fruit length, width, fruit length to width ratio, total fresh seed mass, seed number, individual seed (cotyledon) mass and width, seed length to width ratio, pod index, ovule number, sepal length and width, and style length. Results of tests of normality performed on the natural log transformed values of fruit and seed quantitative traits indicated that natural log transformation corrected for the deviation from normality of these traits (S3 Table http://dx.doi.org/10.13140/RG.2.2.22890.59849). The phenotypic data were thus natural log transformed to conduct GWAS. Untransformed data were also subjected to GWAS for comparison.

**Fig 1:**
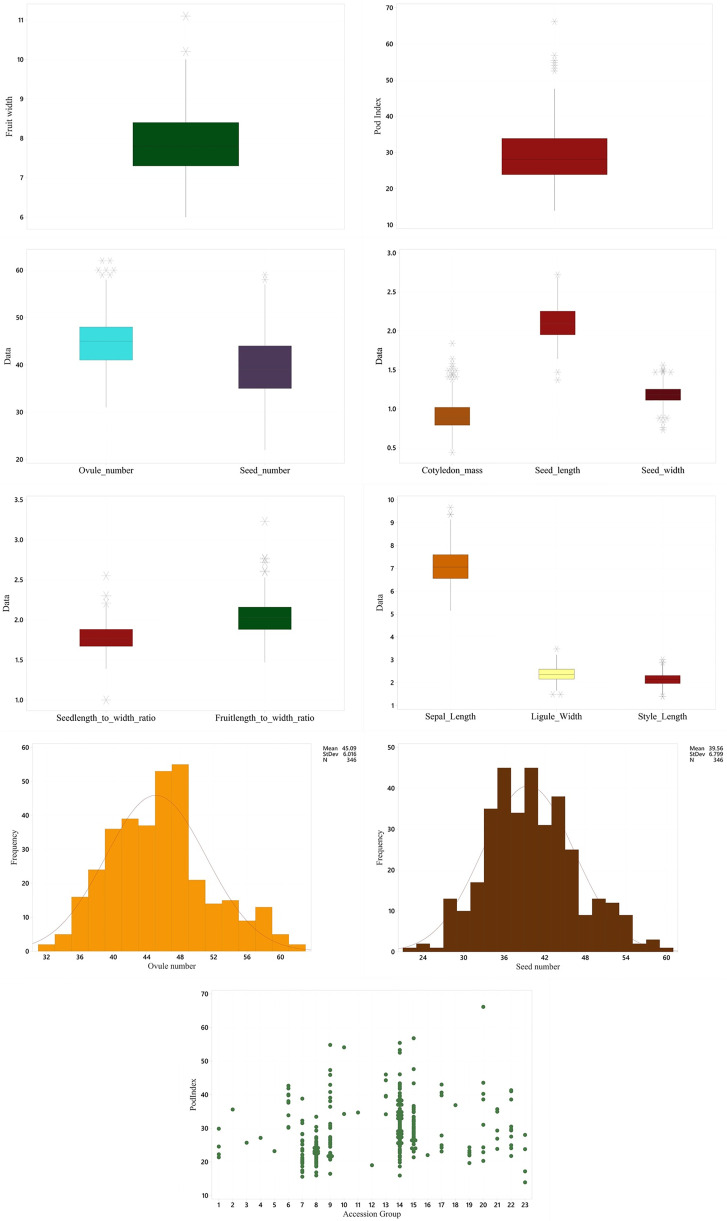
Box and whisker plots, histograms and individual value plot for pod index showing variation in the yield-related traits. The asterisks in the box and whisker plots represent outliers.

The large phenotypic variation expressed in this panel of cacao genotypes, based on the coefficients of variation (S4 Table http://dx.doi.org/10.13140/RG.2.2.36312.37128), and evident in Fig 1 and S1 Fig (http://dx.doi.org/10.13140/RG.2.2.24148.88966), indicated a diverse genetic background, which was suitable for GWAS. The results of ANOVA for yield-related traits are presented in S5 Table (http://dx.doi.org/10.13140/RG.2.2.17438.00328). All of the traits differed significantly between wild and cultivated germplasm except for seed (cotyledon) length to width ratio and fruit length.

**Fig 2:**
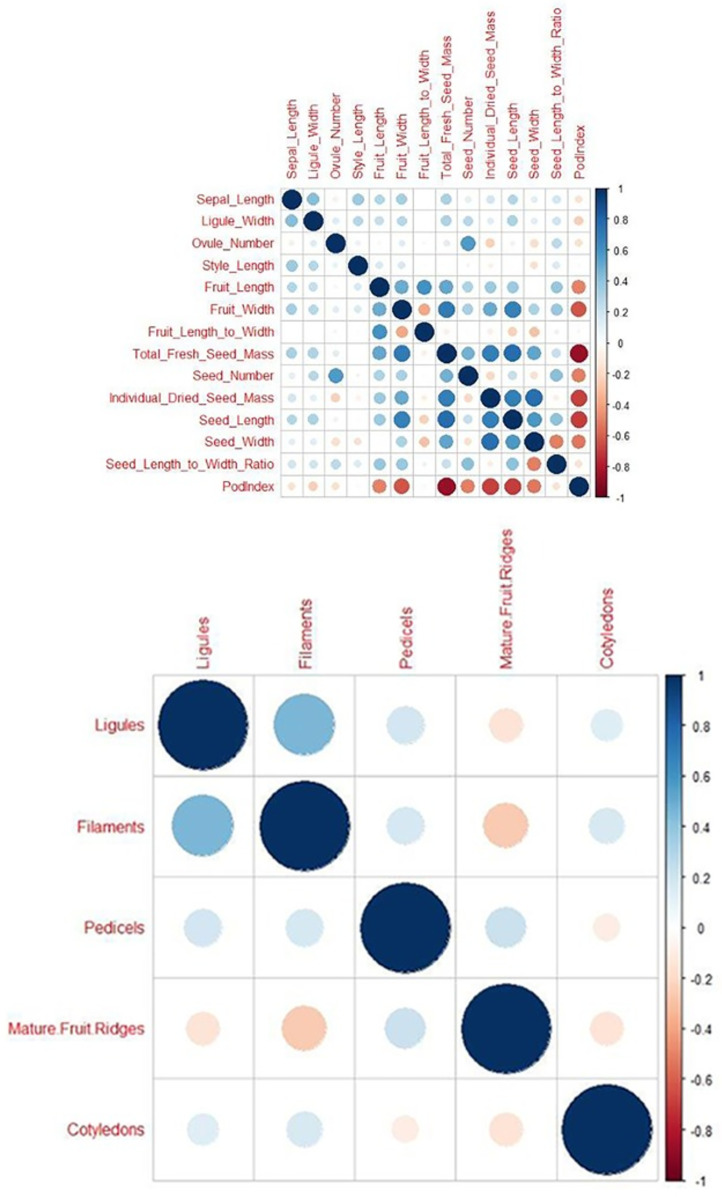
Correlograms showing Pearson correlations for quantitative traits and Spearman correlations for anthocyanin intensity in various plant organs. Positive correlations are displayed as blue circles and negative correlations as orange circles. The sizes of the circles are proportional to the correlation coefficients. The plant organs for which anthocyanin intensity was measured were the flower ligule, filament and pedicel, and mature fruit ridges and seed cotyledons).

Correlation analysis revealed the interdependence of traits on one another. Of the 91 Pearson correlation coefficients, *r*, calculated for the quantitative traits, only 20 were not significant (*P* ≥ 0.05). It is noteworthy that the yield-related traits, seed number, seed/cotyledon mass and seed dimensions, were highly correlated (*P* ≤ 0.001). Correlations of the yield-related traits for 346 cacao accessions are presented in the Correlogram (Fig 2) Seed number was negatively correlated (*r* = -0.205***) with individual dried cotyledon mass. Seed/cotyledon length and width were positively correlated with individual dried seed/cotyledon mass (*r* = 0.688*** and 0.751***, respectively). This suggests that the former traits can be used as indicators of the latter, a significant finding.

There was also a strong correlation (0.568***) between ovule number and seed number that justifies prediction of the latter using the former when fruits are unavailable. The correlations of fruit width with pod index and seed length, *r* = -0.621*** and 0.680***, respectively, were also of interest.

Correlations for anthocyanin intensity in the various plant organs are presented in Fig 2. All of these correlations, based on the Spearman correlation coefficient (r_s_), were significant (*P* ≤ 0.05) except for the anthocyanin pigment concentration in the pedicel and anthocyanin pigmentation of the cotyledon (*r* = -0.097). It was notable that the correlations involving mature fruit surface (ridges) anthocyanin intensity and that in the ligule and filament of the flower and in the seed/cotyledon were all significantly (*P* ≤ 0.05) negative (*r* = -0.150, -0.257, -0.147, respectively).

### Population structure

**Fig 3:**
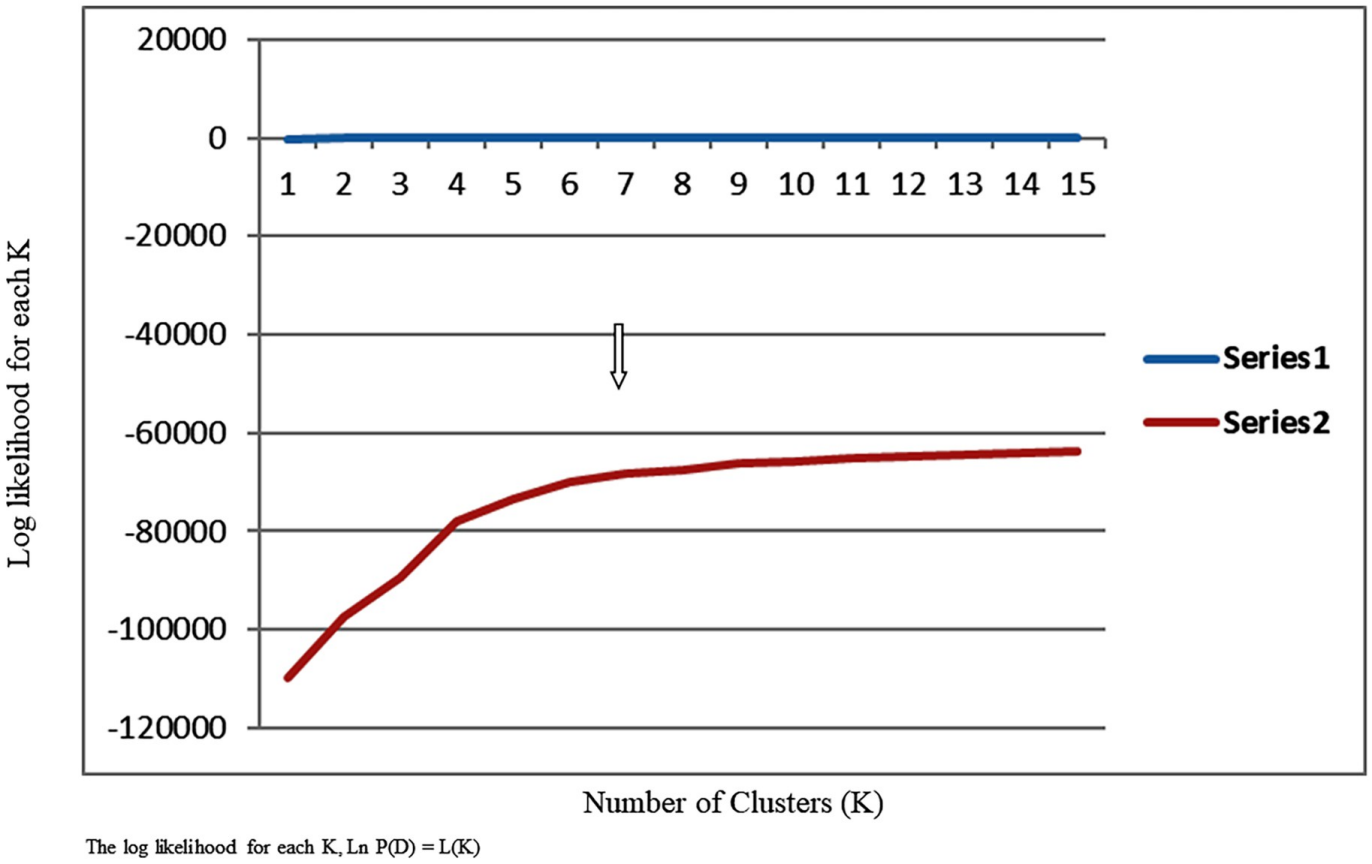
Plot of log of K versus number of clusters based on STRUCTURE analysis. Analysis of population structure of 421 cacao accessions using STRUCTURE—estimated Ln*P*(K) of possible clusters (*K*) from 2 to 15. When *K* is approaching a true value, L(*K*) plateaus (or continues increasing slightly).

Ten replicate runs of models using genotypic clusters (*K*) from 2 to 15 confirmed that *K* = 7 had the highest log-likelihood probability (log Pr (X | *K*) versus *K*) (Fig 3).

**Fig 4:**
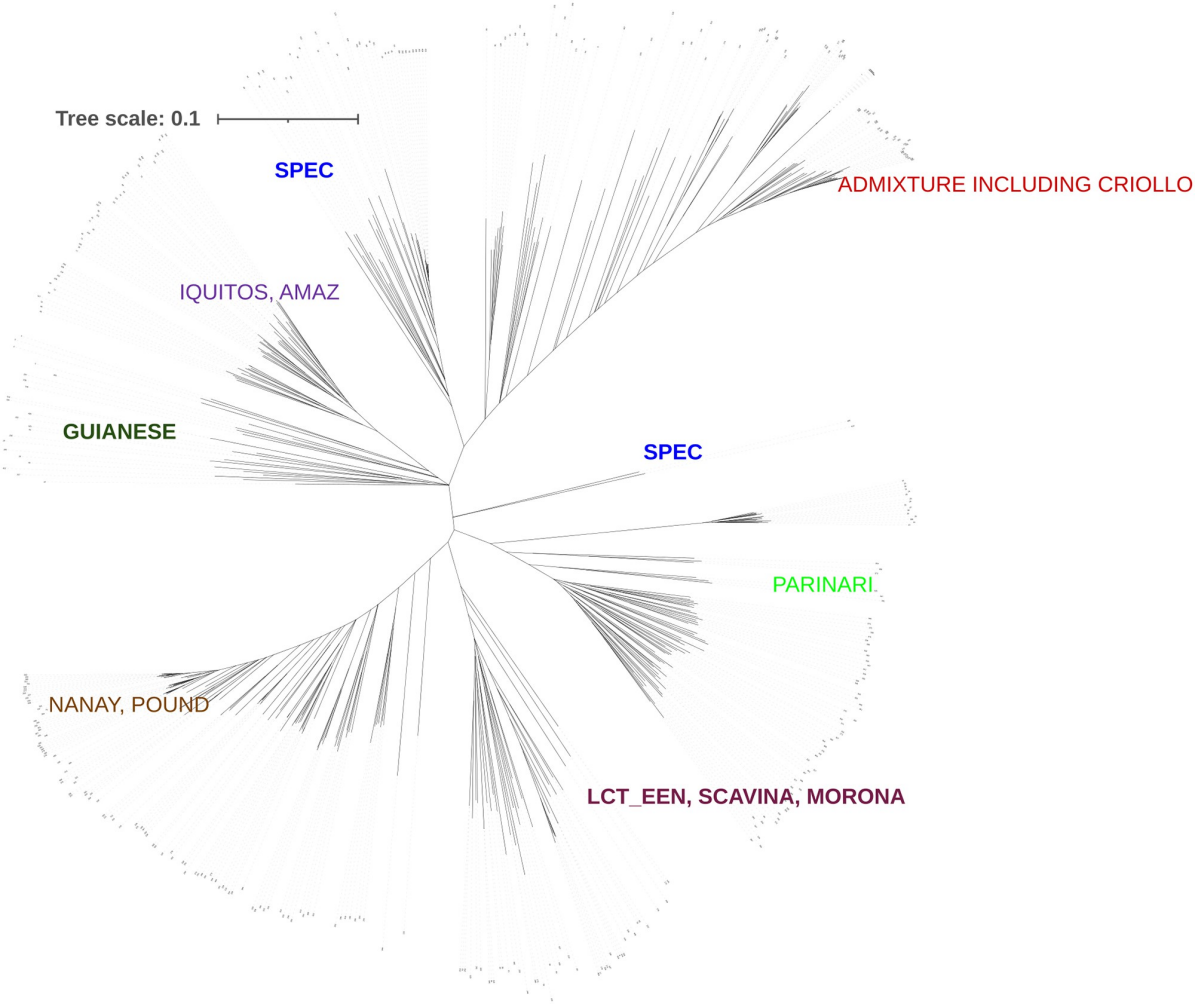
Neighbour-joining tree based on UPGMA of 421 cacao genotypes. The tree was generated in DARwin Version 6 and rendered in iTOL version 6 (https://itol.embl.de/). Seven admixed groups are evident.

The population structure analysis revealed that 74% of the accessions could be stratified into seven sub-populations, while 26% could be regarded as admixtures. The constitution of the seven genetic clusters is presented in S6 Table (http://dx.doi.org/10.13140/RG.2.2.27504.33282). The IMC and AMAZ groups were clustered together as were also the SCA, MO and LCTEEN groups (S6 Table http://dx.doi.org/10.13140/RG.2.2.27504.33282). Several Upper Amazon accessions were genotyped as Trinitario, and are putatively mislabelled. The genetic diversity of the accessions studied is represented in the neighbour-joining tree in Fig 4, which depicts the seven clusters differentiated. (One SPEC accession was not clustered with other accessions).

### Linkage disequilibrium (LD)

**Fig 5:**
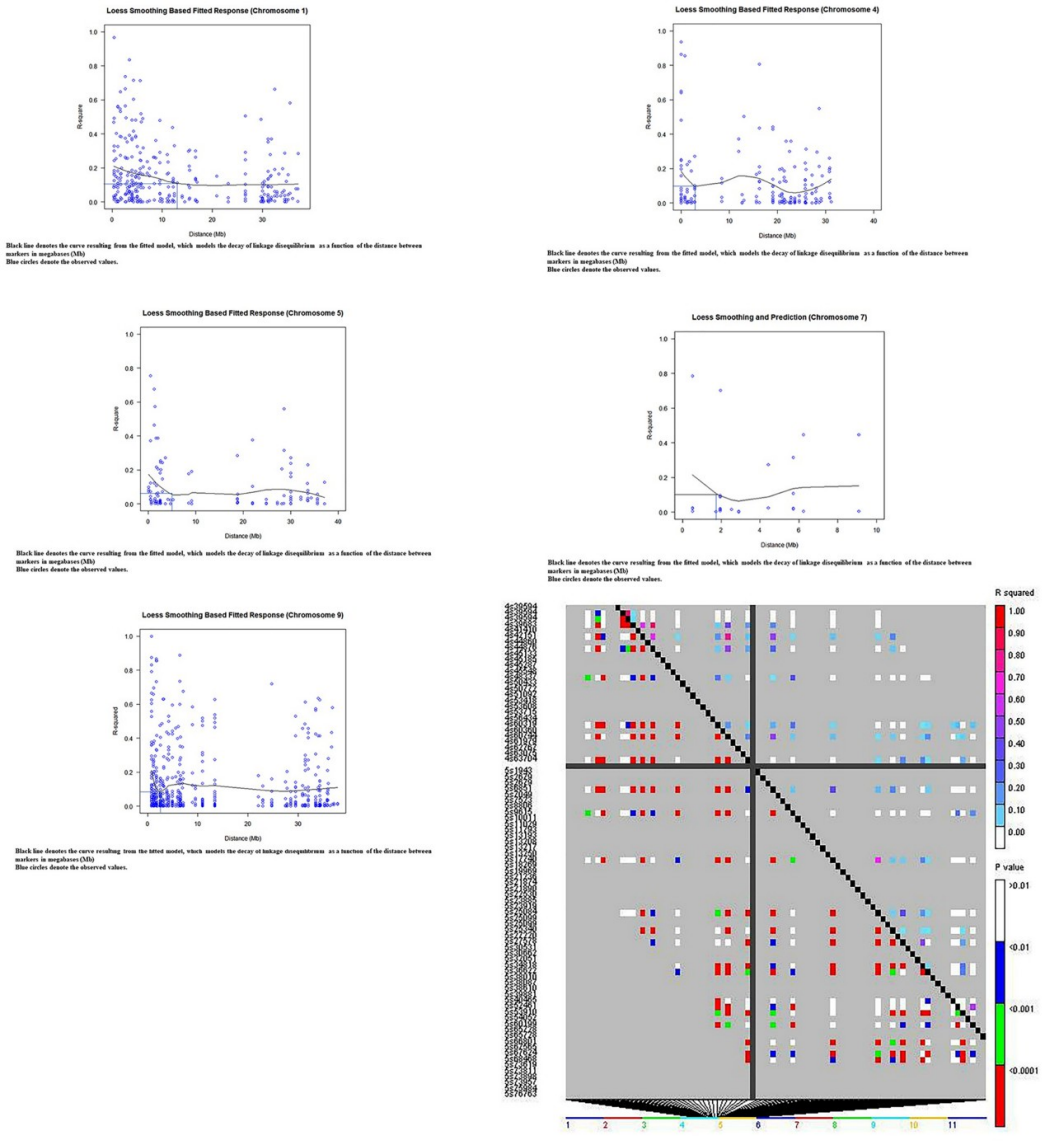
Plots modelling the decay in pairwise linkage disequilibrium coefficients (*r*^2^) as a function of the distance between markers in megabases (Mb). Plot of pairwise linkage disequilibrium coefficients (*r*^2^) on chromosome 1; Plot of pairwise linkage disequilibrium coefficients (*r*^2^) on chromosome 4; Plot of pairwise linkage disequilibrium coefficients (*r*^2^) on chromosome 5; Plot of pairwise linkage disequilibrium coefficients (*r*^2^) on chromosome 7; Plot of pairwise linkage disequilibrium coefficients (*r*^2^) on chromosome 9. Heatmap of linkage disequilibrium (*r*^2^) across the chromosomes 4 and 5 based on data for 421 cacao accessions genotyped using 612 filtered SNPs. Markers were ordered on the x and y axes in the Heatmap according to location along the chromosomes and each cell of the heatmap represents a single marker pair. The upper triangle, above the black diagonal on the heatmap, is colour-coded based on the *r*^2^ value between SNPs while colours depicted in the lower triangle are based on *P*-values for the corresponding *r*^2^ values.

*D*^*2*^ greater than 0.6, where *D*^*2*^ of 1 represents the highest amount of disequilibrium possible, was indicative of recombination or homoplasy between pairs of alleles (Table 2). With regard to the mean squared correlations of allele frequencies, *r*^*2*^ values, none were observed to be greater than 0.2 and the mean *r*^*2*^ across the 10 chromosomes was 0.1 (Table 2). Chromosomes 1 and 3 had mean *r*^*2*^ > 0.14. An average decay of *r*^2^ to 50% (to *r*^2^ = 0.1) over chromosomes 1, 4, 5, 7 and 9 occurred at a distance of 5.21 Mb (9 cM) (S7 Table http://dx.doi.org/10.13140/RG.2.2.20793.44647). LD decay plots, based on *r*^*2*^, are presented in Fig 5.

**Table 2:** Results of linkage disequilibrium analysis.

Variable	Chromosome	Total Count	Mean	SE Mean	StDev	Minimum	Maximum
*D* prime	1	325	0.6729	0.0165	0.2979	0.0012	1.000
	2	495	0.6387	0.0138	0.3074	0.0009	1.000
	3	1071	0.6300	0.0089	0.2930	0.0014	1.000
	4	1231	0.6053	0.0084	0.2958	0.0000	1.000
	5	1431	0.5672	0.0079	0.2998	0.0000	1.000
	6	812	0.6547	0.0101	0.2873	0.0004	1.000
	7	856	0.6244	0.0107	0.3118	0.0018	1.000
	8	1157	0.6407	0.0093	0.3155	0.0030	1.000
	9	5091	0.6223	0.0043	0.3080	0.0000	1.000
	10	1139	0.5530	0.0094	0.3156	0.0040	1.000
*r* ^*2*^	1	325	0.1456	0.0094	0.1699	0.0000	0.9657
	2	495	0.1239	0.0075	0.1661	0.0000	0.9542
	3	1071	0.1413	0.0052	0.1689	0.0000	0.8428
	4	1231	0.1084	0.0044	0.1559	0.0000	0.9346
	5	1431	0.0802	0.0031	0.1169	0.0000	0.7548
	6	812	0.0827	0.0044	0.1245	0.0000	0.7229
	7	856	0.0955	0.0047	0.1382	0.0000	0.7849
	8	1157	0.0679	0.0037	0.1255	0.0000	0.8633
	9	5091	0.0982	0.0021	0.1487	0.0000	1.0000
	10	1139	0.0618	0.0028	0.0959	0.0000	0.5690

SE–Standard error, StDev–Standard deviation.

The linkage disequilibrium (LD) decay patterns, observed for the chromosomes in this study population and a LD heat map (Fig 5, S7 Table http://dx.doi.org/10.13140/RG.2.2.20793.44647), were used to inform the process of searching for putative annotated candidate genes. JBrowse in version 2 of the Criollo B97-61/B2 genome (https://cocoa-genome-hub.southgreen.fr/) was used to locate candidate genes upstream or downstream of MTA zones. The search for putative candidate genes with relevant functional (biological) roles such as in seed development, identified in *UniProtKB*, was conducted within defined intervals, based on linkage disequilibrium decay, spanning MTAs on each chromosome (S7 Table http://dx.doi.org/10.13140/RG.2.2.20793.44647).

### Putatively robust marker-trait associations (MTAs)

**Fig 6:**
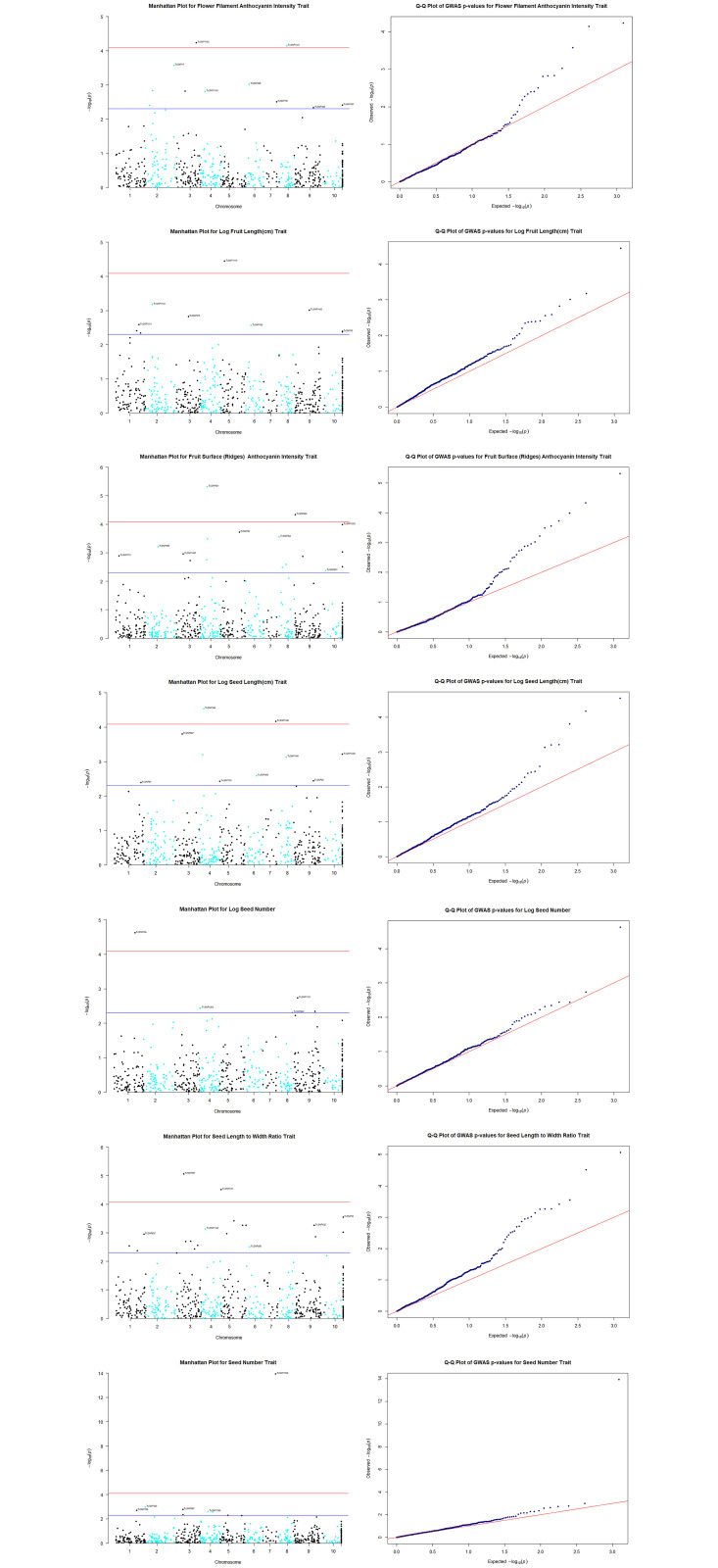
Manhattan plots from genome-wide association analysis. Genome-wide association plots across 8 cacao chromosomes for seven phenotypic traits that had statistically significant MTAs: filament anthocyanin intensity, fruit surface (ridges) anthocyanin intensity, log fruit length, log seed length, log seed number, seed length to width ratio, seed number.
Based on TASSEL version 5.2.50 MLM results for 421 cacao accessions (612 SNPs).Chromosome “11” was designated for unmapped SNP markers (some of which have recently been mapped).X- and Y-axes represent the SNP markers along each chromosome and the -log10(*P*-value), respectively.The red horizontal line corresponds to the Bonferonni significance threshold of *P*-values ≤ 8.17 × 10^−5^ (–log10 (P) = 4.088) and the blue line corresponds to a significance level of 0.005. Fig 6 Quantile–quantile plots of estimated−log10 (*P*) from genome-wide association studies using *TASSEL* MLM. Quantile–quantile plots of estimated−log10 (*P*) for filament anthocyanin intensity; Quantile–quantile plots of estimated−log10 (*P*) for fruit surface (ridges) anthocyanin intensity; Quantile–quantile plots of estimated−log10 (*P*) for log fruit length; Quantile–quantile plots of estimated−log10 (*P*) for log seed length; Quantile–quantile plots of estimated−log10 (*P*) for log seed number; Quantile–quantile plots of estimated−log10 (*P*) for seed length to width ratio; Quantile–quantile plots of estimated−log10 (*P*) for seed number. The plots provide no evidence of bias in the GWAS, such as due to genotyping artifacts, and display the extent to which the observed distribution of the test statistic followed the expected (null) distribution. The red line represents expected *P*-values with no associations.

The *TASSEL* MLM GWAS unravelled 17 significant associations. These were significant above the stringent Bonferroni threshold of *P* ≤ 8.17 × 10^−5^. There was a predominance of positive associations between highly heritable qualitative traits such as fruit shape, anthocyanin intensity on fruit surface, fruit apex form, and flower filament anthocyanin intensity and SNP markers (Table 3, S8 Table http://dx.doi.org/10.13140/RG.2.2.34215.21927). However, there were also some significant associations between SNPs and quantitative traits. Two of the seventeen highly significant (*P* ≤ 8.17 × 10^−5^) MTAs, which were identified with *TASSEL* MLM, were corroborated at this level of significance with GAPIT FarmCPU (Table 3). Interestingly, the most significant (*P* ≤ 1.15 × 10^−14^) MTA based on the *TASSEL* MLM, which was observed for seed number and TcSNP 1335 on chromosome 7, and another involving TcSNP 1335 and log seed length on chromosome 7 were not detected when the FarmCPU model was used. However, FarmCPU revealed a significant MTA involving TcSNP 1126 and seed length on chromosome 7. The highly significant MTA involving log seed number and TcSNP 785 on chromosome 1 (*P≤* 2.38 × 10^−05^) (Table 3) was also not verified with FarmCPU, but a MTA for TcSNP 67 and seed length was identified on chromosome 1 (*P* ≤ 0.005). A comparison of the outputs of GWAS based on *TASSEL* MLM and GAPIT FarmCPU indicated 14 common MTAs involving yield-related traits at *P* ≤ 0.003 (Table 3). Manhattan plots and associated Quantile-Quantile plots are presented for yield-related traits in Fig 6, based on *TASSEL* MLM.

**Table 3:** Most significant, yield-related and other marker-trait associations and variation explained.

Trait	TASSEL Marker	Chr	Position	TASSEL *F*	*TASSEL* MLM *P*	TASSEL Marker R^2^	FarmCPU *P*
Flower filament anthocyanin intensity	TcSNP1183	3	32,973,816	10.01	5.84E^-05^	0.049	NA
	TcSNP1441	8	25,585,284	9.8	7.14E^-05^	0.048	NA
Fruit surface (ridges) anthocyanin intensity	**TcSNP401**	4	20,485,872	12.73	4.78E^-06^	0.071	2.58798E-^09^
	TcSNP644	9	1,220,437	10.31	4.57E^-05^	0.059	NA
Fruit shape orbicular	TcSNP1353	3	34,216,420	19.96	6.73E^-09^	0.109	NA
	TcSNP1477	3	17,127,233	12.15	8.20E^-06^	0.069	NA
Fruit shape oblate	TcSNP1477	3	17,127,233	12.15	8.20E^-06^	0.069	NA
	TcSNP1353	3	34,216,420	19.96	6.73E^-09^	0.109	NA
Fruit length	TcSNP1110	5	1,969,054	16.79	5.29E^-05^	0.047	NA
Log fruit length	TcSNP1110	5	1,969,054	17.56	3.60E^-05^	0.049	NA
Log seed length	**TcSNP344**	4	8,445,308	18.01	2.88E^-05^	0.050	**1.51E-** ^**03**^
	**TcSNP953**	4	2,822,,152	11.10	**9.61E** ^**-04**^	0.034	**7.68E** ^**-03**^
	**TcSNP897**	3	23,362,296	8.46	**2.61E** ^**-04**^	0.051	**6.01E** ^**-03**^
	**TcSNP733**	5	2,070,839	8.39	**4.0 E** ^**-03**^	0.025	**1.71 E** ^**-03**^
	TcSNP1335	7	9,085,336	9.89	6.75E^-05^	0.055	
Seed length (cm)	**TcSNP733**	5	2,070,839	7.08	**8.2E** ^**-03**^	0.022	**6.75E** ^**-03**^
	**TcSNP344**	4	8,445,308	11.31	**8.66E** ^**-04**^	0.034	**7.49E** ^**-03**^
Seed length: width	TcSNP897	3	23,362,296	12.09	8.57E^-06^	0.067	
	**TcSNP733**	5	2,070,839	17.89	3.05E^-05^	0.051	1.12375E^-08^
Log seed length: width	TcSNP823	5	2,546,863	9.49	**9.97E** ^**-05**^	0.053	
	TcSNP841	5	36,814,269				9.71745E^-07^
Log seed number	TcSNP785	1	31,785,158	11	2.38E^-05^	0.059	
Seed number	TcSNP1335	7	9,085,336	35.51	1.15E^-14^	0.170	
	TcSNP1350	1	35,918,110				**1.33E** ^**-03**^
	TcSNP1160	4	21,847,012	116.1	**1.35E** ^**-04**^	0.261	
Log seed cotyledon mass	**TcSNP555**	4	29,189,575	12.22	**5.37E** ^**-04**^	0.037	**5.7E** ^**-04**^
	**TcSNP344**	4	8,445,308	8.79	**3.0 E** ^**-03**^	0.026	**9.9E** ^**-04**^
	TcSNP932	9	1,479,475				9.56678E^-05^
Seed cotyledon mass (g)	**TcSNP555**	4	29,189,575	9.41	**2.0 E** ^**-03**^	0.028	**1.01E** ^**-03**^
	TcSNP953	4	2,822,,152				**1.98E** ^**-02**^
	TcSNP733	5	2,070,839				4.27833E^-05^
Log seed width	**TcSNP555**	4	29,189,575	9.73	**1.9E** ^**-03**^	0.029	6.31401E^-05^
Seed width (cm)	**TcSNP555**	4	29,189575	11.34	**8.50E** ^**-04**^	0.034	4.57572E-^05^
Log Pod Index	**TcSNP555**	4	29,189575	9.35	**2.0 E** ^**-03**^	0.028	**3.33E** ^**-03**^
	**TcSNP642**	8	16,399,147	12.08	**5.77E** ^**-04**^	0.037	**1.09E** ^**-03**^
Pod Index	**TcSNP555**	4	29,189575	11.39	**8.24E** ^**-04**^	0.035	**1.09E** ^**-03**^
	**TcSNP642**	8	16,399,147	12.61	**4.39E** ^**-04**^	0.038	**1.02E** ^**-03**^

*P* values below the stringent Bonferroni correction threshold are in bold.

Chr = chromosome

Bonferroni threshold log10 8.17e^-05^ = -4.0877

Significant yield-related MTAs based on *TASSEL* MLM

Chr 7 seed number 1.15E^-14^ = -log 10 (*P*) 13.939

Chr 3 seed length to width 8.57E^-06^ = -log 10 (*P*) 5.067

Chr 4 log seed length 2.88E^-05^ = -log 10 (*P*) 4.541

Chr 1 log seed number 2.38E^-05^ = -log 10 (*P*) 4.623

Chr 5 seed length to width 3.05E^-05^ = -log 10 (*P*) 4.516

Chr 7 log seed length 6.75E^-05^ = -log 10 (*P*) 4.171

Chr 5 log fruit length 3.60 E^-05^ = -log 10 (*P*) 4.44

The relatively low number of SNPs significantly associated with phenotypic traits, 2.4% of all SNPs used, was probably partly due to the stringent statistical thresholds applied in this study. Consequently, the results were carefully scrutinized just below the level of significance to discern MTAs, which may also have valid functional (biological) significance and to preclude false negative MTAs associated with *TASSEL* MLM. The results of the GAPIT FarmCPU analysis were confirmatory for 15 significant MTAs, including 14 yield-related traits, found with *TASSEL* MLM. These were significant above the Bonferroni threshold in two cases and at *P* ≤ 0.003 in the others (Table 3, S8 Table http://dx.doi.org/10.13140/RG.2.2.34215.21927).

### Putative annotated candidate genes

Nine putative annotated candidate genes with functional roles related to seed development, lipid biosynthesis and transfer and carbohydrate transport were identified on chromosome 1 based on *TASSEL* MLM (Table 4). TcSNP 785, at position 31,785,158 bp on chromosome 1, was co-localized with gene *Tc01v2_g025880*, which is functionally significant since it encodes protein disulfide isomerase that may be required for proper pollen development, ovule fertilization and embryo development (Table 4). This finding was not corroborated with the FarmCPU analysis, which only detected TcSNP 67 to be significantly associated with seed length on chromosome 1 (at position 35,469,159 bp). The functional protein co-localised with TcSNP 67 was 40S ribosomal protein S13.

**Table 4:** Genes co-localized with SNP markers significantly associated with phenotypic traits.

Trait	Marker	Chrom-osome	Physical map location	Co-localized gene/protein with a functional role for this study (location in parentheses)	Gene ontology (annotated description)
Log Seed number* (*TASSEL* MLM)	TcSNP785	1	31,785,158	*Tc01v2_g022850 Bifunctional inhibitor/lipid-transfer protein/seed storage 2S albumin superfamily protein* (29,410,506..29,412,056);	**Lipid transport**, involved in the pathway triacylglycerol biosynthesis, which is part of Glycerolipid metabolism, enables lipid binding;
				*Tc01v2_g023950* Sugar transporter ERD6-like 16 ((30,357,454..30,361,561);	Carbohydrate transport;
				*Tc01v2_g024420* Zinc finger CCCH domain-containing protein 32 (30,688,999..30,694,741);	DNA binding, metal ion binding, embryo development ending in seed dormancy;
				*Tc01v2_g025880* Probable protein disulfide-isomerase A6 (31,781,941..31,785,655);	Protein disulfide isomerase that may be required **for proper pollen development, ovule fertilization and embryo development**;
				*Tc01v2_g027650* WAT1-related protein At3g30340 (32,937,915–32,939,711);	Seed development, L-glutamate transport across plasma membrane;
				*Tc01v2-gO27930* Transducin family protein/WD-40 repeat family protein isoform 2 (33,105,658–33,117,406);	Embryo seed development ending in seed dormancy, embryonic pattern specification;
				*Tc01v2_g028250* Pentatricopeptide repeat-containing protein At5g16420, mitochondrial (33,378,320..33,380,942);	Embryo development ending in seed dormancy;
				*Tc01v2_g028470 Tetratricopeptide repeat-like superfamily protein*, *putative* (33,522,682–33,527,585);	Embryo development ending in seed dormancy;
				*Tc01v2_g028630* E3 ubiquitin-protein ligase SGR9%2C amyloplastic (33,651,107–33,655,106)	Seed development
Fruit shape- orbicular and oblate* (*TASSEL* MLM)	TcSNP1353	3	34,216,420	*Tc03v2_g017890* WAT1-related protein At3g30340 (30,725,570..30,728,183);	Seed development, L-glutamate transport across plasma membrane;
				*Tc03v2_g018590* Putative MADS-box protein FBP24 (31,145,695..31,147,439);	Protein dimerization activity; Seed development;
				Tc03cons_t027110.1 Short-chain dehydrogenase TIC 32, chloroplastic (34,213,998..34,217,670)	Involved in protein precursor import into chloroplasts
Fruit shape orbicular and oblate* (*TASSEL* MLM)	TcSNP1477	3	17,127,233	*Tc03v2_g005890* Ubiquitin carboxyl-terminal hydrolase-related protein isoform 1 (16,583,367..16,592,673);	Seed development;
				Tc03cons_t008500.1 Homeobox protein%2C putative isoform 2 (17,126,096..17,128,183)	Positive regulation of transcription, DNA-templated. Sequence-specific DNA binding
**Seed length to width ratio (***TASSEL* MLM) **Log seed length** (*TASSEL* MLM and FarmCPU)	TcSNP897	3	23,362,296	*Tc03v2_g006940* F-box/kelch-repeat protein At1g74510 (20,863,889..20,866,720);	Biological clock regulation, photomorphogenesis, phenylpropanoid and pigmentation biosynthesis, and **biotic stress responses**;
				*Tc03v2_g007750* Pentatricopeptide repeat-containing protein At5g61370, mitochondrial (22,342,896..22,352,160);	Embryo development ending in seed dormancy;
				*Tc03v2_g007800* Formin-like protein (22,381,761..22,395,019); *Tc03v2_g008070* Diacylglycerol kinase 1 (22,709,997..22,716,468);	Seed morphogenesis; Diacylglycerol kinase is a lipid kinase converting **diacylglycerol** to phosphatidic acid, and regulates many enzymes including protein kinase C, phosphatidylinositol 4-phosphate 5-kinase;
				*Tc03v2_g008980* Tetratricopeptide repeat-like superfamily protein, putative (24,417,524..24,420,534);	Embryo development ending in seed dormancy;
				*Tc03v2_g009010* Transducin family protein / WD-40 repeat family protein isoform 1 (24,440,368..24,446,585);	Embryo development ending in seed dormancy, embryonic pattern specification;
				*Tc03v2_g009610* E3 ubiquitin-protein ligase RGLG2 (25,109,952..25,116,068)	Seed development
Log seed length* (*TASSEL* MLM and FarmCPU)	TcSNP344	4	8,445,308	*Tc04v2_g005270* Transducin family protein / WD-40 repeat family protein, putative isoform 1 (7,911,634..7,917,242);	Embryo development ending in seed dormancy, embryonic pattern specification;
				*Tc04cons_t006580*.*1* Serine/threonine-protein kinase At5g01020 (8,442,189..8,445,924)	ATP binding, protein kinase activity; **seed size control**. (Reference: Li, N.; Xu, R.; Li, Y. ***Molecular Networks of Seed Size Control in Plants***. Annu. Rev. Plant Biol. 2019, 70, 435–463.) [102]
Fruit surface anthocyanin intensity* (*TASSEL* MLM and FarmCPU)	TcSNP401	4	20,485,872	*Tc00_t058610* (mRNA) Putative MYB-related protein 308 (20,286,348..20,287,312); Tc04cons_t012890.1 (mRNA) *Tc04v2_g009950* Transcription repressor MYB 6 (20,286,346..20,287,946); *Tc00_t050170* Type mRNA Transcription repressor MYB 6 (20,287,465..20,287,934)	Cell differentiation; **anthocyanin synthesis**.
Pod index (*TASSEL* MLM)	TcSNP667	4	26,469,745	*Tc04v2_g015140* Transducin/WD40 repeat-like superfamily protein isoform 1; (25,270,128..25,276,905);	Embryo seed development ending in seed dormancy;
				*Tc04v2_g015950* ARM repeat superfamily protein, putative (25,885,196..25,905,106);	Embryo seed development ending in seed dormancy;
				*Tc04v2_g015970* Putative protein FAR1-RELATED SEQUENCE 10 (25,915,934..25,920,963);	Any process that modulates the frequency, rate or extent of cellular DNA-templated transcription;
				*Tc04v2_g015990* Zinc finger CCCH domain-containing protein 1 (25,930,603..25,933,222);	Embryo seed development ending in seed dormancy, DNA binding;
				*Tc04v2_g016720* Putative E3 ubiquitin-protein ligase PRT1 (26,481,865..26,486,773)	Seed development;
				*Tc04v2_g018550* Pentatricopeptide repeat-containing protein At1g25360 (27,636,000..27,638,950);	Embryo development ending in seed dormancy;
				*Tc04v2_g019240* Bifunctional inhibitor/lipid-transfer protein/seed storage 2S albumin superfamily protein, putative (28,057,429..28,058,701	Enables lipid binding, involved in **lipid transport**
Pod index (*TASSEL* MLM and FarmCPU)	TcSNP555	4	29,189,575	*Tc04v2_g019280* Vicilin (28,070,761..28,073,194);	**Storage protein**;
				*Tc04v2_g019640* Pentatricopeptide repeat-containing protein At5g43790 (28,325,040..28,327,111);	Embryo development ending in seed dormancy;
				*Tc04v2_g020060* Putative E3 ubiquitin protein ligase DRIP2 (28,617,769..28,622,712);	**Protein ubiquitination; response to water deprivation**;
				*Tc04cons_t025060*.*1* (mRNA) Glutathione transferase GST 23 (29,188,779..29,189,950);	Modulates various aspects of plant development, including growth, by affecting glutathione pools. **Negative regulation of response to water deprivation. Important role in seed development and seed size/mass determination (in chickpea)**;
				*Tc04v2_g021130* Transducin/WD40 repeat-like superfamily protein, putative isoform 1 (29,341,558..29,345,568);	Embryo development ending in seed dormancy, embryonic pattern specification
				*Tc04v2_g022420* Sugar transporter ERD6-like 16 (30,034,603..30,038,332);	**Carbohydrate transport**;
				*Tc04v2_g023430* ARM repeat superfamily protein (30,600,460..30,602,164);	Embryo seed development ending in seed dormancy;
				*Tc04v2_g023630* Bidirectional sugar transporter SWEET17 (30,712,418..30,715,479)	**Carbohydrate transport**; fructose export from vacuole to cytoplasm
Seed number (*TASSEL* MLM)	TcSNP1160	4	21,847,012	*Tc04v2_g010050* Putative E3 ubiquitin-protein ligase SINA-like 10 (20,474,407..20,478,158);	Seed development;
				*Tc04v2_g010810* Pentatricopeptide repeat-containing protein At3g12770 (21,341,408..21,344,118);	Embryo development ending in seed dormancy;
				*Tc04cons_t014430*.*1 (mRNA)* 14-3-3-like protein (21,845,881..21,849,187);	Positive regulation of protein catabolic process. **(It is also involved in stress response and to presence of cadmium ions—not a functional role in this study, but noteworthy**);
				*Tc04v2_g011630* Putative Late embryogenesis abundant protein 2 (22,388,936..22,390,194);	Embryo development ending in seed dormancy and response to water deprivation;
				*Tc04v2_g013080* Formin-like protein (23,585,867..23,592,071)	Seed morphogenesis
Log seed length (pod index, seed length, seed width at level of significance just below the Bonferroni threshold) (*TASSEL* MLM); and cotyledon mass (FarmCPU)	TcSNP953	4	2,822,152	Acyltransferase-like protein *At3g26840%2C chloroplastic* (2,812,655.. 2,818,423)	**Controls seed mass and oil content** in Assembly cotton 46 (*Gossypium* spp.), (involved in seed storage (globulins) and seed size)
				*Tc04v2_g003740* Casp-like protein 1F2 (2,821,625..2,823,513)	Iron and sulphur cluster binding, cellular anatomical entity
				*Tc04v2_g001980* Glycerol-3-phosphate dehydrogenase [NAD(+)] (1,371,892…1,375,324);	**Fatty acid biosynthetic process**;
				*Tc04v2_g003620* Glycerophosphodiester phosphodiesterase GDPD1, chloroplastic (2,733,578..2,736,152);	Lipid metabolic process;
				*Tc04v2_g003890* Pentatricopeptide repeat-containing protein At3g26782, mitochondrial (2,993,254..2,997,100)	Embryo development ending in seed dormancy;
				*Tc04v2_g004200* PIN domain-like family protein, putative isoform 1 (3,219,943..3,222,504);	Embryo seed development ending in seed dormancy;
				*Tc04v2_g004410* Stearoyl-[acyl-carrier-protein] 9-desaturase, chloroplastic (3,404,981..3,407,629 (- strand);	**Fatty acid biosynthetic process**, fatty acid metabolic process;
				*Tc04v2_g004650* ARM repeat superfamily protein isoform 1 (3,949,150..3,958,576)	Embryo seed development ending in seed dormancy
Seed length to width ratio* (*TASSEL* MLM and FarmCPU)	TcSNP733	5	2,070,839	*Tc05v2_g001050* ARM repeat superfamily protein isoform 1 (543,066..552,891);	Embryo seed development ending in seed dormancy;
				*Tc05v2_g001240* Transducin family protein / WD-40 repeat family protein%2C putative isoform 2 (643,906..651,703);	Embryo development ending in seed dormancy, embryonic pattern specification;
				*Tc05v2_g001740* Glycerol-3-phosphate 2-O-acyltransferase 6 (930,910..933,467);	Rate-limiting enzyme in the *de novo* pathway of **glycerolipid synthesis**. It catalyzes the conversion of glycerol-3-phosphate and long-chain acyl- CoA to lysophosphatidic acid;
				*Tc05v2_g001860* Pentatricopeptide repeat-containing protein At3g09060 (1,003,722..1,006,767);	Embryo development ending in seed dormancy;
				*Tc05v2_g001880* Transducin/WD40 repeat-like superfamily protein%2C putative (1,015,833..1,026,345);	Embryo development ending in seed dormancy, embryonic pattern specification;
				*Tc05v2_g003440* E3 ubiquitin-protein ligase PRT6 (1,796,537..1,810,528);	Seed development;
				Tc05cons_t003970.1 Soluble inorganic pyrophosphatase 4 (2,070,666..2,074,083)	Phosphate-containing compound metabolic process. Important for development (**also stress resistance in plants, responds to cadmium ion, not a functional role in this study, but of interest)**
Log seed length to width ratio* (*TASSEL* MLM)	TcSNP823	5	2,546,863	*Tc05v2_g005120* Probable E3 ubiquitin-protein ligase LOG2 (2,760,099..2,763,370)	Seed development;
Seed number and log seed length* (*TASSEL* MLM)	TcSNP1335	7	9,085,336	*Tc07v2_g010700* **Bidirectional sugar transporter SWEET2** (8,563,273..8,566,205);	**Carbohydrate transport, distribution and storage in seeds**;
				*Tc07v2_g011310* Pentatricopeptide repeat-containing protein At3g14580, mitochondrial (9,120,467..9,122,062);	Embryo development ending in seed dormancy;
				*Tc07v2_g011610* Putative VQ motif-containing protein 10 (9,576,828..9,577,776)	Endosperm development, regulation of seed growth;
				*Tc07_t012010* protein coding Putative 60S ribosomal protein L18a-like protein (9,081,512..9,085,643)	Ribonucleoprotein, Ribosomal protein
Pod index (*TASSEL* MLM and FarmCPU)	TcSNP642	8	16,399,147	*Tc08v2_g013700* Tetratricopeptide repeat-containing protein, putative isoform 1 (14,427,929..14,450,269);	Embryo development ending in seed dormancy;
				*Tc08v2_g014110* Pentatricopeptide repeat-containing protein At1g71460, chloroplastic (14,862,384..14,867,389);	Embryo development ending in seed dormancy;
				*Tc08v2_g015360* Protein disulfide-isomerase 5–2 (16,765,487..16,772,516)	May be required for proper pollen development, ovule fertilization and embryo development

*MTAs (above the Bonferroni threshold) based on *TASSEL* MLM with 612 SNPs are denoted by an asterisk (*).

There are 109 putative candidate genes associated with cacao seed development. Twelve were identified in this study (https://www.uniprot.org/uniprot/?query=cocoa+seed+development&sort=score).

Approximately 40 putative candidate genes that encode embryo and seed development, protein synthesis, carbohydrate transport and lipid biosynthesis, metabolism and transport were identified in this study.

On chromosome 3, 11 putative candidate genes were detected with *TASSEL* MLM. They encode traits associated with seed development and lipid accumulation (Table 4).

Associations based on *TASSEL* MLM between log seed length and TcSNP 953 (*P* ≤ 9.61 × 10^−04^) as well as with log seed length and TcSNP344 (*P* ≤ 2.88 × 10^−05^), pod index and TcSNP 555 (*P* ≤ 8.24 × 10^−04^), and seed number and TcSNP 1160 (*P* ≤ 1.35 × 10^−04^) as well as those confirmed with FarmCPU (Table 3), suggest that chromosome 4 may contain a cluster or ‘hotspot’ of QTLs for yield-related traits. Several MTAs involving TcSNPs 344 and 555 on chromosome 4, TcSNP 733 on chromosome 5 and TcSNP 642 on chromosome 8 were also confirmed with FarmCPU.

There were two putative candidate genes involved with seed development co-localised with TcSNP 344 on chromosome 4, six that encode for seed development that were co-localised with TcSNP 667 (*TASSEL* MLM), eight responsible for seed protein and development and sugar transport that were linked to TcSNP 555, including Sugar transporter ERD6-like 16, and five with seed development functions co-localised with TcSNP 1160 (*TASSEL* MLM) (Table 4). In addition, seven genes involved with lipid formation and seed development, such as Acyltransferase-like protein At3g26840%2C chloroplastic, Glycerol-3-phosphate dehydrogenase, and Stearoyl-[acyl-carrier-protein] 9-desaturase, were localised close to TcSNP 953 on chromosome 4. TcSNP 953 (position 2,822,152 on chromosome 4) is located 3.7 Kb upstream of the gene that encodes acyl transferase-like protein At3g26840. Putative E3 ubiquitin protein ligase DRIP2, which acts as a negative regulator of the response to water stress was also co-localised with SNP 555 on chromosome 4 (Table 4).

On chromosome 5, seven putative candidate genes were co-localised with TcSNP 733. These were all involved in seed development. Of particular interest was Glycerol-3-phosphate 2-O-acyltransferase 6, which is rate-limiting enzyme in the *de novo* pathway of glycerolipid synthesis (Table 4). One candidate gene encodes soluble inorganic pyrophosphatase 4, which is important for development, but also in stress resistance (including to cadmium ion response) in plants (https://www.uniprot.org/uniprot/Q9LFF9). The latter was not of functional significance in this study, but is important for optimised cocoa production systems. In addition, one functional candidate gene involved with seed development was co-localised with TcSNP 823 on chromosome 5, based on *TASSEL* MLM (Table 4).

Three putative candidate genes were found on chromosome 7, one of which was a sugar transporter. Likewise, three functional candidate genes with seed development roles were detected on chromosome 8 although the MTA, involving pod index and SNP 642, was below the stringent Bonferroni threshold (*P* ≤ 4.39 × 10^−04^) based on *TASSEL* MLM. The latter MTA was verified and significant at *P* ≤ 1.02 × 10^−3^ based on the FarmCPU analysis (Table 3).

*TASSEL* MLM revealed a MTA of functional significance on chromosome 9, albeit below the Bonferroni threshold. TcSNP 184, located at 10,934,938 bp on chromosome 9, was co-localized with four putative candidate genes, one of which encodes Zinc finger protein CONSTANS-LIKE 5. The latter is involved in the regulation of flower development and regulation of transcription. The most significant MTA (*P* ≤ 9.57 × 10^−05^) on chromosome 9, based on the FarmCPU analysis, involved TcSNP932, at position 1,479,475 bp, and log cotyledon mass (Table 3). It was 14.3 Kb downstream of 1-acyl-sn-glycerol-3-phosphate acyltransferase 1%2C chloroplastic, which has biological significance in this study.

It must also be noted that TcSNP 401 (20,485,872), located 199 Kb upstream of *Tc00_t058610*, which encodes a putative MYB-related protein 308, 2 Mb upstream of the gene, *Tc04v2_g008890*, which encodes a putative MYB family transcription factor, and 1.3 Mb upstream of the gene, *Tc04v2_g009300*, which encodes a MYB domain protein 20, was significantly associated with fruit surface (ridge) anthocyanin intensity on chromosome 4, based on the *TASSEL* and FarmCPU analyses. A significant association (*P* ≤ 10 × 4.57^−05^) (*TASSEL* MLM) was also found between fruit surface anthocyanin intensity and SNP 644 on chromosome 9 (S8 Table http://dx.doi.org/10.13140/RG.2.2.34215.21927).

With regard to floral traits, there was a highly significant (*P* ≤ 3.81 × 10^−05^) MTA, which was detected between sepal length and TcSNP 1334 on chromosome 3 when *TASSEL* MLM was performed (S8 Table http://dx.doi.org/10.13140/RG.2.2.34215.21927). This was not tested with GAPIT FarmCPU.

Of the 17 significant MTAs identified with *TASSEL* MLM, there were two for fruit shape oblate (an uncommon phenotype in this diverse cacao germplasm sample), and orbicular on chromosome 3. These MTAs involved TcSNPs 1353 and 1477. Interestingly, loci controlling fruit shape were dispersed over several chromosomes, representing independent linkage groups.

In summary, about 40 putative candidate genes of functional importance were identified in this study. These included those that encode protein precursors, carbohydrate transport, lipid synthesis/bioassembly, binding and metabolism, lipid transfer and seed storage, endosperm development and regulation of seed growth, embryo development leading to seed dormancy, seed development/morphogenesis and regulation of flower and pollen development and ovule fertilization as well as responses to water deprivation, cadmium contamination and other abiotic stresses and biotic (disease) stresses (Table 4).

### Genomic prediction value of traits

The genomic estimated breeding values (GEBV) of the phenotypic traits studied are presented in S9 Table (http://dx.doi.org/10.13140/RG.2.2.12404.83842). Of the qualitative traits studied, fruit basal constriction and filament anthocyanin intensity had predictive values greater than 0.5. The quantitative traits, seed number, seed mass, seed length, seed width, seed length to width ratio, pod index, fruit wall hardness, ovule number and fruit width had GEBV values greater than 0.5. The yield-related traits, seed number and dried seed (cotyledon) mass had GEBV values of 0.611 and 0.6014, respectively. The GEBV value of cotyledon length and width, indicators of seed size, were 0.6199 and 0.5435, respectively. Interestingly, ovule number had the largest GEBV value of 0.6325. It is regarded as a reliable predictor of seed number, which is dependent on successful pollination.

## Discussion

### Linkage disequilibrium patterns

Our findings, based on the linkage disequilibrium analyses, suggest that MTAs could be localized in the cacao genome with relatively high precision particularly when wild cacao genotypes are studied. This concurs with the observations of Stack et al. [40], who observed that the wild genotypes (such as those from the Purús, Contamana, Curaray, Iquitos, Nanay, Marañón and Guiana genetic groups) exhibited very low overall LD. In our study, an average decay of *r*^2^ to 50% over chromosomes 1, 4, 5, 7 and 9 occurred at a relatively short distance of 5.21 Mb (9 cM) on average (S7 Table http://dx.doi.org/10.13140/RG.2.2.20793.44647). It is instructive that for cultivated germplasm such as Trinitario (*e*.*g*., the Imperial College Selection (ICS) accession group (S1 Table http://dx.doi.org/10.13140/RG.2.2.16179.71202), LD decay was observed in previous studies to be very gradual with increasing marker distance [40, 63].

### Marker-trait associations (MTAs)

The highly significant MTAs involving quantitative yield-related traits, observed in this study, suggest stability of genomic regions involved. However, the genetic markers, co-localized with genes, were not major (Table 3). Previous studies [10, 33, 63–72] identified useful MTAs and reference has been made to 300 QTLs [33]. Knowledge gleaned on MTAs related to yield potential (as measured by Pod Index), dried individual seed mass, seed size and seed number per fruit in diverse cacao germplasm should prove valuable for future genomics-assisted breeding for yield improvement.

The relatively small effect size of the markers associated with yield-related traits, in this study, where none of the markers explained more than 20% of the phenotypic variation expressed, is not unusual for quantitative traits. Most of the markers studied explained 5 to 11% of the phenotypic variation expressed.

Genetic variation of quantitative (polygenic, continuous) traits such as yield and disease resistance are controlled by the combined effects of QTL, epistasis (interactions between QTLs) [14], the environment and interaction between environment and QTL [73]. The use of only biallelic subsets of SNPs, in this study, could have excluded multiallelic loci, which may have contributed to additional variance expressed in the study population for polygenic traits, such as those related to yield potential. Mir et al. [74] described yield as a very complex quantitative trait that is controlled by a network of minor genes. For such polygenic traits, with small effect size, increasing the sample size of the study population and densely sampling a population that shows phenotypic diversity should improve the power to detect meaningful associations [75].

A phenomenon whereby significant associations were found in this study between certain traits, such as fruit anthocyanin intensity, fruit shape and seed length to width ratio and seed number, at different loci (Table 3) was of interest. It suggests that a large part of trait variance was explained by several marker-trait associations, as has been observed in other studies [73]. In this study, there were 26 SNPs significantly associated with fruit shape, two on chromosome 1, seven on chromosome 2, nine on chromosome 3, three on chromosome 5, two on chromosome 7 and three on chromosome 8, based on the results of *TASSEL* MLM. It seems justifiable to hypothesize that minor genes as well as major genes affect fruit shape. The oblate shape is a trait associated with certain wild types, which have evolved over a long period of time. A well-known cacao accession with this trait is CATONGO.

The presence of markers significantly associated with different traits, in the same genomic region, was also observed in this study, based on the *TASSEL* MLM. The latter traits were seed number and orbicular shape on chromosome 7 (SNP 390) and seed number and seed length, also on chromosome 7 (SNP 1335) (Tables 3 and 4). These associations may represent the occurrence of linked genes, each one coded separately for a specific trait [11] or may indicate co-localization of the respective markers with a gene or gene block with putative pleiotropic effect [76]. In the case of seed number and seed length, this apparent linkage is striking since pod index (a measure of yield potential) is derived from seed number and dried individual cotyledon mass. The latter was found to be significantly correlated with seed dimensions (length and width) in this study (Fig 2). The relevant associated putative genes may have adaptive influence due to linkage mediated by selective forces [77]. The likelihood of such a phenomenon being observed during this study was feasible due to the inclusion of at least 48 cultivated accessions, including 28 Imperial College Selections (ICS) (S1 Table http://dx.doi.org/10.13140/RG.2.2.16179.71202). The latter reportedly evolved over a period of more than two hundred years in Trinidad and Tobago and were selected based on large seed size and seed number and favourable yield, among other selection criteria. It must be underscored that pleiotropic markers may facilitate simultaneous selection of the multiple traits with which they are significantly associated and thus gene pyramiding.

### Putative annotated candidate genes for yield-related traits

The search for candidate genes in this study was guided by the fact that the storage compounds of cacao seeds are starch, lipids (fats) and storage proteins [78]. Bucheli et al. [79] investigated the variation of sugars, carboxylic acids, purine alkaloids, fatty acids, and endoproteinase activity during maturation of cacao seeds. Aspartic endoproteinase activity was observed to increase rapidly during seed expansion and a major change in the fatty acid composition occurred in the young embryo. Mustiga et al. [80] detected a major QTL explaining 24% of the relative level of palmitic acid on the distal end of chromosome 4, located close to the *Thecc1EG017405* gene. The latter is an orthologue and isoform of the stearoyl-acyl carrier protein (ACP) desaturase (SAD) gene that is involved in fatty acid biosynthesis. Cacao seeds also contain a vicilin-like globulin, a seed storage protein [81].

There are three acyltransferases and a phosphohydrolase involved in the bioassembly of plant storage lipids, *viz*., glycerol-3-phosphate acyltransferase (GPAT), lyso-phosphatidic acid acyltransferase (LPAT), diacylglycerol acyltransferase (DGAT) and phosphatidate phosphohydrolase (PAPase) [82]. Fritz et al. [83] purified glycerol-3-phosphate acyltransferase from the post-microsomal supernatant of cocoa seeds.

Triacylglycerols (TAGs) are the major storage lipids in several plants and serve as energy reserves in seeds that are later used for germination and seedling development [84, 85]. The terminal step in TAG formation in plants involves the catalytic action of diacylglycerol acyltransferase (DGAT) in the presence of acyl-CoA [84]. Developing seeds in *Brassica napus* have been reported to produce Diaylglycerol (DAG) during the active phase of oil accumulation [86].

The proteins encoded by candidate genes, which were co-localized with SNPs found to be significantly associated with yield-related traits, during this study (Table 3), are presented in Table 4. An association (just below the stringent significance level with Bonferroni correction) observed between seed length (*TASSEL* MLM and FarmCPU) and seed cotyledon mass (FarmCPU) and TcSNP 953 on chromosome 4, at a position of 2,822,152 bp, 3.7 Kb upstream of a gene that encodes diacylglycerol acyltransferase (Acyltransferase-like protein At3g26840%2C chloroplastic) (Table 4), is among the most noteworthy of this study and warrants further investigation with expression studies.

At3g26840 is involved in seed storage (globulins) and seed size in Assembly cotton 46 (*Gossypium* spp.) [82]. *Gossypium* spp. are related to cacao, both being members of the Malvaceae family. The discovery of genes that encode Glycerol-3-phosphate dehydrogenase, and Stearoyl-[acyl-carrier-protein] 9-desaturase on chromosome 4 was also of relevance. The association revealed by FarmCPU between TcSNP 932, on chromosome 9, with log cotyledon mass, must be considered due to its functional importance. This SNP was co-localised with 1-acyl-sn-glycerol-3-phosphate acyltransferase 1%2C chloroplastic, which is involved in lipid metabolism.

Another putative candidate gene, unravelled during this study, was Tc01v2_g022850, which encodes Bifunctional inhibitor/lipid-transfer protein/seed storage 2S albumin superfamily protein involved in Glycerolipid metabolism (chromosome 1, 2.5 Mb downstream of SNP 785 (31,785,158) on chromosome 1) (Table 4). It is must also be recorded that TcSNP 555, on chromosome 4, was co-localised with vicilin in this study.

### Consensus MTAs involving yield-related traits in cacao

Since several hundred MTAs or QTLs have previously been identified in cacao, an attempt was made to check for congruence between the findings of this study and previous ones with respect to common traits. Highly significant (stable) associations between yield-related traits, seed length and seed length to width ratio, seed number and seed (cotyledon) mass and pod index, and SNPs were found on chromosomes 1, 3, 4, 5, 7 and 9 in this study (Table 3). The findings from previous studies that coincide with those of this study are highlighted below.

Eight significant associations between SSR markers and yield-related traits were reported by Marcano et al. [87]. These included associations with fresh seed mass on chromosomes **1**, 2, **5**, 6, **9** and 10, MTAs involving dried seed mass (100 seeds) on chromosomes 2, **4**, **9** and 10 as well as one marker associated with seed number per fruit on chromosome **5**. In their mapping study, dos Santos Fernandes et al. [88] identified several QTLs flanked by the markers on chromosome **4**, which were associated with pod index, dried individual seed mass, number of fruits harvested and number of healthy fruits harvested. They also identified a significant association between the marker, *Tcm002s23708704*, and pod index on chromosome 2. dos Santos Fernandes et al. [88] unravelled 13 candidate genes linked to yield (dried seed mass, pod number), on chromosomes **4** and 2. Nine of these genes are annotated as transmembrane transporters, specializing in sugar transport, two genes are involved in carbohydrate metabolism, one gene codes for lipid metabolism, and one is involved in glucose metabolism [88]. Motilal et al. [64] identified three SNPs (TcSNP 368, 697, 1370) on chromosomes **1** and **9** that were significantly associated with seed number. Clément et al. [20] found two QTLs for yield in the clone, POUND 12, located close to a QTL for yield, identified in IMC 78, on chromosome **4**.

**Fig 7:**
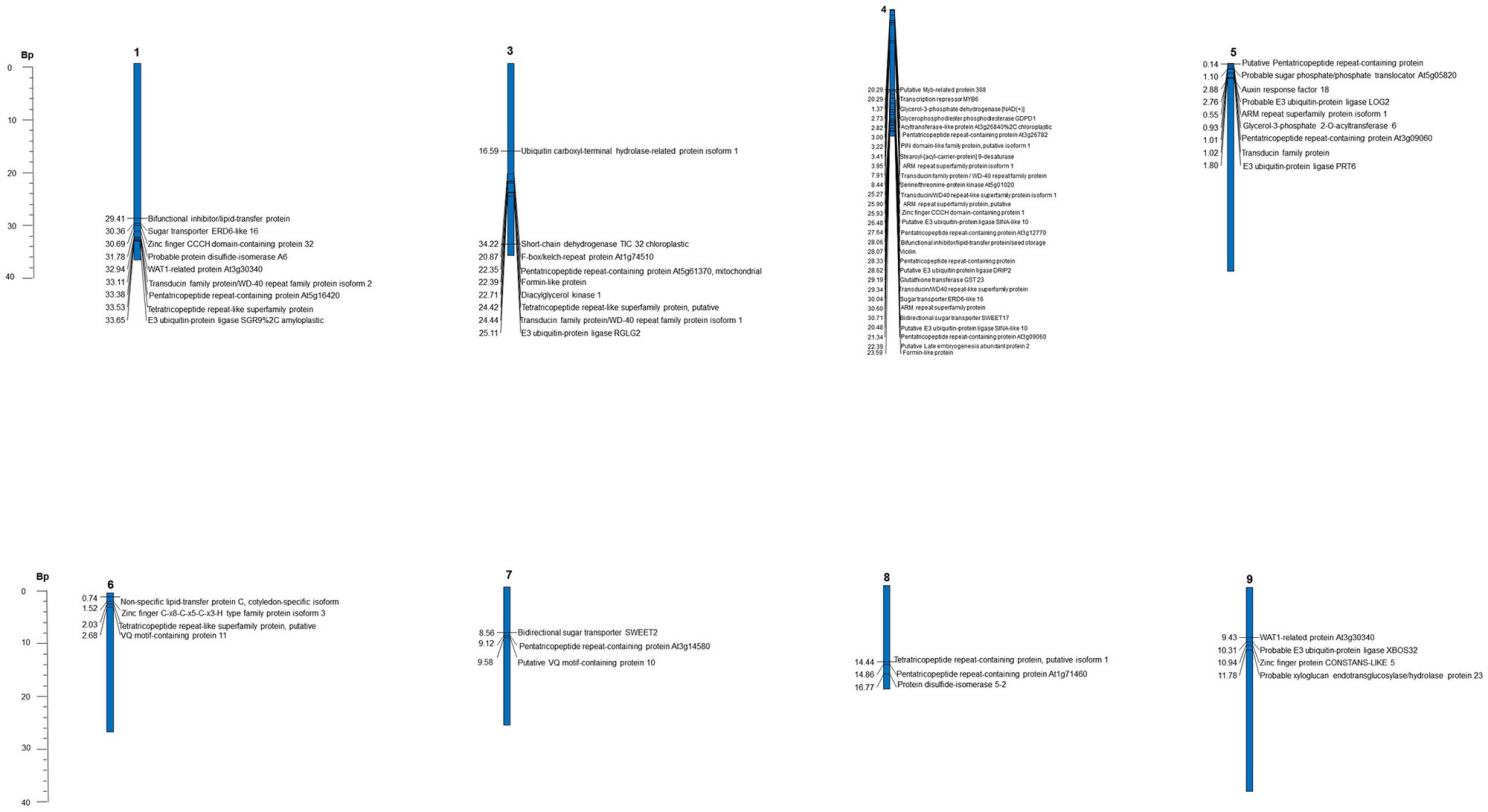
Physical map of *T*. *cacao* L. showing annotated candidate genes, which were co-localised with SNP markers associated with yield-related and other traits. Gene loci and proteins are shown on the right and genetic distances (Mb) are shown on the left. No candidate genes were identified on chromosomes 2 and 10.

Previous studies thus commonly observed loci on chromosomes 1, 2, 4, and 9 to be associated with seed mass and dimensions in cacao [87]. The findings of this study suggest that yield-related traits are associated with loci on chromosomes 1, 3, 4, 5, 7, 8 and 9, putatively linked to functional genes (Tables 3 and 4). Two SNPs, TcSNP 344 and 953, were associated with seed length on chromosome **4** (Tables 3 and 4). TcSNP 953 is located at the top of chromosome 4 (Fig 7) and thus the MTA, though below the stringent Bonferroni level of significance, may be considered validated since it was observed in a common region where yield-related MTAs were located in the studies by dos Santos Fernandes et al. [88] and Clément et al. [20].

A highly significant MTA, observed in this study, involved seed number and TcSNP 785 on chromosome **1** (*TASSEL* MLM). Motilal et al. [64] also reported such an MTA on chromosome **1**.

Criollo, Forastero and Trinitario are widely recognised classes of cocoa in the trade. The cacao accessions in this study that displayed favourable yield potential were mainly Trinitario (cultivated germplasm) [44] and those with Criollo ancestry. This was due mainly to their large seed sizes (Table 5). This supports the deduction of Doebley et al. [89] that “cultivated species generally have larger fruits or seeds compared to their wild ancestors, indicating that fruit and seed size are major agronomic traits that have been selected in crops during their domestication.” However, several Upper Amazon Forastero types, in this study, also had favourable (low) pod index due to their large seed numbers (Table 5, S1 Fig http://dx.doi.org/10.13140/RG.2.2.24148.88966).

**Table 5:** Superior accessions in terms of yield-related traits and associated genotypes (allelic variants) for SNP markers of interest.

Accession	Ovule number	Total fresh seed mass (g)	Seed number	Cotyledon mass (g)	Cotyledon length (cm)	Cotyledon width (cm)	Cotyledon Length: width	Pod Index (≤21)	TcSNP 642 allelic variant	TcSNP 555 allelic variant	TcSNP953 allelic variant
UF11	39	101	39	1.84	2.72	1.51	1.8	13.94	A:C	A:A	A:A
ICS60	37	94	39	1.64	2.63	1.49	1.77	15.63	A:C	A:A	A:A
LCTEEN-261/S_4	43	94.5	43	1.41	2.58	1.33	1.94	16.49	A:C	A:A	A:A
IMC10	54	82.2	58	1.02	2.5	1.2	2.08	16.9	A:C	A:A	A:T
ICS62	58	79.5	54	1.09	2.24	1.25	1.79	16.99	C:C	A:A	A:A
UF676	37	85.8	39	1.49	2.57	1.48	1.74	17.21	A:C	A:A	A:A
ICS6	43	86.5	43	1.33	2.45	1.47	1.67	17.49	A:A	A:A	A:A
ICS75	40	74.3	38	1.41	2.44	1.41	1.73	18.66	A:C	A:A	A:A
IMC3	62	74.2	45	1.19	2.68	1.31	2.05	18.67	A:C	A:A	A:A
IMC33	49	74.1	52	1.02	2.39	1.25	1.91	18.85	A:C	A:A	unknown
IMC65	53	85.2	50	1.06	2.35	1.17	2.01	18.87	A:A	A:A	A:T
MATINA1-7	46	78.5	36	1.46	2.65	1.47	1.8	19.03	C:C	A:A	A:A
IMC71	60	90.8	56	0.92	2.14	1.12	1.91	19.41	A:C	A:A	A:A
ICS63	39	80.7	39	1.31	2.57	1.42	1.81	19.57	A:C	A:A	A:A
SC19	37	77	33	1.54	2.46	1.46	1.68	19.68	A:C	A:A	A:A
IMC27	52	88.6	54	0.94	2.35	1.07	2.2	19.7	A:C	A:A	A:A
ICS8	39	70.1	40	1.26	2.35	1.39	1.69	19.84	A:C	A:A	A:A
ICS1	39	78.3	39	1.29	2.58	1.38	1.87	19.88	A:A	A:A	A:A
IMC61	54	71.4	57	0.88	2.31	1.15	2.01	19.94	A:C	A:A	A:T
IMC68	51	75.8	51	0.98	2.22	1.16	1.91	20.01	A:C	A:C	unknown
IMC67	48	79.2	48	1.04	2.27	1.07	2.12	20.03	A:C	A:C	A:A
ICS48	40	72	36	1.38	2.52	1.43	1.76	20.13	A:C	A:A	unknown
SCA9	57	65.9	46	1.07	2.34	1.3	1.8	20.32	A:C	A:C	A:A
IMC77	48	76.5	54	0.91	2.4	1.15	2.09	20.35	A:C	A:A	unknown
ICS15	42	74.1	34	1.44	2.44	1.56	1.56	20.42	A:C	A:A	unknown
IMC39	55	68.4	59	0.82	2.32	1.22	1.9	20.67	A:C	A;A	unknown
IMC63	44	71	52	0.93	2.49	1.14	2.18	20.68	A:C	A:A	A:T
LCTEEN20S10	43	67.9	38	1.27	2.32	1.34	1.73	20.72	A:C	A:A	A:A
ICS40	50	71.1	49	0.98	2.36	1.13	2.09	20.82	A:C	A:A	A:A

82.8% of the elite cacao accessions had genotype A:C for TcSNP 642.

89.7% of these cacao accessions had genotype A:A for TcSNP 555.

61% had genotype A:A for TcSNP 953 (data were unavailable for seven of the accessions).

### Improving yield potential with MTAs unravelled

Based on the findings of this study, elucidation and selection of genotypes associated with large seed size in *T*. *cacao* may thus be facilitated by using TcSNP 953, TcSNP 555 and TcSNP 344 on chromosome 4 and TcSNP 733 on chromosome 5 (Tables 3 and 4). The results of a preliminary evaluation to detect genotypes associated with favourable yield potential (pod index) are presented in Table 5 and involve TcSNP 555, TcSNP 642 (on chromosome 8) and TcSNP 953. Further investigation with haplotype inferencing for genomic prediction and involving training and test populations of *T*. *cacao* L. in genomic selection studies, as described by Bhat et al. [90], is recommended.

Additional studies to investigate functional genomics associated with yield-related traits in cacao are also recommended. Such studies, in wheat, have revealed transcription factors, which can affect seed number, genes involved in metabolism or signalling of growth regulators, genes determining cell division and proliferation related to seed size, and floral regulators that regulate inflorescence architecture and seed number. Genes involved in carbohydrate metabolism, affecting plant architecture and grain yield such as trehalose phosphate synthase (TPS) and trehalose phosphate phosphatase (TPP) genes have also been identified [91].

These recommended follow-up studies would entail gene expression analysis, involving transcriptomics as described by Jako et al. [82]. The DGAT gene, Tag1, from *Arabidopsis* was shown to encode an acyl-CoA-dependent DGAT. Jako et al. [82] also demonstrated that seed-specific over-expression of the DGAT cDNA in wild-type *Arabidopsis* “enhances oil deposition and average seed mass, which are correlated with DGAT transcript levels”, and that “DGAT has an important role in regulating the quantity of seed TAGs, the sink size in developing seeds and thus seed size.” They also demonstrated that “over-expression” of the acyl-CoA-dependent DGAT in a “seed-specific manner in wild-type *Arabidopsis* plants results in increased oil deposition and average seed mass.”

Seed size has also been shown to be directly determined by carbohydrate import into seeds, in maize and rice, and involves SWEET-mediated hexose transport [92]. SWEET genes regulate the transport, distribution and storage of carbohydrates in plants, and are involved in many important physiological processes, including phloem loading, reproductive development, disease-resistance, stress response, and host-pathogen interaction. In this study, SWEET17 was localized on chromosome **4** upstream of TcSNP555 (Table 4), which was associated with pod index (*P* ≤ 4.29 × 10^−4^). SWEET 2 was also localized downstream of TcSNP1335, which was significantly associated (*P*≤ 1.15 ×10^−14^) with seed number as well as with log seed length (*P*≤ 6.75 × 10^−05^) on chromosome 7 (*TASSEL* MLM) (Table 4).

### Anthocyanin pigmentation relationships

In this study, fruit ridge anthocyanin concentration (fruit colour) was significantly associated with TcSNP 401 on chromosome 4, located at 20,485,872, about 199 Kb upstream of the gene Tc00_t058610 (mRNA). The latter encodes a Putative MYB-related protein 308. TcSNP 401 was also 198 Kb upstream of the Transcription repressor MYB 6 (Table 4) (https://cocoa-genome-hub.southgreen.fr/). This SNP accounted for 7.1% of the phenotypic variation in fruit colour observed (*P* ≤ 4.78 × 10^−06^) with *TASSEL* MLM (Table 3). The MTA was corroborated with FarmCPU. Four SNP variants on chromosome 4, co-localised with a MYB transcription factor gene (TcMYB113), were inferred to encode ‘fruit colour differences’ between cacao varieties by Motamayor et al. [70].

Another significant association (*P* ≤ 6.79 × 10^−05^) for fruit surface anthocyanin intensity was found in this study with *TASSEL* MLM analysis. It involved TcSNP 1203, on chromosome 3 (located at 563,101 bp), which accounted for 5.6% of the phenotypic variation in fruit surface anthocyanin concentration in this cohort of germplasm.

Three regions associated with pigmentation on different organs in cacao have been identified by Marcano et al. [87]. This sector includes the major locus identified by Crouzillat et al. [12] as responsible for controlling ‘seed colour’ in Catongo x POUND 12 backcross progeny.

MYB proteins are involved in regulatory networks controlling metabolism, including the synthesis of anthocyanins, responsible for the red pigmentation in cacao [93, 94]. Liu et al. [95] observed that overexpression of the Tc-MYBPA gene elicited increased expression of several genes encoding the major structural enzymes of the proanthocyanidin and anthocyanidin pathway in cacao.

There were also significant associations between filament anthocyanin concentration and SNPs on chromosomes 3 and 8 (TcSNPs 1183 and 1441, respectively), based on the *TASSEL* MLM results of this study (Table 4, S8 Table http://dx.doi.org/10.13140/RG.2.2.34215.21927). All of the MTAs involving anthocyanin concentration in this study suggest multi-gene control of anthocyanin intensity in the mature fruit epidermis and flower filaments of cacao. Furthermore, the differential expression of pigmentation in the seeds and fruits of cacao, observed at the ICGT, as evidenced in the negative correlations in Fig 2, and as stated by Bartley [96], may be explained by the association of pigmentation of seeds and fruit pigmentation with several genomic regions.

MTAs involving anthocyanin intensity warrant further investigation to determine their value for genomic selection due to the significance of this trait in differentiating among certain genotypes of interest, such as CCN-51 and ancient Nacional cacao [70, 94], the nutraceutical value of anthocyanin and its putative role in cacao disease resistance [34, 95].

## Highlights of this study

The results of this study support the observation of Rockman [97] that most complex traits (such as those related to yield in cacao) are controlled by several (putatively interacting) loci with small effects. While some phenotypic traits are controlled by a small number of loci with large effects (as is often the case for traits under biotic selection) [74], others, such as yield, may have more complex genetic architectures. The latter may be controlled by many rare variants, each having a large effect on the phenotype or conversely, many common variants with only small effects on the phenotypes [98]. The causative variants may be clustered in one or a small number of genes or across many genes.

The data on gene ontology presented in Table 4 provide evidence of polygenic control of yield-related traits in cacao. For such traits, it may be more effective to predict the performance of genotypes by using multiple molecular markers [98]. Multilocus mixed linear models (MMLMs) may thus be considered for future studies in cacao, when complex traits are investigated, because these incorporate multiple markers simultaneously as covariates in a stepwise MLM [99].

The observations regarding drought tolerance in this study are of considerable significance since the conditions at the ICGT, where these accessions were conserved, are considered sub-optimal in terms of soil moisture content [44]. Therefore, the latter results, which indicate drought adaptation among genotypes with inherent high yield potential, merit follow-up functional genomic studies.

Once the functional roles of putative genes co-localized with markers with significant associations to traits of interest are elucidated, the effects of relationships of these putative genes with geography and local adaptation must be established, as recommended by McKown et al. [100]. Micheli et al. [101] have reported on functional genomics in cacao focusing on genes expressed under specific physiological conditions. Consistency in QTL effects over different genetic backgrounds must also be established. Individuals identified with favourable marker genotypes and haplotypes may then be used as parental types for enhancement or breeding programmes, such as those involving Multiparent Advanced Generation Intercross Populations (MAGIC), in targeted environments.

Despite the fact that yield is a complex trait, our results on potential genomic selection (GS) for yield traits are very promising, given the high predictive values obtained for these traits, generally superior to 0.5. The detection of several markers associated with yield-related traits with good predictive value, in this study, could facilitate GS and marker-assisted selection (MAS) for yield in cacao.

## Conclusion

In this study, carefully collated phenotyping data on traits of economic interest in cacao, such as yield potential (S2 Table http://dx.doi.org/10.13140/RG.2.2.29601.48484), and SNP genotyping data (S10 Table http://dx.doi.org/10.13140/RG.2.2.25826.61123), generated via transcriptome sequencing, were subjected to GWAS. Twenty-nine cacao accessions represent promising material for breeding in terms of yield potential. The results presented herein indicated oligogenic and polygenic control of yield-related traits in cacao. The stringently significant marker-trait associations related to yield, found in this study, were indications of the presence of QTL on chromosomes such as 3, 4, 5, 7, 8 and 9. Chromosome 4 putatively contains a QTL cluster associated with yield-related traits. Some annotated candidate genes associated with seed development, seed lipid accumulation, metabolism and development and plant stress responses (including to drought and to cadmium) have been identified during this research. The identification of yield-related traits with good predictive value could further facilitate GS for yield potential in cacao. The performance of the non-phenotyped individuals at the Trinidad genebank (ICGT) could also be predicted once they are genotyped. This would be particularly useful for the enhanced genotypes (GEBP progeny) [45]. Identification of parents possessing high predictive values and favourable alleles and haplotypes, prior to crossing, should prove beneficial for more rapid development of enhanced cacao progenies.

## Supporting information

S1 Table Background information on the *T*. *cacao* L. accessions used in the analyses. http://dx.doi.org/10.13140/RG.2.2.16179.71202.
(DOCX)

S2 Table Phenotypic data for 346 cacao accessions fully phenotyped and used to generate descriptive statistics. http://dx.doi.org/10.13140/RG.2.2.29601.48484.
(XLSX)

S3 Table Results of tests of normality performed on the natural log transformed fruit and seed quantitative traits. http://dx.doi.org/10.13140/RG.2.2.22890.59849.
(DOCX)

S4 Table Descriptive statistics for quantitative fruit and seed traits in wild, cultivated and unclassified cacao germplasm studied. http://dx.doi.org/10.13140/RG.2.2.36312.37128.
(DOCX)

S5 Table ANOVA for yield-related traits. http://dx.doi.org/10.13140/RG.2.2.17438.00328.
(DOCX)

S6 Table Coefficients of membership for clusters of accessions based on STRUCTURE analysis. http://dx.doi.org/10.13140/RG.2.2.27504.33282.
(DOCX)

S7 Table Distances over which linkage disequilibrium decayed to 50 percent over chromosomes 1, 4, 5, 7 and 9. http://dx.doi.org/10.13140/RG.2.2.20793.44647.
(DOCX)

S8 Table Summary of significantly positive marker-trait associations (*TASSEL* MLM and GAPIT FarmCPU). http://dx.doi.org/10.13140/RG.2.2.34215.21927.
(XLSX)

S9 Table Predictive values (GEBV) of phenotypic traits associated with SNPS. http://dx.doi.org/10.13140/RG.2.2.12404.83842.
(DOCX)

S10 Table Genotype data used in GWAS. http://dx.doi.org/10.13140/RG.2.2.25826.61123.
(TXT)

S1 Fig Summary report for Pod index in wild cacao and Summary report for Pod index in cultivated cacao. http://dx.doi.org/10.13140/RG.2.2.24148.88966.
(TIF)

## References

[1] Alverson WS, Whitlock BA, Nyffeler R, Bayer C, Baum DA. Phylogeny of the core Malvales: evidence from ndhF sequence data. American Journal of Botany. 1999 Oct;86(10):1474–86. doi: 10.2307/2656928 10523287

[2] Expert market research. Expert Market Research Report. 2020. https://www.expertmarketresearch.com/reports/chocolate-market. Accessed August 6, 2020.

[3] Argout X, Salse J, Aury JM, Guiltinan MJ, Droc G, Gouzy J et al. The genome of *Theobroma cacao*. Nature Genetics. 2011 Feb;43(2):101–8. doi: 10.1038/ng.736 21186351

[4] Cheesman EE. Notes on the nomenclature, classification and possible relationships of cacao populations. Tropical Agriculture. 1944;21(8).

[5] Eskes A, Lanaud C. Cocoa. In: Charrier A, Jacquot M, Hamon S, Nicolas D, editors. Tropical Plant Breeding. Montpellier: CIRAD; 2001. p. 78–105.

[6] Bekele F, Phillips-Mora W. Cocoa Breeding. In: Al-Khayri JM et al., editors. Advances in Plant Breeding: Industrial and Food Crops, Vol 6. Springer-Verlag, Cham; 2019. p. 409–87. doi: 10.1007/978-3-030-23265-8_12

[7] Lanaud C, Fouet O, Legavre T, Lopes U, Sounigo O, Eyango MC et al. Deciphering the *Theobroma cacao* self-incompatibility system: from genomics to diagnostic markers for self-compatibility. Journal of Experimental Botany. 2017 Oct 13;68(17):4775–90. doi: 10.1093/jxb/erx293 29048566 PMC5853246

[8] Ribeyre F, Sounigo O, Argout X, Cilas C, Efombagn MI, Denis M et al. The genomic selection of *Theobroma cacao* L: a new strategy of marker assisted selection to improve breeding efficiency and predict useful traits in new populations. International Symposium on Cocoa Research. 2017 Nov 13–17; Lima, Peru. London: ICCO. http://agritrop.cirad.fr/589763/1/ID589763.pdf

[9] Simmonds NW. The breeding of perennial crops. Proceedings of the Workshop on the Conservation, Characterisation and Utilization of Cocoa Genetic Resources in the 21st Century;1992 Sep 13–17; Port-of-Spain, Trinidad. Port-of-Spain: The Cocoa Research Unit;1993. p. 156–62.

[10] Clément D, Risterucci AM, Motamayor JC, N’Goran J, Lanaud C. Mapping QTL for yield components, vigor, and resistance to *Phytophthora palmivora* in *Theobroma cacao* L. Genome. 2003a Apr 1;46(2):204–12. doi: 10.1139/g02-125 12723036

[11] Araújo IS, de Souza Filho GA, Pereira MG, Faleiro FG, de Queiroz VT, Guimarães CT et al. Mapping of quantitative trait loci for butter content and hardness in cocoa beans (*Theobroma cacao* L.). Plant Molecular Biology Reporter. 2009 Jun 1;27(2):177–83. https://link.springer.com/content/pdf/10.1007/s11105-008-0069-9.pdf

[12] Crouzillat D, Lerceteau E, Pétiard V, Morera J, Rodríguez H, Walker D et al. *Theobroma cacao* L.: a genetic linkage map and quantitative trait loci analysis. Theoretical and Applied Genetics. 1996 Jul 1;93(1–2):205–14. doi: 10.1007/BF00225747 24162219

[13] Crouzillat D, Ménard B, Mora A, Phillips W, Pétiard V. Quantitative trait analysis in *Theobroma cacao* using molecular markers. Euphytica. 2000a Jul;114(1):13–23. doi: 10.1023/A:1003892217582

[14] Crouzillat D, Phillips W, Fritz PJ, Pétiard V. Quantitative trait loci analysis in *Theobroma cacao* using molecular markers. Inheritance of polygenic resistance to *Phytophthora palmivora* in two related cacao populations. Euphytica. 2000b Jul;114(1):25–36. doi: 10.1023/A:1003994212394

[15] N’Goran JA, Risterucci AM, Clément D, Sounigo O, Lorieux M, Lanaud C. Identification of quantitative trait loci (QTL) in *Theobroma cacao* L. L. Agron Afr. 1997;9:55–63.

[16] Lanaud C, Kébé IS, Risterucci AM, Clément D, N’Goran JA, Grivet L et al. Mapping quantitative trait loci (QTL) for resistance to *Phytophthora palmivora* in *T*. *cacao*. Proceedings of the 12th International Cocoa Research Conference; 1996 Nov 17; Bahia, Brazil. Lagos: Cocoa Producers’ Alliance; 1999. p. 99–105.

[17] Lanaud C, Boult E, Clapperton J, N’Goran JKA, Cros E, Chapelin M et al. Identification of QTLs related to fat content, seed size an sensorial traits in *Theobroma cacao* L. Proceedings of the 14th International Cocoa Conference; 2003 Oct; Accra, Ghana. Lagos: Cocoa Producers’ Alliance;2005. p. 1119–26.

[18] Lanaud C, Fouet O, Clément D, Boccara M, Risterucci AM, Surujdeo-Maharaj S et al. A meta-QTL analysis of disease resistance traits of *Theobroma cacao* L. Molecular Breeding. 2009 Nov;24(4):361–74. doi: 10.1007/s11032-009-9297-4

[19] Flament MH, Kébé I, Clément D, Pieretti I, Risterucci AM, N’Goran JA et al. Genetic mapping of resistance factors to *Phytophthora palmivora* in cocoa. Genome. 2001 Feb 1;44(1):79–85. doi: 10.1139/g00-099 11269360

[20] Clément D, Risterucci AM, Motamayor JC, N’Goran J, Lanaud C. Mapping quantitative trait loci for bean traits and ovule number in *Theobroma cacao* L. Genome. 2003b Feb 1;46(1):103–11. doi: 10.1139/g02-118 12669802

[21] Clément D, Lanaud C, Sabau X, Fouet O, Le Cunff L, Ruiz E, et al. Creation of BAC genomic resources for cocoa (*Theobroma cacao* L.) for physical mapping of RGA containing BAC clones. Theoretical and Applied Genetics. 2004 May 1;108(8):1627–34. doi: 10.1007/s00122-004-1593-0 15235775

[22] Risterucci AM, Paulin D, Ducamp M, N’Goran JA, Lanaud C. Identification of QTLs related to cocoa resistance to three species of *Phytophthora*. Theoretical and Applied Genetics. 2003 Dec 1;108(1):168–74. doi: 10.1007/s00122-003-1408-8 13679987

[23] Pugh T. Etude du déséquilibre de liaison chez le cacaoyer appartenant aux groupes Criollo/Trinitario. Application au marquage génétique d’intérêt pour la sélection. Thèse Doctorat. Montpellier: Ecole National Supérieur d’Agonomie; 2005.107 p.

[24] Pugh T, Fouet O, Risterucci AM, Brottier P, Abouladze M, Delettrez C et al. A new codominant marker-based cocoa linkage map: development and integration of new microsatellite markers into cocoa linkage map. A new cocoa reference map. Proceedings of 14th International Cocoa Research Conference; 2003 Oct 13–18; Accra, Ghana. Lagos: Cocoa Producers’ Alliance; 2005. p. 153–60.

[25] Brown JS, Schnell RJ, Motamayor JC, Lopes U, Kuhn DN, Borrone JW. Resistance gene mapping for witches’ broom disease in *Theobroma cacao* L. in an F_2_ population using SSR markers and candidate genes. Journal of the American Society for Horticultural Science. 2005 May 1;130(3):366–73. doi: 10.21273/JASHS.130.3.366

[26] Brown JS, Phillips-Mora W, Power EJ, Krol C, Cervantes-Martinez C, Motamayor JC et al. Mapping QTLs for resistance to frosty pod and black pod diseases and horticultural traits in *Theobroma cacao* L. Crop Science. 2007 Sep;47(5):1851–8. doi: 10.2135/cropsci2006.11.0753

[27] Brown JS, Sautter RT, Tondo CT, Borrone J, Kuhn D, Motamayor J et al. A composite linkage map from the combination of three crosses made from commercial clones of cacao, *T*. *cacao* L. Trop Plant Biol. 2008 Apr 22;1(2):120–30. doi: 10.1007/s12042-008-9011-4

[28] Faleiro FG, Queiroz VT, Lopes UV, Guimarães CT, Pires JL, Yamada MM et al. Mapping QTLs for witches’ broom (*Crinipellis perniciosa*) resistance in cacao (*Theobroma cacao* L.). Euphytica. 2006 May;149(1):227–35. doi: 10.1007/s10681-005-9070-7

[29] Argout X, Martin G, Droc G, Fouet O, Labadie K, Rivals E et al. The cacao Criollo genome v2. 0: an improved version of the genome for genetic and functional genomic studies. BMC Genomics. 2017 Dec 1;18(1):730. doi: 10.1186/s12864-017-4120-9 28915793 PMC5603072

[30] Saski CA, Feltus FA, Staton ME, Blackmon BP, Ficklin SP, Kuhn DN et al. A genetically anchored physical framework for *Theobroma cacao* cv. Matina 1–6. BMC Genomics. 2011 Dec;12(1):413–25. doi: 10.1186/1471-2164-12-413 21846342 PMC3173454

[31] Feltus FA, Saski CA, Mockaitis K, Haiminen N, Parida L, Smith Z et al. Sequencing of a QTL-rich region of the *Theobroma cacao* genome using pooled BACs and the identification of trait specific candidate genes. BMC Genomics. 2011 Dec;12(1):1–6. doi: 10.1186/1471-2164-12-379 21794110 PMC3154204

[32] Fouet O, Allegre M, Argout X, Jeanneau M, Lemainque A, Pavek S et al. Structural characterization and mapping of functional EST-SSR markers in *Theobroma cacao*. Tree Genetics & Genomes. 2011 Aug;7(4):799–817. doi: 10.1007/s11295-011-0375-5

[33] Allegre M, Argout X, Boccara M, Fouet O, Roguet Y, Bérard AU et al. Discovery and mapping of a new expressed sequence tag-single nucleotide polymorphism and simple sequence repeat panel for large-scale genetic studies and breeding of *Theobroma cacao* L. DNA Research. 2012 Feb 1;19(1):23–35. doi: 10.1093/dnares/dsr039 22210604 PMC3276266

[34] Argout X, Fouet O, Wincker P, Gramacho K, Legavre T, Sabau X et al. Towards the understanding of the cocoa transcriptome: Production and analysis of an exhaustive dataset of ESTs of *Theobroma cacao* L. generated from various tissues and under various conditions. BMC Genomics. 2008 Dec 1;9(1):512. doi: 10.1186/1471-2164-9-512 18973681 PMC2642826

[35] Akhunov E, Nicolet C, Dvorak J. Single nucleotide polymorphism genotyping in polyploid wheat with the Illumina GoldenGate assay. Theoretical and Applied Genetics. 2009 Aug 1;119(3):507–17. https://link.springer.com/content/pdf/10.1007/s00122-009-1059-5.pdf 19449174 10.1007/s00122-009-1059-5 PMC2715469

[36] Myles S, Peiffer J, Brown PJ, Ersoz ES, Zhang Z, Costich DE et al. Association mapping: critical considerations shift from genotyping to experimental design. The Plant Cell. 2009 Aug 1;21(8):2194–202. http://www.plantcell.org/content/plantcell/21/8/2194.full.pdf doi: 10.1105/tpc.109.068437 19654263 PMC2751942

[37] Breseghello F, Sorrells ME. Association analysis as a strategy for improvement of quantitative traits in plants. Crop Science. 2006 May;46(3):1323–30. doi: 10.2135/cropsci2005.09-0305

[38] Li H, Bradbury P, Ersoz E, Buckler ES, Wang J. Joint QTL linkage mapping for multiple-cross mating design sharing one common parent. PloS One. 2011 Mar 15;6(3):e17573. doi: 10.1371/journal.pone.0017573 21423655 PMC3057965

[39] Hill WG, Robertson A. Linkage disequilibrium in finite populations. Theoretical and Applied Genetics. 1968 Jun;38(6):226–31. doi: 10.1007/BF01245622 24442307

[40] Stack JC, Royaert S, Gutiérrez O, Nagai C, Holanda IS, Schnell R et al. Assessing microsatellite linkage disequilibrium in wild, cultivated, and mapping populations of *Theobroma cacao* L. and its impact on association mapping. Tree Genetics & Genomes. 2015 Apr 1;11(2):19. doi: 10.1007/s11295-015-0839-0

[41] Bekele FL, Bekele I, Butler DR, Bidaisee GG. Patterns of morphological variation in a sample of cacao (*Theobroma cacao* L.) germplasm from the International Cocoa Genebank, Trinidad. Genetic Resources and Crop Evolution. 2006 Aug;53(5):933–48. doi: 10.1007/s10722-004-6692-x

[42] Motamayor JC, Lachenaud P, Da Silva e Mota JW, Loor R, Kuhn DN, Brown JS et al. Geographic and genetic population differentiation of the Amazonian chocolate tree (*Theobroma cacao* L). PloS One. 2008 Oct 1;3(10):e3311. doi: 10.1371/journal.pone.0003311 18827930 PMC2551746

[43] Bekele F, Butler DR. Proposed short list of cocoa descriptors for characterization. Working procedures for cocoa germplasm evaluation and selection. Proceedings of the CFC/ICCO/IPGRI Project Workshop; 1998 Feb 1–6; Montpellier, France. Rome: International Plant Genetic Resources Institute (IPGRI); 2000.p. 41–8.

[44] Bekele FL, Bidaisee GG, Singh H, Saravanakumar D. Morphological characterisation and evaluation of cacao (*Theobroma cacao* L.) in Trinidad to facilitate utilisation of Trinitario cacao globally. Genetic Resources and Crop Evolution. 2020a Mar;67(3):621–43. doi: 10.1007/s10722-019-00793-7

[45] Bekele F, Bidaisee G, Saravanakumar D. Examining phenotypic diversity and economic value of cacao (*Theobroma cacao* L.) conserved at the International Cocoa Genebank, Trinidad to support improvement in cocoa yield globally. Tropical Agriculture. 2020b (released 2021 Feb 25);97(2). https://journals.sta.uwi.edu/ojs/index.php/ta/article/view/7970

[46] Iwaro AD, Bekele FL, Butler DR. Evaluation and utilisation of cacao (*Theobroma cacao* L.) germplasm at the International Cocoa Genebank, Trinidad. Euphytica. 2003 Mar;130(2):207–21. doi: 10.1023/A:1022855131534

[47] R Core Team. *R*: A Language and Environment for Statistical Computing. 887 R Foundation for Statistical Computing, Vienna, Austria. https://www.R-project.org. 2017; 888.

[48] Zhao K, Aranzana MJ, Kim S, Lister C, Shindo C, Tang C et al. An *Arabidopsis* example of association mapping in structured samples. PLoS Genet. 2007 Jan 19;3(1):e4. doi: 10.1371/journal.pgen.0030004 17238287 PMC1779303

[49] Pritchard JK, Stephens M, Donnelly P. Inference of population structure using multilocus genotype data. Genetics. 2000 Jun 1;155(2):945–59. doi: 10.1093/genetics/155.2.945 10835412 PMC1461096

[50] Pritchard JK, Donnelly P. Case-control studies of association in structured or admixed populations. Theoretical Population Biology. 2001 Nov 1;60(3):227–37. doi: 10.1006/tpbi.2001.1543 11855957

[51] Pritchard JK, Wen W, Falush D. Documentation for STRUCTURE software: Version 2.3. University of Chicago, Chicago, IL. 2010 Feb 2:1–37. http://pritch.bsd.uchicago.edu/structure.html

[52] Evanno G, Regnaut S, Goudet J. Detecting the number of clusters of individuals using the software STRUCTURE: a simulation study. Molecular Ecology. 2005 Jul;14(8):2611–20. doi: 10.1111/j.1365-294X.2005.02553.x 15969739

[53] Hubisz MJ, Falush D, Stephens M, Pritchard JK. Inferring weak population structure with the assistance of sample group information. Molecular Ecology Resources. 2009 Sep;9(5):1322–32. doi: 10.1111/j.1755-0998.2009.02591.x 21564903 PMC3518025

[54] Perrier X, Jacquemoud-Collet JP. DARwin software. 2006. http://darwin.cirad.fr/

[55] Bradbury PJ, Zhang Z, Kroon DE, Casstevens TM, Ramdoss Y, Buckler ES. TASSEL: software for association mapping of complex traits in diverse samples. Bioinformatics. 2007 Oct 1;23(19):2633–5. doi: 10.1093/bioinformatics/btm308 17586829

[56] Liu Xiaolei, Huang Meng, Fan Bin, Buckler Edward S., and Zhang Zhiwu. Iterative usage of fixed and random effect models for powerful and efficient genome-wide association studies. PLoS Genetics. 2016 Feb 1;12(2):e1005767. doi: 10.1371/journal.pgen.1005767 26828793 PMC4734661

[57] Henderson CR. Best linear unbiased estimation and prediction under a selection model. Biometrics. 1975 Jun 1:423–47. doi: 10.2307/2529430 1174616

[58] Endelman JB, Jannink JL. Shrinkage estimation of the realized relationship matrix. G3: Genes| Genomes| Genetics. 2012 Nov 1;2(11):1405–13. doi: 10.1534/g3.112.004259 23173092 PMC3484671

[59] Cover T, Hart P. Nearest neighbor pattern classification. IEEE Transactions on information theory. 1967 Jan;13(1):21–7. doi: 10.1109/TIT.1967.1053964

[60] Gao X, Becker LC, Becker DM, Starmer JD, Province MA. Avoiding the high Bonferroni penalty in genome-wide association studies. Genetic Epidemiology: The Official Publication of the International Genetic Epidemiology Society. 2010 Jan;34(1):100–5. doi: 10.1002/gepi.20430 19434714 PMC2796708

[61] Utro F, Haiminen N, Livingstone D, Cornejo OE, Royaert S, Schnell RJ et al. iXora: exact haplotype inferencing and trait association. BMC Genetics. 2013 Dec;14(1):1–5. doi: 10.1186/1471-2156-14-48 23742238 PMC3716545

[62] Gutiérrez-López N, Ovando-Medina I, Salvador-Figueroa M, Molina-Freaner F, Avendaño-Arrazate CH, Vázquez-Ovando A. Unique haplotypes of cacao trees as revealed by trnH-psbA chloroplast DNA. PeerJ. 2016 Apr 7;4:e1855. doi: 10.7717/peerj.1855 27076998 PMC4830229

[63] Marcano M, Pugh T, Cros E, Morales S, Páez EA, Courtois B et al. Adding value to cocoa (*Theobroma cacao* L.) germplasm information with domestication history and admixture mapping. Theoretical and Applied Genetics. 2007 Mar 1;114(5):877–84. doi: 10.1007/s00122-006-0486-9 17252253

[64] Motilal LA, Zhang D, Mischke S, Meinhardt LW, Boccara M, Fouet O et al. Association mapping of seed and disease resistance traits in *Theobroma cacao* L. Planta. 2016 Dec;244(6):1265–76. doi: 10.1007/s00425-016-2582-7 27534964

[65] Motilal LA, Sounigo O, Thévenin JM, Risterucci AM, Pieretti I, Noyer JL et al. *Theobroma cacao* L.: genome map and QTLs for *Phytophthora palmivora* resistance. Towards the effective and optimum promotion of cocoa through research and development. Proceedings of the 13th International Cocoa Research Conference; 2000 Oct 9–14; Kota Kinabalu, Malaysia. Lagos: Cocoa Producers’ Alliance; 2001. p. 111–17.

[66] Queiroz VT, Guimarães CT, Ahnert D, Schuster I, Daher RT, Pereira MG et al. Identification of a major QTL in cocoa (*Theobroma cacao* L.) associated with resistance to Witches’ Broom disease. Plant Breeding. 2003 Jun;122(3):268–72. doi: 10.1046/j.1439-0523.2003.00809.x

[67] Royaert S, Phillips-Mora W, Leal AM, Cariaga K, Brown JS, Kuhn DN et al. Identification of marker-trait associations for self-compatibility in a segregating mapping population of *Theobroma cacao* L. Tree Genetics & Genomes. 2011 Dec;7(6):1159–68. doi: 10.1007/s11295-011-0403-5

[68] Royaert S, Jansen J, da Silva DV, de Jesus Branco SM, Livingstone DS, Mustiga G et al. Identification of candidate genes involved in Witches’ Broom disease resistance in a segregating mapping population of *Theobroma cacao* L. in Brazil. BMC Genomics. 2016 Dec;17(1):107. doi: 10.1186/s12864-016-2415-x 26865216 PMC4750280

[69] Sounigo O, Efombagn B, Lemainque A et al. Association mapping on cocoa: a way to identify functional SSR markers linked to yield, tolerance to black pod and mirids assessed in Cameroon and develop a marker assisted breeding programme. Proceedings of the 16th International Cocoa Research Conference; 2009 Nov 16–21; Bali, Indonesia. Lagos: Cocoa Producers’ Alliance; 2012. p.153-8.

[70] Motamayor JC, Mockaitis K, Schmutz J, Haiminen N, Livingstone D III, Cornejo O et al. The genome sequence of the most widely cultivated cacao type and its use to identify candidate genes regulating pod color. Genome Biology. 2013 Jun;14(6):1–25. doi: 10.1186/gb-2013-14-6-r53 23731509 PMC4053823

[71] Da Silva MR, Clément D, Gramacho KP, Monteiro WR, Argout X, Lanaud C et al. Genome-wide association mapping of sexual incompatibility genes in cacao (*Theobroma cacao* L.). Tree Genetics & Genomes. 2016 Jun;12(3):1–3. doi: 10.1007/s11295-016-1012-0

[72] Osorio-Guarín JA, Berdugo-Cely JA, Coronado-Silva RA, Baez E, Jaimes Y, Yockteng R. Genome-wide association study reveals novel candidate genes associated with productivity and disease resistance to *Moniliophthora* spp. in cacao (*Theobroma cacao* L.). G3: Genes, Genomes, Genetics. 2020 May 1;10(5):1713–25. doi: 10.1534/g3.120.401153 32169867 PMC7202020

[73] Semagn K, Bjørnstad Å, Xu Y. The genetic dissection of quantitative traits in crops. Electronic Journal of Biotechnology. 2010 Sep;13(5):16–7. https://scielo.conicyt.cl/pdf/ejb/v13n5/a16.pdf

[74] Mir RR, Choudhary N, Bawa V, Jan S, Singh B, Ashraf Bhat M et al. Allelic diversity, structural analysis and genome-wide association study (GWAS) for yield and related traits using unexplored common bean (*Phaseolus vulgaris* L.) germplasm from Western Himalayas. Frontiers in Genetics. 2021;11:1797. doi: 10.3389/fgene.2020.609603 33584807 PMC7876396

[75] Korte A, Farlow A. The advantages and limitations of trait analysis with GWAS: a review. Plant Methods. 2013 Dec;9(1):1–9. doi: 10.1186/1746-4811-9-29 23876160 PMC3750305

[76] Caspari E. Pleiotropic gene action. Evolution. 1952 March; 6:1–18. https://www.jstor.org/stable/2405500

[77] Yeaman S. Genomic rearrangements and the evolution of clusters of locally adaptive loci. Proceedings of the National Academy of Sciences. 2013 May 7;110(19):E1743–51. Available from: doi: 10.1073/pnas.1219381110 23610436 PMC3651494

[78] Elwers S, Zambrano A, Rohsius C, Lieberei R. Histological features of phenolic compounds in fine and bulk cocoa seed (*Theobroma cacao* L.). J Appl Bot Food Qual. 2010 Sep 1;83(2):182–8.

[79] Bucheli P, Rousseau G, Alvarez M, Laloi M, McCarthy J. Developmental variation of sugars, carboxylic acids, purine alkaloids, fatty acids, and endoproteinase activity during maturation of *Theobroma cacao* L. seeds. Journal of Agricultural and Food Chemistry. 2001 Oct 15;49(10):5046–51. doi: 10.1021/jf010620z 11600064

[80] Mustiga GM, Morrissey J, Stack JC, DuVal A, Royaert S, Jansen J et al. Identification of climate and genetic factors that control fat content and fatty acid composition of *Theobroma cacao* L. beans. Frontiers in Plant Science. 2019 Oct 14;10:1159. doi: 10.1105/tpc.109.068437 31681345 PMC6802002

[81] Amin I, Jinap S, Jamilah B, Harikrisna K, Biehl B. Analysis of vicilin (7S)-class globulin in cocoa cotyledons from various genetic origins. Journal of the Science of Food and Agriculture. 2002 May 15;82(7):728–32. doi: 10.1002/jsfa.1104

[82] Jako C, Kumar A, Wei Y, Zou J, Barton DL, Giblin EM et al. Seed-specific over-expression of an *Arabidopsis* cDNA encoding a diacylglycerol acyltransferase enhances seed oil content and seed weight. Plant Physiology. 2001 Jun 1;126(2):861–74. doi: 10.1104/pp.126.2.861 11402213 PMC111175

[83] Fritz PJ, Fritz KA, Kauffman JM, Patterson GR, Robertson CA, Stoesz DA et al. Cocoa seeds: changes in protein and polysomal RNA during development. Journal of Food Science. 1985 Jul;50(4):946–50. https://onlinelibrary.wiley.com/doi/abs/10.1111/j.1365-2621.1985.tb12986.x

[84] Lung SC, Weselake RJ. Diacylglycerol acyltransferase: a key mediator of plant triacylglycerol synthesis. Lipids. 2006 Dec;41(12):1073–88. doi: 10.1007/s11745-006-5057-y 17269553

[85] Sørensen BM, Furukawa-Stoffer TL, Marshall KS, Page EK, Mir Z, Forster RJ et al. Storage lipid accumulation and acyltransferase action in developing flaxseed. Lipids. 2005 Oct;40(10):1043–9. doi: 10.1007/s11745-005-1467-0 16382576

[86] Perry HJ, Harwood JL. Changes in the lipid content of developing seeds of Brassica napus. Phytochemistry. 1993 Jan 1;32(6):1411–5. doi: 10.1016/0031-9422(93)85148-K

[87] Marcano M, Morales S, Hoyer MT, Courtois B, Risterucci AM, Fouet O et al. A genomewide admixture mapping study for yield factors and morphological traits in a cultivated cocoa (*Theobroma cacao* L.) population. Tree Genetics & Genomes. 2009 Apr 1;5(2):329–37. doi: 10.1007/s11295-008-0185-6

[88] dos Santos Fernandes L, Correa FM, Ingram KT, de Almeida AA, Royaert S. QTL mapping and identification of SNP-haplotypes affecting yield components of *Theobroma cacao* L. Horticulture Research. 2020 Mar 1;7(1):1–8. doi: 10.1038/s41438-020-0250-3 32140235 PMC7049306

[89] Doebley JF, Gaut BS, Smith BD. The molecular genetics of crop domestication. Cell. 2006 Dec 29;127(7):1309–21. doi: 10.1016/j.cell.2006.12.006 17190597

[90] Bhat JA, Ali S, Salgotra RK, Mir ZA, Dutta S, Jadon V et al. Genomic selection in the era of next generation sequencing for complex traits in plant breeding. Frontiers in Genetics. 2016 Dec 27;7:221. https://www.frontiersin.org/articles/10.3389/fgene.2016.00221/full 28083016 10.3389/fgene.2016.00221 PMC5186759

[91] Mangini G, Blanco A, Nigro D, Signorile MA, Simeone R. Candidate genes and quantitative trait loci for grain yield and seed size in Durum Wheat. Plants 2021;10:312–28. doi: 10.3390/plants10020312 33562879 PMC7916090

[92] Sosso D, Luo D, Li QB, Sasse J, Yang J, Gendrot G et al. Seed filling in domesticated maize and rice depends on SWEET-mediated hexose transport. Nature Genetics. 2015 Dec;47(12):1489. doi: 10.1038/ng.3422 26523777

[93] Falcone Ferreyra ML, Rius S, Casati P. Flavonoids: biosynthesis, biological functions, and biotechnological applications. Frontiers in Plant Science. 2012 Sep 28;3:222. doi: 10.3389/fpls.2012.00222 23060891 PMC3460232

[94] Devy L, Anita-Sari I, Saputra TI, Susilo AW, Wachjar A. Identification of molecular marker based on MYB Transcription Factor for the selection of Indonesian Fine Cacao (*Theobroma cacao* L.). Pelita Perkebunan (a Coffee and Cocoa Research Journal). 2018 Aug 31;34(2):59–68.

[95] Liu Y, Shi Z, Maximova SN, Payne MJ, Guiltinan MJ. Tc-MYBPA is an Arabidopsis TT2-like transcription factor and functions in the regulation of proanthocyanidin synthesis in *Theobroma cacao*. BMC Plant Biology. 2015 Dec;15(1):1–6. doi: 10.1186/s12870-015-0529-y 26109181 PMC4481123

[96] Bartley BGD. The genetic diversity of cacao and its utilization. The genetic diversity of cacao and its utilization. Wallingford: CABI Publishing; 2005.

[97] Rockman MV. The QTN program and the alleles that matter for evolution: all that’s gold does not glitter. Evolution: International Journal of Organic Evolution. 2012 Jan;66(1):1–7. doi: 10.1111/j.1558-5646.2011.01486.x 22220860 PMC3386609

[98] Lee S, Abecasis GR, Boehnke M, Lin X. Rare-variant association analysis: study designs and statistical tests. The American Journal of Human Genetics. 2014 Jul 3;95(1):5–23. doi: 10.1016/j.ajhg.2014.06.009 24995866 PMC4085641

[99] Qi Z, Song J, Zhang K, Liu S, Tian X, Wang Y et al. Identification of QTNs controlling 100-seed weight in soybean using multilocus genome-wide association studies. Frontiers in Genetics. 2020 Jul 16;11:689. doi: 10.3389/fgene.2020.00689 32765581 PMC7378803

[100] McKown AD, Klápště J, Guy RD, Geraldes A, Porth I, Hannemann J et al. Genome-wide association implicates numerous genes underlying ecological trait variation in natural populations of *Populus trichocarpa*. New Phytologist. 2014 Jul;203(2):535–53. doi: 10.1111/nph.12815 24750093

[101] Micheli F, Maximova S, Gramacho KP, Guiltinan M, Wilkinson MJ, Lanaud C et al. Functional genomics of cacao. In: Jean-Claude K, Michel D, editors. Advances in Botanical Research, Chapter 3. London: Academic Press; 2010. p.119–177.

[102] Li N, Xu R, Li Y. Molecular networks of seed size control in plants. Annual Review of Plant Biology. 2019 Apr 29;70:435–63. (Table 4). doi: 10.1146/annurev-arplant-050718-095851 30795704

