# Supplementary material for: Genome-wide association studies and genomic selection assays made in a large sample of cacao (Theobroma cacao L.) germplasm reveal significant marker-trait associations and good predictive value for improving yield potential
Source: PLoS One. 2022 Oct 6;17(10):e0260907. doi: 10.1371/journal.pone.0260907 (PMC9536643; doi:10.1371/journal.pone.0260907)
Supplement: S1 Table — http://dx.doi.org/10.13140/RG.2.2.16179.71202. (DOCX) [file pone.0260907.s001.docx]

**S1 Table Background information on the *Theobroma cacao* L. accessions included in this study.**

| **Accession group** | **Number of accessions** | **Origin of group** | **Class** | **Status** |
| --- | --- | --- | --- | --- |
| AMAZ[CHA] | 5 | Ecuador | Upper Amazon Forastero | wild |
| APA | 1 | Colombia | Upper Amazon Forastero | wild |
| BELIZE | 10 | Belize | Criollo | wild |
| CATONGO | 1 | Brazil | Lower Amazon Forastero | cultivated |
| CCN | 1 | Ecuador | Hybrid | cultivated |
| COCA[CHA] | 1 | Ecuador | Upper Amazon Forastero | wild |
| DR | 1 | Indonesia | Criollo/Trinitario | Wild/cultivated |
| EET[ECU] | 1 | Ecuador | Various | cultivated |
| GS | 1 | Grenada | Trinitario | cultivated |
| GU | 13 | French Guiana | Lower Amazon Forastero | wild |
| ICS | 28 | Trinidad | Trinitario | cultivated |
| IF | 2 | Indonesia | unknown | cultivated |
| IMC | 48 | Peru | Upper Amazon Forastero | wild |
| K | 1 | Papua New Guinea | Hybrid | cultivated |
| LAFI | 1 | Western Samoa | Trinitario | cultivated |
| LCTEEN | 59 | Ecuador | Upper Amazon Forastero or Trinitario | wild or cultivated |
| M | 2 | Trinidad | Unclassified | unknown |
| MAN[BRA] | 1 | Brazil | Lower Amazon Forastero | cultivated |
| MATINA | 2 | Costa Rica and Brazil | Mixed/Trinitario | cultivated |
| MO | 5 | Peru | Upper Amazon Forastero | wild |
| NA | 130 | Peru | Upper Amazon Forastero | wild |
| PA | 54 | Peru | Upper Amazon Forastero | wild |
| PLAYA ALTA | 1 | Venezuela | Trinitario | cultivated |
| POUND | 8 | Peru | Upper Amazon Forastero | wild |
| RB[BRA] | 1 | Brazil | Lower Amazon Forastero | wild |
| RIT | 1 | unknown | unknown | unknown |
| SC[COL] | 7 | Colombia | Trinitario or unknown | cultivated |
| SCA | 8 | Peru | Upper Amazon Forastero | wild |
| SIC | 1 | Brazil | Lower Amazon Forastero | cultivated |
| SNK | 4 | Cameroun | Trinitario | cultivated |
| SPA[COL] | 5 | Peru | Hybrid | cultivated |
| SPEC (1-54)  SPEC (55 and upward) | 3  8 | Colombia | Upper Amazon Forastero;  Unknown | wild;  cultivated |
| TSH | 3 | Trinidad and Tobago | Hybrid | cultivated |
| UF | 4 | Costa Rica | Trinitario | cultivated |
