# Supplementary material for: Genome-wide association studies and genomic selection assays made in a large sample of cacao (Theobroma cacao L.) germplasm reveal significant marker-trait associations and good predictive value for improving yield potential
Source: PLoS One. 2022 Oct 6;17(10):e0260907. doi: 10.1371/journal.pone.0260907 (PMC9536643; doi:10.1371/journal.pone.0260907)
Supplement: S3 Table — http://dx.doi.org/10.13140/RG.2.2.22890.59849. (DOCX) [file pone.0260907.s003.docx]

**S3 Table. Results of tests of normality performed on the natural log transformed values of fruit and seed quantitative traits.**

| **Natural log transformed variable** | **Anderson-Darling test of normality*** | **Ryan-Joiner test of normality*** | **Kolmogorov-Smirnov test of normality*** | **Comment** |
| --- | --- | --- | --- | --- |
| Fruit length | >0.578 | >0.10 | >0.15 | use natural log |
| Fruit width | >0.176 | >0.10 | >0.06 | use natural log |
| Fruit length: width | >0.07 | <0.01 | >0.122 | use natural log |
| Total fresh seed weight (g) | >0.80 | >0.10 | >0.15 | use natural log |
| Seed number | >0.06 | >0.10 | <0.04 | use natural log |
| Cotyledon mass (g) | <0.005 | <0.01 | <0.01 | Natural log transformation did not correct deviation from normality |
| Seed/Cotyledon length (cm) | >0.56 | <0.05 | >0.15 | use natural log |
| Seed/Cotyledon width (cm) | >0.09 | >0.01 | <0.03 | use natural log |
| Seed/Cotyledon length to width ratio | >0.124 | >0.10 | >0.136 | use natural log |
| Pod index | >0.30 | >0.10 | >0.15 | use natural log |

**Legend**: * - *P*-values are for tests of normality

**Notes**

Kolmogorov–Smirnov (K-S) and other tests are nonparametric tests of the equality of continuous (or discontinuous), one-dimensional probability distributions, which can be used to compare a sample with a reference probability distribution (for example, one-sample K–S test), or to compare two samples (two-sample K–S test).

- Natural log of Seed/Cotyledon width did not deviate from normality after treating the following as outliers:

LCTEEN-411 (0.85), NA286 (0.73), NA47 (0.89), NA712 (0.88), PA218 (0.76), SCA11 (0.91) and SCA6 (0.88). These accessions had low values.

- Seed/Cotyledon length to width ratio:

The value for NA21 (1.00 cm) was relatively too small and PA218 (2.55 cm) was too large (outliers).

These extreme values were treated as missing. After that, natural log transformation corrected the deviation from normality.

- Cotyledon mass – this entry behaved like a mixture distribution.
