# Supplementary material for: Genome-wide association studies and genomic selection assays made in a large sample of cacao (Theobroma cacao L.) germplasm reveal significant marker-trait associations and good predictive value for improving yield potential
Source: PLoS One. 2022 Oct 6;17(10):e0260907. doi: 10.1371/journal.pone.0260907 (PMC9536643; doi:10.1371/journal.pone.0260907)
Supplement: S4 Table — http://dx.doi.org/10.13140/RG.2.2.36312.37128. (DOCX) [file pone.0260907.s004.docx]

**S4 Table. Descriptive statistics for quantitative fruit and seed traits in wild, cultivated and unclassified cacao germplasm studied.**

| **Variable** | **Type of germplasm** | **Total Count** | **Mean** | **Standard Error of Mean** | **Coefficient of Variation** | | **Minimum** | | **Maximum** | | **Skewness** | |  | |
| --- | --- | --- | --- | --- | --- | --- | --- | --- | --- | --- | --- | --- | --- | --- |
| Seed number | Wild -1 | 263 | 40.194 | 0.428 | 17.27 | | 22.00 | | 59.00 | | 0.26 | |  | |
|  | Cultivated -2 | 49 | 37.429 | 0.836 | 15.64 | | 26.00 | | 54.00 | | 0.97 | |  | |
|  | Unclassified – 3 | 34 | 37.71 | 1.05 | 16.24 | | 23.00 | | 48.00 | | -0.62 | |  | |
|  |  |  |  |  |  | |  | |  | |  | |  | |
| Individual dried cotyledon mass (g) | 1 | 263 | 0.877 | 0.0097 | 18.00 | | 0.44 | | 1.49 | | 0.44 | |  | |
|  | 2 | 49 | 1.154 | 0.039 | 23.42 | | 0.62 | | 1.84 | | 0.00 | |  | |
|  | 3 | 34 | 0.920 | 0.041 | 25.90 | | 0.56 | | 1.51 | | 0.55 | |  | |
|  |  |  |  |  |  | |  | |  | |  | |  | |
| Cotyledon length (cm) | 1 | 263 | 2.056 | 0.012 | 9.71 | | 1.37 | | 2.69 | | 0.21 | |  | |
|  | 2 | 49 | 2.319 | 0.026 | 7.75 | | 1.92 | | 2.72 | | 0.04 | |  | |
|  | 3 | 34 | 2.158 | 0.029 | 7.85 | | 1.88 | | 2.58 | | 0.47 | |  | |
|  |  |  |  |  |  | |  | |  | |  | |  | |
| Cotyledon width (cm) | 1 | 263 | 1.159 | 0.0064 | 9.00 | | 0.73 | | 1.41 | | -0.64 | |  | |
|  | 2 | 49 | 1.317 | 0.019 | 10.14 | | 0.93 | | 1.56 | | -0.71 | |  | |
|  | 3 | 34 | 1.178 | 0.020 | 9.91 | | 0.83 | | 1.34 | | -0.91 | |  | |
|  |  |  |  |  |  | |  | |  | |  | |  | |
| Cotyledon length to width ratio | 1 | 263 | 1.781 | 0.011 | 10.11 | | 1.00 | | 2.55 | | 0.23 | |  | |
|  | 2 | 49 | 1.769 | 0.019 | 7.55 | | 1.54 | | 2.20 | | **1.00** | |  | |
|  | 3 | 34 | 1.845 | 0.030 | 9.61 | | 1.52 | | 2.30 | | 0.48 | |  | |
|  |  |  |  |  |  | |  | |  | |  | |  | |
| Pod index | 1 | 263 | 30.122 | 0.46 | 24.96 | | 15.98 | | 66.14 | | **1.10** | |  | |
|  | 2 | 49 | 24.803 | 0.89 | 25.04 | | 13.94 | | 41.36 | | 0.93 | |  | |
|  | 3 | 34 | 31.49 | 1.65 | 30.61 | | 16.49 | | 54.82 | | 0.86 | |  | |
|  |  |  | |  |  |  | |  | |  | |  | |  |
| Substantial skewness is indicated in bold | | | | | | | | | | | | | |  |
