# Supplementary material for: Genome-wide association studies and genomic selection assays made in a large sample of cacao (Theobroma cacao L.) germplasm reveal significant marker-trait associations and good predictive value for improving yield potential
Source: PLoS One. 2022 Oct 6;17(10):e0260907. doi: 10.1371/journal.pone.0260907 (PMC9536643; doi:10.1371/journal.pone.0260907)
Supplement: S5 Table — http://dx.doi.org/10.13140/RG.2.2.17438.00328. (DOCX) [file pone.0260907.s005.docx]

**S5 Table. ANOVA for yield-related traits.**

| **Traits** | **Source** | **DF** | **Adjusted**  **Sum of Squares** | **Adjusted**  **Mean Square** | **F-Value** | ***P*-value** |
| --- | --- | --- | --- | --- | --- | --- |
| Ovule number | Type  (wild versus cultivated or unclassified ) | 2 | 0.7503 | 0.37513 | 24.15 | 0.0001*** |
|  | Residual | 343 | 5.3287 | 0.01554 |  |  |
|  | Total | 345 | 6.0790 |  |  |  |
| Fruit length ((cm) | Type | 2 | 0.04253 | 0.02127 | 1.65 | 0.194 |
|  | Residual | 343 | 4.42558 | 0.01290 |  |  |
|  | Total | 345 | 4.46812 |  |  |  |
| Fruit width (cm) | Type | 2 | 0.1921 | 0.096045 | 10.29 | 0.0001*** |
|  | Residual | 343 | 3.2016 | 0.009334 |  |  |
|  | Total | 345 | 3.3937 |  |  |  |
| Fruit length to width ratio | Type | 2 | 0.07574 | 0.03787 | 3.40 | 0.034* |
|  | Residual | 343 | 3.82030 | 0.01114 |  |  |
|  | Total | 345 | 3.89604 |  |  |  |
| Total fresh seed mass (g) | Type | 2 | 2.493 | 1.24648 | 20.23 | 0.0001*** |
|  | Residual | 343 | 21.134 | 0.06161 |  |  |
|  | Total | 345 | 23.627 |  |  |  |
| Individual dried cotyledon mass (g) | Type | 2 | 2.834 | 1.41720 | 34.84 | 0.0001*** |
|  | Residual | 343 | 13.952 | 0.04068 |  |  |
|  | Total | 345 | 16.786 |  |  |  |
| Seed/Cotyledon length (cm) | Type | 2 | 0.6487 | 0.324346 | 37.28 | 0.0001*** |
|  | Residual | 343 | 2.9842 | 0.008700 |  |  |
|  | Total | 345 | 3.6328 |  |  |  |
| Seed/Cotyledon width (cm) | Type | 2 | 0.6559 | 0.327946 | 34.81 | 0.0001*** |
|  | Residual | 343 | 3.2314 | 0.009421 |  |  |
|  | Total | 345 | 3.8873 |  |  |  |
| Seed/Cotyledon length to width ratio | Type | 2 | 0.04153 | 0.020764 | 2.16 | 0.116 |
|  | Residual | 343 | 3.29051 | 0.009593 |  |  |
|  | Total | 345 | 3.33204 |  |  |  |
| Seed number | Type | 2 | 0.2732 | 0.13660 | 4.64 | 0.010* |
|  | Residual | 343 | 10.1000 | 0.02945 |  |  |
|  | Total | 345 | 10.3732 |  |  |  |
| Pod index | Type | 2 | 1.674 | 0.83705 | 14.07 | 0.0001*** |
|  | Residual | 343 | 20.406 | 0.05949 |  |  |
|  | Total | 345 | 22.080 |  |  |  |
