# Supplementary material for: Genome-wide association studies and genomic selection assays made in a large sample of cacao (Theobroma cacao L.) germplasm reveal significant marker-trait associations and good predictive value for improving yield potential
Source: PLoS One. 2022 Oct 6;17(10):e0260907. doi: 10.1371/journal.pone.0260907 (PMC9536643; doi:10.1371/journal.pone.0260907)
Supplement: S6 Table — http://dx.doi.org/10.13140/RG.2.2.27504.33282. (DOCX) [file pone.0260907.s006.docx]

**S6 Table. Coefficients of membership for clusters of accessions based on *STRUCTURE* analysis**

| **Accession** | **PA** | **LCT_EEN, SCA, MO** | **NA, POUND** | **SPEC** | **CRIOLLO** | **GU** | **AMAZ, IMC** |
| --- | --- | --- | --- | --- | --- | --- | --- |
| AMAZ12 | 0.004 | 0.16 | 0.299 | 0.141 | 0.003 | 0.002 | 0.391 |
| AMAZ15 | 0.008 | 0.25 | 0.035 | 0.132 | 0.002 | 0.002 | 0.57 |
| AMAZ6 | 0.004 | 0.304 | 0.018 | 0.168 | 0.001 | 0.008 | 0.496 |
| AMAZ6_3 | 0.003 | 0.22 | 0.004 | 0.183 | 0.004 | 0.005 | 0.581 |
| AMAZ9 | 0.003 | 0.43 | 0.008 | 0.133 | 0.006 | 0.002 | 0.417 |
| APA4 | 0.001 | 0 | 0.224 | **0.772** | 0 | 0.002 | 0.001 |
| BELIZE97-61_B-2-C10-2 | 0.001 | 0.002 | 0.001 | 0.001 | **0.975** | 0.013 | 0.008 |
| BELIZE9717_B-1 | 0 | 0 | 0 | 0 | **0.999** | 0 | 0 |
| BELIZE9761_B-1 | 0 | 0 | 0 | 0 | **0.999** | 0 | 0 |
| BELIZE9774_7-4 | 0 | 0 | 0 | 0 | **0.999** | 0 | 0 |
| BELIZE97C_6-4 | 0 | 0 | 0 | 0 | **0.999** | 0 | 0 |
| BELIZE97C_C1 | 0 | 0 | 0 | 0 | **0.999** | 0 | 0 |
| BELIZE97C_C6 | 0 | 0 | 0 | 0 | **0.999** | 0 | 0 |
| BELIZE97C_D-1 | 0 | 0 | 0 | 0 | **0.999** | 0 | 0 |
| BELIZE97EX1C_3 | 0 | 0.001 | 0 | 0 | **0.998** | 0 | 0 |
| BELIZE97ST1_1 | 0 | 0 | 0 | 0 | **0.999** | 0 | 0 |
| CATONGO | 0.001 | 0 | 0 | 0.996 | 0 | 0.001 | 0 |
| CCN51 | 0.015 | 0.006 | 0.002 | **0.282** | 0.151 | 0.002 | **0.542** |
| COCA3370_5 | 0.031 | 0.246 | 0.044 | 0.185 | 0.088 | 0.002 | **0.403** |
| DR1 | 0.001 | 0.001 | 0.001 | 0.527 | 0.468 | 0.001 | 0.001 |
| EET-95 | 0.002 | **0.519** | 0.002 | **0.47** | 0.002 | 0.002 | 0.003 |
| GS36 | 0.01 | 0.007 | 0.004 | 0.493 | 0.472 | 0.006 | 0.007 |
| GU114-P | 0.001 | 0 | 0 | 0 | 0 | **0.997** | 0 |
| GU151-F | 0.001 | 0 | 0 | 0 | 0 | **0.997** | 0 |
| GU175-P | 0 | 0 | 0 | 0 | 0 | **0.998** | 0 |
| GU195-P | 0.001 | 0 | 0 | 0 | 0 | **0.998** | 0 |
| GU219-P | 0.001 | 0 | 0 | 0 | 0 | **0.998** | 0 |
| GU241-P | 0.001 | 0 | 0.001 | 0 | 0 | **0.997** | 0 |
| GU261-P | 0.001 | 0 | 0 | 0 | 0 | **0.997** | 0 |
| GU265-P | 0.001 | 0 | 0 | 0 | 0 | **0.998** | 0 |
| GU277-G | 0.001 | 0 | 0 | 0 | 0 | **0.998** | 0 |
| GU286 | 0.002 | 0 | 0.003 | 0.001 | 0 | **0.99** | 0.003 |
| GU300-P | 0.001 | 0 | 0 | 0 | 0 | **0.998** | 0 |
| GU305-P | 0.018 | **0.503** | 0.038 | 0.389 | 0.018 | **0.015** | 0.02 |
| GU307-P | 0.001 | 0 | 0 | 0 | 0 | **0.997** | 0 |
| GU310-P | 0.005 | 0 | 0.001 | 0.005 | 0 | **0.987** | 0.001 |
| GU353P | 0.001 | 0 | 0 | 0 | 0 | **0.998** | 0 |
| ICS1 | 0.002 | 0.001 | 0.001 | **0.698** | **0.297** | 0.001 | 0.001 |
| ICS1 | 0.002 | 0.001 | 0.001 | **0.698** | **0.297** | 0.001 | 0.001 |
| **ICS10** | 0.002 | 0.336 | 0.001 | **0.001** | **0.001** | 0.002 | **0.658** |
| ICS100 | 0.003 | 0.001 | 0.002 | **0.51** | **0.483** | 0.001 | 0.001 |
| ICS15 | 0.002 | 0.001 | 0.001 | **0.671** | **0.322** | 0.002 | 0.001 |
| ICS25 | 0.002 | 0.001 | 0.001 | **0.643** | **0.351** | 0.001 | 0.001 |
| **ICS30** | 0.004 | 0.001 | 0.006 | **0.4** | **0.002** | 0.001 | **0.586** |
| ICS39 | 0.001 | 0.001 | 0.001 | **0.498** | **0.497** | 0.001 | 0.001 |
| **ICS40** | **0.379** | 0.047 | 0.001 | **0.002** | **0.005** | 0.002 | **0.564** |
| ICS45 | 0.002 | 0.001 | 0.001 | **0.493** | **0.502** | 0.001 | 0.001 |
| ICS47 | 0.002 | 0.001 | 0.001 | **0.438** | **0.556** | 0.001 | 0.001 |
| ICS48 | 0.002 | 0.001 | 0.001 | **0.493** | **0.501** | 0.001 | 0.001 |
| ICS49 | 0.003 | 0.001 | 0.001 | **0.774** | **0.22** | 0.001 | 0.001 |
| ICS6 | 0.002 | 0.001 | 0.001 | **0.739** | **0.255** | 0.001 | 0.001 |
| ICS60 | 0.001 | 0.001 | 0.001 | **0.471** | **0.524** | 0.001 | 0.001 |
| **ICS62** | 0.005 | **0.375** | 0.09 | **0.001** | **0.001** | 0.002 | **0.526** |
| ICS63 | 0.001 | 0.001 | 0.001 | **0.696** | **0.3** | 0.001 | 0.001 |
| ICS66 | 0.003 | 0.001 | 0.001 | **0.646** | **0.347** | 0.001 | 0.001 |
| ICS70 | 0.05 | 0.541 | 0.022 | **0.355** | **0.015** | 0.003 | 0.013 |
| ICS72 | 0.002 | 0.001 | 0.001 | **0.57** | **0.425** | 0.001 | 0.001 |
| ICS75 | 0.011 | 0.003 | 0.008 | **0.591** | **0.34** | 0.022 | 0.025 |
| ICS76 | 0.002 | 0.001 | 0.001 | **0.637** | **0.357** | 0.001 | 0.001 |
| ICS8 | 0.002 | 0.001 | 0.001 | **0.782** | **0.211** | 0.001 | 0.001 |
| **ICS80** | 0.143 | 0.036 | 0.007 | **0.177** | **0.152** | 0.006 | **0.48** |
| ICS81 | 0.004 | 0.004 | 0.003 | **0.55** | **0.43** | 0.004 | 0.004 |
| ICS81 | 0.014 | 0.005 | 0.003 | **0.628** | **0.341** | 0.005 | 0.004 |
| ICS84 | 0.001 | 0.001 | 0.001 | **0.629** | **0.366** | 0.001 | 0.001 |
| ICS86 | 0.005 | 0.004 | 0.008 | **0.644** | **0.329** | 0.002 | 0.009 |
| ICS89 | 0.002 | 0.001 | 0.001 | **0.554** | **0.441** | 0.001 | 0.001 |
| ICS95 | 0.002 | 0.001 | 0.001 | **0.462** | **0.532** | 0.001 | 0.001 |
| IF5 | 0.002 | 0.001 | 0.001 | 0.819 | 0.175 | 0.001 | 0.001 |
| IFC1 | 0.001 | 0.002 | 0.001 | 0.82 | 0.175 | 0.001 | 0.001 |
| IMC10 | 0.001 | 0.001 | 0.001 | 0.001 | 0 | 0.001 | **0.996** |
| IMC105 | 0.001 | 0.231 | 0.001 | 0.001 | 0.002 | 0.002 | **0.762** |
| IMC107 | 0.001 | 0.001 | 0.001 | 0.001 | 0.001 | 0.001 | **0.995** |
| **IMC13** | 0.012 | 0.465 | 0.058 | 0.274 | 0.007 | 0.021 | 0.163 |
| IMC14 | 0.001 | 0.001 | 0.001 | 0.001 | 0 | 0.001 | **0.995** |
| **IMC16** | 0.001 | 0 | 0.997 | 0.001 | 0 | 0 | **0.001** |
| IMC18 | 0.006 | 0.001 | 0.003 | 0.003 | 0.001 | 0.001 | **0.985** |
| IMC2 | 0.001 | 0.195 | 0.001 | 0.001 | 0.001 | 0.001 | **0.8** |
| IMC20 | 0.001 | 0.003 | 0.001 | 0 | 0.002 | 0.001 | **0.993** |
| IMC23 | 0.001 | 0 | 0.001 | 0.001 | 0 | 0.001 | **0.995** |
| IMC27 | 0.001 | 0 | 0.001 | 0.001 | 0 | 0.001 | **0.996** |
| **IMC3** | 0.005 | 0.003 | 0.001 | **0.505** | **0.474** | 0.005 | **0.006** |
| IMC31 | 0.001 | 0.001 | 0.001 | 0.001 | 0.001 | 0.001 | **0.995** |
| IMC33 | 0.001 | 0.001 | 0.001 | 0 | 0.001 | 0.001 | **0.995** |
| IMC36 | 0.002 | 0.271 | 0.004 | 0.283 | 0.004 | 0.002 | **0.435** |
| **IMC38** | 0.108 | **0.852** | 0.006 | 0.001 | 0.001 | 0.022 | **0.01** |
| IMC39 | 0.001 | 0.001 | 0.001 | 0.001 | 0.001 | 0.001 | **0.996** |
| IMC41 | **0.994** | 0 | 0.001 | 0.002 | 0 | 0.001 | **0.001** |
| IMC42 | 0.001 | 0.001 | 0.001 | 0.001 | 0.001 | 0.001 | **0.996** |
| IMC45 | 0.002 | 0.258 | 0.001 | 0.001 | 0.001 | 0.001 | **0.736** |
| IMC47 | 0.02 | **0.494** | 0.015 | 0.439 | 0.004 | 0.005 | **0.023** |
| IMC48 | 0.001 | 0 | 0.002 | 0.001 | 0 | 0.001 | **0.995** |
| IMC49 | 0.003 | 0.183 | 0.002 | 0.001 | 0.001 | 0.007 | **0.803** |
| IMC5 | 0.001 | 0.001 | 0.001 | 0.001 | 0 | 0.001 | **0.996** |
| IMC50 | 0.001 | 0.001 | 0.001 | 0.001 | 0.001 | 0.001 | **0.995** |
| IMC51 | 0.003 | 0.221 | 0.001 | 0.001 | 0.002 | 0.002 | **0.771** |
| IMC53 | 0.036 | 0.124 | 0.044 | 0.054 | 0.007 | 0.031 | **0.704** |
| IMC54 | 0.002 | 0.231 | 0.001 | 0.001 | 0.003 | 0.003 | **0.759** |
| IMC55 | 0.001 | 0 | 0.002 | 0.001 | 0 | 0.001 | **0.994** |
| IMC57 | 0.001 | 0.216 | 0.002 | 0.001 | 0.001 | 0.006 | **0.773** |
| IMC58 | 0.001 | 0 | 0.001 | 0.001 | 0 | 0.001 | **0.996** |
| IMC59 | 0.001 | 0 | 0.002 | 0.001 | 0 | 0.001 | **0.995** |
| IMC6 | 0.001 | 0.001 | 0.001 | 0.001 | 0.001 | 0.001 | **0.995** |
| IMC60 | 0.001 | 0.001 | 0.001 | 0.001 | 0.001 | 0.001 | **0.996** |
| IMC61 | 0.001 | 0.001 | 0.001 | 0.001 | 0.001 | 0.001 | **0.996** |
| IMC63 | 0.001 | 0.001 | 0.001 | 0.001 | 0.001 | 0.001 | **0.996** |
| IMC65 | 0.001 | 0 | 0.001 | 0.001 | 0 | 0.001 | **0.996** |
| IMC66 | 0.002 | 0.033 | 0.001 | 0.001 | 0.002 | 0.003 | **0.958** |
| **IMC67** | 0.005 | 0.024 | **0.579** | 0.008 | 0.006 | 0.001 | 0.378 |
| IMC68 | 0.005 | 0.277 | 0.005 | 0.01 | 0.006 | 0.056 | **0.642** |
| IMC71 | 0.002 | 0.001 | 0.001 | 0.001 | 0 | 0.001 | **0.994** |
| IMC73 | 0.002 | 0.201 | 0.003 | 0.001 | 0.001 | 0.004 | **0.789** |
| IMC76 | 0.001 | 0.002 | 0.001 | 0 | 0.001 | 0.001 | **0.995** |
| IMC77 | 0.001 | 0 | 0.001 | 0.001 | 0 | 0.001 | **0.996** |
| IMC78 | 0.001 | 0 | 0.002 | 0.001 | 0 | 0.001 | **0.995** |
| **IMC83** | 0.079 | **0.521** | 0.013 | **0.363** | 0.007 | 0.001 | **0.016** |
| IMC9 | 0.002 | 0.322 | 0.002 | 0.001 | 0.001 | 0.002 | **0.671** |
| IMC94 | 0.001 | 0.062 | 0.001 | 0.001 | 0.009 | 0.004 | **0.922** |
| IMC96 | 0.002 | 0.003 | 0.001 | 0.001 | 0.002 | 0.002 | **0.989** |
| IMC97 | 0.001 | 0 | 0.001 | 0.001 | 0 | 0.001 | **0.996** |
| IMC98 | 0.001 | 0.001 | 0.001 | 0.001 | 0.001 | 0.001 | **0.995** |
| K5 | 0.001 | 0.001 | 0.001 | **0.817** | 0.178 | 0.001 | 0.001 |
| LAF1 | 0.001 | 0.001 | 0.001 | 0.272 | **0.723** | 0.001 | 0.001 |
| LCTEEN-188 | 0.001 | **0.84** | 0 | 0 | 0.158 | 0 | 0.001 |
| LCTEEN-193 | 0.001 | **0.983** | 0 | 0 | 0.015 | 0 | 0 |
| LCTEEN-220 | 0.012 | **0.953** | 0.002 | 0.002 | 0.011 | 0.018 | 0.002 |
| LCTEEN-261/S_4 | 0.003 | **0.486** | 0.003 | 0.479 | 0.024 | 0.001 | 0.003 |
| LCTEEN-63 | 0 | **0.963** | 0 | 0 | 0.036 | 0 | 0 |
| LCTEEN-82 | 0.002 | **0.84** | 0.003 | 0.001 | 0.001 | 0.001 | 0.152 |
| LCTEEN-84 | 0.002 | **0.992** | 0.002 | 0.001 | 0.001 | 0.001 | 0.001 |
| LCTEEN-122 | 0 | **0.941** | 0 | 0 | 0.057 | 0 | 0 |
| LCTEEN-127 | 0.001 | **0.652** | 0.001 | 0.322 | 0.018 | 0.002 | 0.004 |
| LCTEEN-162_S1010 | **0.328** | **0.001** | 0.163 | 0.009 | 0.001 | 0.003 | **0.496** |
| LCTEEN-163 | 0 | **0.942** | 0 | 0 | 0.056 | 0 | 0 |
| LCTEEN-163_A | 0 | **0.993** | 0 | 0 | 0.005 | 0 | 0 |
| LCTEEN-189 | 0.001 | **0.84** | 0 | 0 | 0.158 | 0 | 0.001 |
| LCTEEN-195 | 0.002 | **0.876** | 0.004 | 0.002 | 0.104 | 0.003 | 0.009 |
| LCTEEN-202 | 0.003 | **0.759** | 0.003 | 0.22 | 0.008 | 0.001 | 0.006 |
| LCTEEN-21_S4 | 0.002 | **0.236** | 0.001 | 0.747 | 0.009 | 0.001 | 0.005 |
| LCTEEN-212_S4 | 0.005 | **0.032** | 0.003 | 0.877 | 0.031 | 0.019 | 0.033 |
| LCTEEN-218 | 0 | **0.988** | 0 | 0 | 0.011 | 0 | 0 |
| LCTEEN-221 | 0.002 | **0.603** | 0.002 | 0.374 | 0.005 | 0.007 | 0.008 |
| LCTEEN-227 | 0 | **0.852** | 0 | 0 | 0.146 | 0 | 0 |
| LCTEEN-23 | 0.011 | **0.71** | 0.005 | 0.256 | 0.012 | 0.003 | 0.004 |
| LCTEEN-246 | 0.023 | **0.011** | 0.008 | 0.528 | 0.123 | 0.008 | 0.299 |
| LCTEEN-249 | 0.004 | **0.99** | 0.001 | 0.001 | 0.002 | 0.002 | 0.001 |
| LCTEEN-251 | 0.001 | **0.995** | 0 | 0 | 0.003 | 0 | 0 |
| LCTEEN-255 | 0.001 | **0.89** | 0.001 | 0.001 | 0.105 | 0.001 | 0.001 |
| LCTEEN-267 | 0.001 | **0.992** | 0 | 0 | 0.005 | 0.001 | 0 |
| LCTEEN-278 | 0.009 | **0.148** | 0.004 | 0.279 | 0.004 | 0.004 | 0.551 |
| LCTEEN-280 | 0.003 | **0.924** | 0.003 | 0.048 | 0.008 | 0.009 | 0.004 |
| LCTEEN-30 | 0 | **0.997** | 0 | 0 | 0.001 | 0.001 | 0 |
| LCTEEN-312 | 0 | **0.73** | 0 | 0 | 0.268 | 0 | 0 |
| LCTEEN-32 | 0.009 | **0.957** | 0.003 | 0.004 | 0.002 | 0.003 | 0.022 |
| LCTEEN-325 | 0.004 | **0.882** | 0.001 | 0.006 | 0.099 | 0 | 0.008 |
| LCTEEN-326 | 0.001 | **0.002** | 0.011 | 0.716 | 0.001 | 0.001 | 0.266 |
| LCTEEN-327 | 0 | **0.981** | 0 | 0 | 0.018 | 0 | 0 |
| LCTEEN-332 | 0 | **0.826** | 0 | 0 | 0.172 | 0 | 0 |
| LCTEEN-333 | 0 | **0.839** | 0 | 0 | 0.159 | 0 | 0 |
| LCTEEN-36 | 0 | **0.997** | 0 | 0 | 0.001 | 0 | 0 |
| LCTEEN-362 | 0.004 | **0.723** | 0.006 | 0.002 | 0.247 | 0.011 | 0.008 |
| LCTEEN-368 | 0.002 | **0.796** | 0.001 | 0.001 | 0.197 | 0.002 | 0.001 |
| LCTEEN-37 | 0.035 | **0.826** | 0.058 | 0.005 | 0.002 | 0.009 | 0.064 |
| LCTEEN-372 | 0 | **0.993** | 0 | 0 | 0.006 | 0 | 0 |
| LCTEEN-403 | 0.001 | **0.983** | 0 | 0 | 0.015 | 0.001 | 0.001 |
| LCTEEN-409 | 0.001 | **0** | 0.224 | 0.772 | 0 | 0.001 | 0.001 |
| LCTEEN-411 | 0.001 | **0.799** | 0 | 0 | 0.199 | 0 | 0.001 |
| LCTEEN-413 | 0.011 | **0.955** | 0.002 | 0.002 | 0.012 | 0.017 | 0.002 |
| LCTEEN-46 | 0.021 | **0.49** | 0.01 | 0.306 | 0.055 | 0.075 | 0.044 |
| LCTEEN-57 | 0 | **0.921** | 0 | 0 | 0.077 | 0 | 0 |
| LCTEEN-60 | 0.21 | **0.285** | 0.008 | 0.004 | 0.001 | 0.004 | 0.488 |
| LCTEEN-62_S4 | 0 | **0** | 0 | 0.998 | 0 | 0 | 0 |
| LCTEEN-68_S2 | 0.043 | **0.012** | 0.007 | 0.514 | 0.115 | 0.007 | 0.302 |
| LCTEEN-73A | 0.081 | **0.805** | 0.027 | 0.066 | 0.002 | 0.002 | 0.017 |
| LCTEEN-81 | 0.001 | **0.996** | 0 | 0 | 0.001 | 0.001 | 0.001 |
| LCTEEN-83 | 0.008 | **0.834** | 0.005 | 0.031 | 0.001 | 0.002 | 0.119 |
| LCTEEN-86 | 0 | **0.997** | 0 | 0 | 0.001 | 0 | 0 |
| LCTEEN-90_S7 | 0 | **0.001** | 0.005 | 0.993 | 0.001 | 0 | 0 |
| LCTEEN-91 | 0 | **0.997** | 0 | 0 | 0 | 0.001 | 0 |
| LCTEEN15_S3 | 0.023 | **0.77** | 0.002 | 0.187 | 0.006 | 0.002 | 0.011 |
| LCTEEN20S10 | 0.038 | **0.662** | 0.002 | 0.023 | 0.064 | 0.003 | 0.208 |
| LCTEEN66 | 0 | **0.997** | 0 | 0 | 0.001 | 0 | 0 |
| LCTEEN6S1 | 0.012 | **0.706** | 0.005 | 0.266 | 0.004 | 0.003 | 0.005 |
| M33 | 0.109 | **0.496** | 0.02 | **0.356** | 0.006 | 0.004 | 0.011 |
| M8 | 0.006 | 0 | 0.231 | **0.748** | 0.001 | 0.002 | 0.012 |
| MAN15_60 | **0.345** | 0.093 | 0.196 | 0.116 | 0.074 | 0.005 | 0.172 |
| Mat1-6 | 0 | 0 | 0 | **0.998** | 0 | 0 | 0 |
| MATINA1_7 | 0.004 | 0.009 | 0.001 | 0.185 | 0.297 | 0.002 | **0.502** |
| MO121 | 0.001 | **0.997** | 0 | 0 | 0.001 | 0.001 | 0.001 |
| MO76 | 0.002 | **0.985** | 0.001 | 0.001 | 0.005 | 0.004 | 0.001 |
| MO83 | **0.994** | 0 | 0.001 | 0.001 | 0 | 0.002 | 0.001 |
| MO96 | 0 | **0.997** | 0 | 0 | 0.001 | 0.001 | 0 |
| MO99 | 0.001 | **0.995** | 0.001 | 0.001 | 0.001 | 0.001 | 0.001 |
| NA1 | 0.004 | 0.116 | **0.486** | 0.003 | 0.003 | 0.009 | 0.379 |
| NA104 | 0.005 | 0.135 | **0.133** | 0.212 | 0.006 | 0.129 | 0.379 |
| NA110 | 0.008 | 0.169 | **0.496** | 0.002 | 0.001 | 0.003 | 0.322 |
| NA111 | 0.003 | 0.086 | **0.749** | 0.013 | 0.073 | 0.002 | 0.074 |
| NA112 | 0.003 | 0.001 | **0.965** | 0.014 | 0 | 0.001 | 0.016 |
| NA113 | 0.003 | 0.007 | **0.575** | 0.002 | 0.008 | 0.002 | 0.404 |
| NA114 | 0.003 | 0.001 | **0.001** | **0.759** | 0.234 | 0.001 | 0.001 |
| NA127 | 0 | 0 | **0.998** | 0 | 0 | 0 | 0 |
| NA129 | 0 | 0 | **0.998** | 0 | 0 | 0 | 0 |
| NA13 | 0.005 | 0.002 | **0.583** | 0.013 | 0.001 | 0.001 | 0.394 |
| NA137 | 0.002 | 0.031 | **0.001** | 0.001 | 0.001 | 0.003 | 0.96 |
| NA14 | 0.011 | 0.212 | **0.441** | 0.023 | 0.005 | 0.036 | 0.271 |
| NA140 | 0.03 | 0.3 | **0.004** | 0.64 | 0.001 | 0.022 | 0.002 |
| NA141 | 0.007 | 0.002 | **0.653** | 0.01 | 0.002 | 0.008 | 0.318 |
| NA142 | 0.001 | 0.001 | **0.001** | 0.778 | 0.217 | 0.001 | 0.001 |
| NA144 | 0.006 | 0.052 | **0.714** | 0.001 | 0.09 | 0.005 | 0.132 |
| NA145 | 0.001 | 0.001 | **0.001** | 0.001 | 0.001 | 0.001 | 0.995 |
| NA149 | 0.001 | 0.148 | **0.845** | 0 | 0.001 | 0.001 | 0.004 |
| NA154 | 0.002 | 0.072 | **0.916** | 0.001 | 0.003 | 0.001 | 0.005 |
| NA157 | 0.197 | 0.486 | **0.005** | 0.299 | 0.003 | 0.002 | 0.008 |
| NA159 | 0.003 | 0.001 | **0.001** | 0.672 | 0.32 | 0.001 | 0.002 |
| NA168 | 0.005 | 0.142 | **0.803** | 0.018 | 0.003 | 0.002 | 0.027 |
| NA170 | 0.004 | 0.005 | **0.581** | 0.007 | 0.003 | 0.003 | 0.398 |
| NA176 | 0.985 | 0.001 | **0.002** | 0.01 | 0.001 | 0.001 | 0.001 |
| NA178 | 0 | 0 | **0.998** | 0 | 0 | 0 | 0 |
| NA181 | 0.003 | 0.12 | **0.52** | 0.001 | 0.096 | 0.001 | 0.259 |
| NA183 | 0.007 | 0.056 | **0.669** | 0.002 | 0.046 | 0.002 | 0.218 |
| NA184 | 0 | 0 | **0.998** | 0 | 0 | 0 | 0 |
| NA187 | 0.004 | 0.001 | **0.68** | 0.004 | 0.001 | 0.07 | 0.24 |
| NA189 | 0.004 | 0.027 | **0.613** | 0.001 | 0.119 | 0.002 | 0.234 |
| NA19 | 0.002 | 0.001 | **0.894** | 0.034 | 0 | 0.001 | 0.069 |
| NA191 | 0.003 | 0 | **0.882** | 0.038 | 0 | 0.001 | 0.075 |
| NA194 | 0 | 0 | **0.998** | 0 | 0 | 0 | 0 |
| NA204 | 0 | 0 | **0.998** | 0 | 0 | 0 | 0 |
| NA206 | 0.004 | 0.001 | **0.664** | 0.018 | 0 | 0.001 | 0.311 |
| NA21 | 0.005 | 0.781 | **0.003** | 0.203 | 0.002 | 0.003 | 0.003 |
| NA218 | 0.09 | 0.398 | **0.018** | 0.254 | 0.002 | 0.002 | 0.235 |
| NA223 | 0.004 | 0.561 | **0.001** | 0.429 | 0.001 | 0.003 | 0.001 |
| NA226 | 0.003 | 0.001 | **0.821** | 0.002 | 0 | 0.002 | 0.171 |
| NA227 | 0.002 | 0.18 | **0.694** | 0.005 | 0.113 | 0.001 | 0.006 |
| NA228 | 0.064 | 0.574 | **0.013** | 0.313 | 0.026 | 0.001 | 0.007 |
| NA229 | 0.002 | 0.003 | **0.592** | 0.004 | 0.002 | 0.002 | 0.395 |
| NA230 | 0.037 | 0.308 | **0.004** | 0.121 | 0.002 | 0.004 | 0.524 |
| NA232 | 0.002 | 0 | **0.994** | 0.001 | 0 | 0.001 | 0.001 |
| NA235 | 0.006 | 0.001 | **0.678** | 0.005 | 0.001 | 0.015 | 0.293 |
| NA246 | 0.002 | 0.203 | **0.787** | 0.001 | 0.001 | 0.001 | 0.006 |
| NA26 | 0.017 | 0.003 | **0.619** | 0.02 | 0.003 | 0.007 | 0.332 |
| NA268 | 0.006 | 0.003 | **0.578** | 0.004 | 0.002 | 0.002 | 0.405 |
| NA283 | 0 | 0 | **0.998** | 0 | 0 | 0 | 0 |
| NA286 | 0 | 0 | **0.998** | 0 | 0 | 0 | 0 |
| NA289 | 0 | 0 | **0.998** | 0 | 0 | 0 | 0 |
| NA3 | 0.003 | 0.339 | **0.498** | 0.001 | 0.001 | 0.001 | 0.156 |
| NA30 | 0.028 | 0.003 | **0.001** | 0.653 | 0.308 | 0.007 | 0.001 |
| NA312 | 0.8 | 0.055 | **0.002** | 0.001 | 0.096 | 0.042 | 0.005 |
| NA32 | 0.004 | 0.004 | **0.533** | 0.003 | 0.002 | 0.001 | 0.452 |
| NA326 | 0 | 0 | **0.998** | 0 | 0 | 0 | 0 |
| NA327 | 0.007 | 0.138 | **0.724** | 0.069 | 0.041 | 0.002 | 0.019 |
| NA33 | 0.003 | 0.18 | **0.612** | 0.001 | 0.002 | 0.001 | 0.201 |
| NA337 | 0.061 | 0.002 | **0.856** | 0.024 | 0.001 | 0.015 | 0.041 |
| NA339 | 0.002 | 0.001 | **0.001** | 0.739 | 0.255 | 0.001 | 0.001 |
| NA34 | 0.004 | 0.022 | **0.598** | 0.01 | 0.007 | 0.001 | 0.357 |
| NA342 | 0 | 0 | **0.998** | 0 | 0 | 0 | 0 |
| NA387 | **0.993** | 0.001 | **0.002** | 0.001 | 0 | 0.003 | 0.001 |
| NA39 | 0.002 | **0.549** | **0.002** | **0.438** | 0.004 | 0.002 | 0.003 |
| NA399 | 0.002 | 0.001 | **0.001** | **0.74** | 0.255 | 0.001 | 0.001 |
| NA40 | 0.003 | 0 | **0.884** | 0.032 | 0 | 0.001 | 0.079 |
| NA423 | **0.984** | 0 | **0.004** | 0.004 | 0 | 0.005 | 0.002 |
| NA43 | 0.009 | 0.351 | **0.313** | 0.244 | 0.043 | 0.034 | 0.006 |
| NA432 | 0.003 | 0.106 | **0.803** | 0.003 | 0.043 | 0.003 | 0.039 |
| NA435 | 0 | 0 | **0.998** | 0 | 0 | 0 | 0 |
| NA45 | 0.002 | 0 | **0.886** | 0.034 | 0 | 0.001 | 0.076 |
| NA46 | 0.002 | 0 | **0.867** | 0.035 | 0 | 0.001 | 0.094 |
| NA47 | 0.131 | **0.851** | **0.012** | 0.001 | 0.001 | 0.003 | 0.002 |
| NA471 | 0.002 | **0.988** | **0.001** | 0.001 | 0.001 | 0.003 | 0.004 |
| NA475 | 0 | 0 | **0.998** | 0 | 0 | 0 | 0 |
| NA49 | 0.001 | 0.147 | **0.845** | 0 | 0.002 | 0.001 | 0.004 |
| NA504 | **0.992** | 0 | **0.001** | 0.002 | 0 | 0.003 | 0.001 |
| NA534 | **0.994** | 0 | **0.001** | 0.001 | 0 | 0.002 | 0.001 |
| NA58 | 0.008 | **0.535** | **0.035** | **0.362** | 0.008 | 0.01 | 0.042 |
| NA61 | 0.021 | 0.005 | **0.568** | 0.009 | 0.009 | 0.001 | 0.387 |
| NA66 | 0.006 | 0.003 | **0.578** | 0.004 | 0.002 | 0.002 | 0.405 |
| NA669 | 0.001 | 0.001 | **0.001** | **0.751** | **0.245** | 0.001 | 0.001 |
| NA670 | 0.002 | 0.113 | **0.694** | 0.003 | 0.073 | 0.002 | 0.113 |
| NA672 | 0 | 0 | **0.998** | 0 | 0 | 0 | 0 |
| NA674 | 0 | 0 | **0.998** | 0 | 0 | 0 | 0 |
| NA678 | 0 | 0 | **0.998** | 0 | 0 | 0 | 0 |
| NA680 | 0.001 | 0.001 | **0.001** | **0.751** | **0.245** | 0.001 | 0.002 |
| NA686 | 0.001 | 0.001 | **0.004** | **0.804** | 0.188 | 0.001 | 0.001 |
| NA687 | 0.007 | 0.01 | **0.009** | 0.145 | 0.148 | 0.001 | 0.68 |
| NA689 | 0.236 | 0.004 | **0.086** | 0.033 | 0.008 | 0.001 | 0.631 |
| NA691 | 0.001 | 0 | **0.998** | 0 | 0 | 0 | 0.001 |
| NA697 | 0 | 0 | **0.998** | 0 | 0 | 0 | 0 |
| NA70 | 0.01 | 0.144 | **0.528** | 0.005 | 0.001 | 0.001 | 0.31 |
| NA702 | 0.001 | 0 | **0.998** | 0 | 0 | 0 | 0 |
| NA705 | 0.002 | **0.502** | **0.016** | **0.394** | 0.052 | 0.019 | 0.014 |
| NA706 | 0.109 | **0.873** | **0.006** | 0.001 | 0.001 | 0.007 | 0.003 |
| NA71 | 0.003 | 0.001 | **0.7** | 0.01 | 0.001 | 0.004 | 0.282 |
| NA712 | 0.002 | 0.294 | **0.699** | 0.001 | 0.001 | 0.001 | 0.002 |
| NA715 | 0 | 0 | **0.998** | 0 | 0 | 0 | 0 |
| NA717 | 0.002 | 0.001 | **0.001** | **0.507** | **0.488** | 0.001 | 0.001 |
| NA718 | 0 | 0 | **0.998** | 0 | 0 | 0 | 0 |
| NA719 | 0.001 | 0.001 | **0.894** | 0.035 | 0 | 0.001 | 0.068 |
| NA720 | 0.002 | 0.232 | **0.749** | 0.002 | 0.01 | 0.002 | 0.003 |
| NA724 | 0.131 | **0.267** | **0.005** | **0.186** | 0.005 | 0.007 | **0.399** |
| NA728 | 0 | 0 | **0.998** | 0 | 0 | 0 | 0 |
| NA730 | 0 | 0 | **0.998** | 0 | 0 | 0 | 0 |
| NA732 | 0.037 | 0.001 | **0.958** | 0.001 | 0.001 | 0.003 | 0.001 |
| NA733 | 0 | 0 | **0.998** | 0 | 0 | 0 | 0 |
| NA739 | 0.06 | **0.882** | **0.05** | 0.001 | 0.001 | 0.002 | 0.004 |
| NA74 | 0.003 | 0.001 | **0.697** | 0.009 | 0.001 | 0.003 | 0.286 |
| NA750 | 0 | 0 | **0** | **0.998** | 0 | 0 | 0 |
| NA753 | 0.002 | 0.044 | **0.558** | 0.001 | 0.108 | 0.001 | 0.286 |
| NA756 | 0 | 0 | **0.998** | 0 | 0 | 0 | 0 |
| NA758 | 0.001 | 0.001 | **0.001** | 0.001 | 0 | 0.001 | **0.996** |
| NA759 | **0.953** | 0.001 | **0.002** | 0.026 | 0 | 0.012 | 0.005 |
| NA763 | 0.025 | 0.002 | **0.88** | 0.002 | 0.001 | 0.008 | 0.082 |
| NA764 | 0.002 | 0.001 | **0.001** | **0.417** | **0.576** | 0.001 | 0.001 |
| NA770 | 0 | 0 | **0.998** | 0 | 0 | 0 | 0 |
| NA773 | 0 | 0 | **0.998** | 0 | 0 | 0 | 0 |
| NA780 | 0.004 | **0.77** | **0.001** | 0.186 | 0.002 | 0.009 | 0.027 |
| NA79 | 0.028 | 0.181 | **0.5** | 0.049 | 0.047 | 0.037 | 0.158 |
| NA796 | 0.001 | 0.182 | **0.811** | 0 | 0.002 | 0.001 | 0.003 |
| NA8 | 0.006 | 0.196 | **0.509** | 0.003 | 0.001 | 0.003 | 0.282 |
| NA804 | 0.003 | 0.007 | **0.348** | **0.343** | 0.213 | 0.001 | 0.085 |
| NA807 | 0.002 | 0.161 | **0.662** | 0.004 | 0.156 | 0.002 | 0.013 |
| NA81 | 0.003 | 0.001 | **0.001** | **0.782** | 0.211 | 0.001 | 0.001 |
| NA831 | 0.003 | 0.203 | **0.619** | 0.001 | 0.001 | 0.001 | 0.171 |
| NA84 | 0.019 | 0.006 | **0.903** | 0.002 | 0.052 | 0.009 | 0.01 |
| NA851 | **0.989** | 0.001 | **0.002** | 0.001 | 0.001 | 0.004 | 0.002 |
| NA90 | 0.001 | 0 | **0.998** | 0 | 0 | 0 | 0.001 |
| PA1[PER] | **0.357** | **0.57** | 0.002 | 0.018 | 0.001 | 0.002 | 0.049 |
| PA107 | **0.857** | 0.036 | 0.002 | 0.007 | 0.059 | 0.014 | 0.025 |
| PA118 | **0.992** | 0 | 0.003 | 0.002 | 0 | 0.002 | 0.001 |
| PA12 | **0.986** | 0.001 | 0.001 | 0.007 | 0 | 0.002 | 0.003 |
| PA120 | **0.995** | 0 | 0.001 | 0.001 | 0 | 0.001 | 0.001 |
| PA121 | **0.995** | 0 | 0.001 | 0.002 | 0 | 0.001 | 0.001 |
| PA124 | **0.995** | 0 | 0.001 | 0.001 | 0 | 0.001 | 0.001 |
| PA125 | **0.871** | 0.008 | 0.001 | 0.003 | 0.044 | 0.013 | 0.06 |
| PA126 | **0.944** | 0.001 | 0.016 | 0.035 | 0.001 | 0.003 | 0.001 |
| PA128 | **0.996** | 0 | 0.001 | 0.001 | 0 | 0.001 | 0.001 |
| PA132 | **0.992** | 0 | 0.001 | 0.002 | 0 | 0.003 | 0.001 |
| PA135 | **0.992** | 0 | 0.002 | 0.003 | 0 | 0.002 | 0.001 |
| PA136 | **0.994** | 0 | 0.001 | 0.001 | 0.001 | 0.001 | 0.001 |
| PA137 | **0.995** | 0.001 | 0.001 | 0.001 | 0 | 0.001 | 0.001 |
| PA141 | **0.994** | 0 | 0.002 | 0.002 | 0 | 0.001 | 0.001 |
| PA150 | **0.084** | 0.515 | 0.013 | 0.314 | 0.06 | 0.003 | 0.011 |
| PA151 | **0.995** | 0 | 0.001 | 0.001 | 0 | 0.001 | 0.001 |
| PA156 | **0.958** | 0.001 | 0.003 | 0.035 | 0.001 | 0.001 | 0.002 |
| PA157 | **0.991** | 0.001 | 0.001 | 0.004 | 0.001 | 0.002 | 0.001 |
| PA16 | **0.92** | 0.051 | 0.002 | 0.002 | 0.001 | 0.022 | 0.001 |
| PA165 | **0.994** | 0 | 0.002 | 0.001 | 0 | 0.002 | 0.001 |
| PA169 | **0.953** | 0.001 | 0.002 | 0.026 | 0 | 0.012 | 0.005 |
| PA171 | **0.965** | 0.004 | 0.007 | 0.002 | 0.004 | 0.01 | 0.009 |
| PA173 | **0.984** | 0 | 0.002 | 0.011 | 0 | 0.001 | 0.001 |
| PA175 | **0.991** | 0 | 0.001 | 0.005 | 0 | 0.001 | 0.001 |
| PA184 | **0.987** | 0.001 | 0.002 | 0.001 | 0.001 | 0.008 | 0.001 |
| PA191 | **0.995** | 0 | 0.001 | 0.001 | 0 | 0.001 | 0.001 |
| PA195 | **0.995** | 0 | 0.001 | 0.001 | 0 | 0.001 | 0.001 |
| PA202 | **0.969** | 0.001 | 0.003 | 0.002 | 0.001 | 0.022 | 0.002 |
| PA211 | **0.995** | 0.001 | 0.001 | 0.001 | 0 | 0.001 | 0.001 |
| PA218 | **0.761** | 0.175 | 0.002 | 0.004 | 0.02 | 0.029 | 0.01 |
| PA27 | **0.99** | 0.001 | 0.001 | 0.001 | 0.001 | 0.005 | 0.001 |
| PA272 | **0.696** | 0.287 | 0.002 | 0.001 | 0.006 | 0.007 | 0.001 |
| PA279 | **0.991** | 0.001 | 0.001 | 0.002 | 0.001 | 0.003 | 0.002 |
| PA289 | **0.994** | 0 | 0.001 | 0.002 | 0 | 0.001 | 0.001 |
| PA291 | **0.993** | 0.001 | 0.001 | 0.001 | 0.001 | 0.003 | 0.001 |
| PA293 | **0.992** | 0 | 0.001 | 0.004 | 0 | 0.002 | 0.001 |
| PA296 | **0.996** | 0 | 0.001 | 0.001 | 0 | 0.001 | 0.001 |
| PA299 | **0.996** | 0 | 0.001 | 0.001 | 0 | 0.001 | 0.001 |
| PA3 | **0.973** | 0.001 | 0.003 | 0.02 | 0.001 | 0.002 | 0.001 |
| PA30 | **0.968** | 0 | 0.004 | 0.014 | 0 | 0.01 | 0.003 |
| PA300 | **0.936** | 0.011 | 0.002 | 0.001 | 0.01 | 0.037 | 0.002 |
| PA301 | **0.993** | 0 | 0.001 | 0.003 | 0 | 0.002 | 0.001 |
| PA303 | **0.994** | 0.001 | 0.001 | 0.001 | 0.001 | 0.002 | 0.001 |
| PA32 | **0.989** | 0.001 | 0.001 | 0.003 | 0.001 | 0.005 | 0.002 |
| PA34 | **0.986** | 0.001 | 0.002 | 0.008 | 0.001 | 0.001 | 0.003 |
| PA39 | **0.916** | 0 | 0.002 | 0.078 | 0.001 | 0.002 | 0.001 |
| PA4 | **0.99** | 0.002 | 0.001 | 0.001 | 0.001 | 0.004 | 0.001 |
| PA46 | **0.947** | 0 | 0.001 | 0.05 | 0 | 0.001 | 0.001 |
| PA67 | **0.687** | **0.301** | 0.001 | 0.001 | 0.007 | 0.002 | 0.002 |
| PA70 | **0.993** | 0 | 0.002 | 0.002 | 0 | 0.001 | 0.001 |
| PA84 | **0.995** | 0 | 0.001 | 0.002 | 0 | 0.001 | 0.001 |
| PA88 | **0.991** | 0.001 | 0.001 | 0.001 | 0.001 | 0.004 | 0.001 |
| PA95 | **0.994** | 0 | 0.001 | 0.003 | 0 | 0.001 | 0.001 |
| PLAYAALTA2 | 0.023 | 0.002 | 0.1 | **0.63** | **0.225** | 0.002 | 0.018 |
| Pound10_B | 0.002 | 0.002 | **0.982** | 0.003 | 0 | 0.001 | 0.009 |
| Pound10_C | 0.017 | 0.102 | **0.853** | 0.003 | 0.007 | 0.002 | 0.015 |
| Pound12_B | 0.001 | 0.001 | **0.001** | 0.772 | 0.223 | 0.001 | 0.001 |
| Pound16_B | 0.002 | 0.004 | **0.619** | 0.002 | 0.007 | 0.025 | 0.34 |
| Pound25_A | 0.002 | 0.004 | **0.62** | 0.002 | 0.007 | 0.025 | 0.339 |
| Pound26_C | 0 | 0 | **0.998** | 0 | 0 | 0 | 0 |
| Pound32_A | 0 | 0 | **0.998** | 0.001 | 0 | 0 | 0 |
| Pound7_A | 0.009 | 0.001 | **0.577** | 0.149 | 0.001 | 0.001 | 0.263 |
| RB29 | **0.298** | **0.402** | 0.022 | 0.003 | 0.026 | 0.005 | **0.244** |
| RIT22A2 | 0.006 | **0.428** | 0.007 | 0.165 | 0.019 | 0.003 | **0.371** |
| SC12 | 0.001 | 0.001 | 0.001 | **0.576** | **0.418** | 0.001 | 0.001 |
| SC15 | 0.002 | 0.421 | 0.002 | **0.544** | **0.008** | 0.004 | 0.018 |
| SC19 | 0.002 | 0.001 | 0.001 | **0.447** | **0.546** | 0.002 | 0.001 |
| SC20 | 0.002 | 0.001 | 0.001 | **0.503** | **0.492** | 0.001 | 0.001 |
| SC3 | 0.002 | 0.001 | 0.001 | **0.502** | **0.492** | 0.001 | 0.001 |
| SC4 | 0.002 | 0.001 | 0.001 | **0.503** | **0.49** | 0.001 | 0.001 |
| SC6 | 0.001 | 0 | 0.001 | 0 | 0 | **0.997** | 0 |
| SCA10 | 0.002 | **0.001** | 0.001 | **0.783** | 0.212 | 0.001 | 0.001 |
| SCA11 | 0.095 | **0.886** | 0.01 | 0.001 | 0.001 | 0.002 | 0.006 |
| SCA12 | 0.096 | **0.885** | 0.009 | 0.001 | 0.001 | 0.002 | 0.006 |
| SCA20 | 0.024 | **0.029** | 0.247 | 0.015 | 0.004 | 0.209 | **0.472** |
| SCA3 | 0.001 | **0.001** | 0.001 | 0.001 | 0 | 0.001 | **0.996** |
| SCA5 | 0.127 | **0.847** | 0.006 | 0.001 | 0.001 | 0.01 | 0.008 |
| SCA6 | 0.057 | **0.926** | 0.003 | 0.001 | 0.001 | 0.002 | 0.01 |
| SCA9 | 0.011 | **0.01** | **0.515** | 0.02 | 0.004 | 0.002 | 0.438 |
| SIC864 | 0.001 | 0 | 0 | 0.996 | 0 | 0.001 | 0 |
| SNK10 | 0.002 | 0.001 | 0.001 | **0.671** | **0.324** | 0.001 | 0.001 |
| SNK13 | 0.002 | 0.001 | 0.001 | **0.842** | **0.153** | 0.001 | 0.001 |
| SNK16 | 0.002 | 0.001 | 0.001 | **0.729** | **0.264** | 0.001 | 0.001 |
| SNK413 | 0.001 | 0.001 | 0.001 | **0.746** | **0.249** | 0.001 | 0.001 |
| SPA10 | 0.002 | 0.007 | 0.472 | 0.001 | 0.002 | 0.001 | **0.515** |
| SPA18 | 0.002 | 0.002 | 0.52 | 0.001 | 0.001 | 0.001 | **0.473** |
| SPA4 | 0.001 | 0.163 | 0.829 | 0.001 | 0.002 | 0.001 | **0.003** |
| SPA7 | 0.007 | 0.032 | 0.001 | 0.235 | 0.148 | 0.001 | **0.575** |
| SPA9 | 0.003 | 0.005 | 0.461 | 0.001 | 0.001 | 0.002 | **0.527** |
| SPEC138_13 | 0.488 | 0.294 | 0.002 | **0.197** | **0.002** | 0.003 | 0.013 |
| SPEC160_9 | 0.002 | 0.001 | 0.002 | **0.437** | **0.556** | 0.001 | 0.001 |
| SPEC18_6 | 0.004 | 0.133 | 0.129 | **0.212** | **0.006** | 0.134 | 0.382 |
| SPEC185_4 | 0.001 | 0.001 | 0.001 | **0.523** | **0.473** | 0.001 | 0.001 |
| SPEC194_103 | 0.002 | 0.001 | 0.001 | **0.394** | **0.599** | 0.002 | 0.001 |
| SPEC194_109 | 0.001 | 0.001 | 0.002 | **0.434** | **0.56** | 0.001 | 0.001 |
| SPEC194_48 | 0.002 | 0.001 | 0.001 | **0.665** | **0.33** | 0.001 | 0.001 |
| SPEC194_62 | 0.002 | 0.001 | 0.001 | **0.577** | **0.417** | 0.001 | 0.001 |
| SPEC194_75 | 0.011 | 0.169 | 0.56 | **0.003** | **0.006** | 0.004 | 0.247 |
| SPEC41_11 | 0.001 | 0 | 0.223 | **0.773** | **0** | 0.001 | 0.001 |
| SPEC41_6_18 | 0.001 | 0 | 0.224 | **0.772** | **0** | 0.001 | 0.001 |
| TSH1077 | **0.137** | 0.049 | 0.008 | 0.182 | 0.142 | 0.005 | **0.477** |
| TSH1188 | **0.128** | 0.202 | 0.034 | 0.152 | 0.022 | 0.003 | **0.458** |
| TSHS76 | 0.072 | **0.502** | 0.03 | **0.348** | 0.013 | 0.002 | 0.033 |
| UF11 | 0.002 | 0.001 | 0.001 | **0.507** | **0.488** | 0.001 | 0.001 |
| UF168 | 0.002 | 0.001 | 0.001 | **0.507** | **0.488** | 0.001 | 0.001 |
| UF667 | 0.001 | 0.001 | 0.001 | **0.507** | **0.488** | 0.001 | 0.001 |
| UF676 | 0.001 | 0.001 | 0.001 | **0.507** | **0.488** | 0.001 | 0.001 |
