# Supplementary material for: Genome-wide association studies and genomic selection assays made in a large sample of cacao (Theobroma cacao L.) germplasm reveal significant marker-trait associations and good predictive value for improving yield potential
Source: PLoS One. 2022 Oct 6;17(10):e0260907. doi: 10.1371/journal.pone.0260907 (PMC9536643; doi:10.1371/journal.pone.0260907)
Supplement: S7 Table — http://dx.doi.org/10.13140/RG.2.2.20793.44647. (DOCX) [file pone.0260907.s007.docx]

**S7 Table. Summary of Linkage disequilibrium decay results.**

| **Chromosome** | ***r^2^*max** | **50% *r^2^* max** | **Distance corresponding to 50% *r^2^* max (Mb)** | **Distance corresponding to 50% r^2^ max (cM)** | **Remark** |
| --- | --- | --- | --- | --- | --- |
| 1 | 0.2104 | 0.1052 | 14.289 | 24.69 |  |
| 3 | 0.20398 | 0.10199 | n/a | n/a | correlation between alleles was not detected at 50% of *r^2^* max or below |
| 4 | 0.18411 | 0.0921 | 3.178 | 5.49 |  |
| 5 | 0.15367 | 0.07684 | 5.231 | 9.05 |  |
| 7 | 0.2146 | 0.10732 | 1.733 | 2.99 |  |
| 9 | 0.21068 | 0.10534 | 1.723 | 2.98 |  |

**Legend**

Average LD decay to 50% over chromosomes 1, 4, 5, 7 and 9 is 5.23 Mbp (9.1 cM)

1cM = 573 Kbp (0.573 Mbp)

1.745 cM = 1 Mbp
