# Supplementary material for: Genome-wide association studies and genomic selection assays made in a large sample of cacao (Theobroma cacao L.) germplasm reveal significant marker-trait associations and good predictive value for improving yield potential
Source: PLoS One. 2022 Oct 6;17(10):e0260907. doi: 10.1371/journal.pone.0260907 (PMC9536643; doi:10.1371/journal.pone.0260907)
Supplement: S9 Table — http://dx.doi.org/10.13140/RG.2.2.12404.83842. (DOCX) [file pone.0260907.s009.docx]

**S6 Table. Predictive values (GEBV) of phenotypic traits associated with SNPS.**

| **Phenotypic Trait** | **GEBV** |
| --- | --- |
| Ovule number | 0.6325 |
| Seed/bean number | 0.6110 |
| Seed/bean mass (g) | 0.6014 |
| Seed length (cm) | 0.6199 |
| Pod width (cm) | 0.5849 |
| Total wet seed mass (g) | 0.5656 |
| Seed width (cm) | 0.5435 |
| Seed length to width ratio (log transformed) | 0.5503 |
| Filament anthocyanin intensity | 0.5486 |
| Pod Index (log transformed) | 0.5394 |
| Pod basal constriction | 0.5116 |
| Pod index (not transformed) | 0.4975 |
| Pod wall hardness | 0.5134 |
| Sepal width (mm) | 0.4919 |
| Sepal length (mm) | 0.4366 |
| Ligule width (mm) | 0.4720 |

GEBV indicates the correlation between the predicted value per trait based on genotype and the observed value based on the measured phenotype
