# Supplementary figures and images for: Genome-wide association studies and genomic selection assays made in a large sample of cacao (Theobroma cacao L.) germplasm reveal significant marker-trait associations and good predictive value for improving yield potential
Source: PLoS One. 2022 Oct 6;17(10):e0260907. doi: 10.1371/journal.pone.0260907 (PMC9536643; doi:10.1371/journal.pone.0260907)

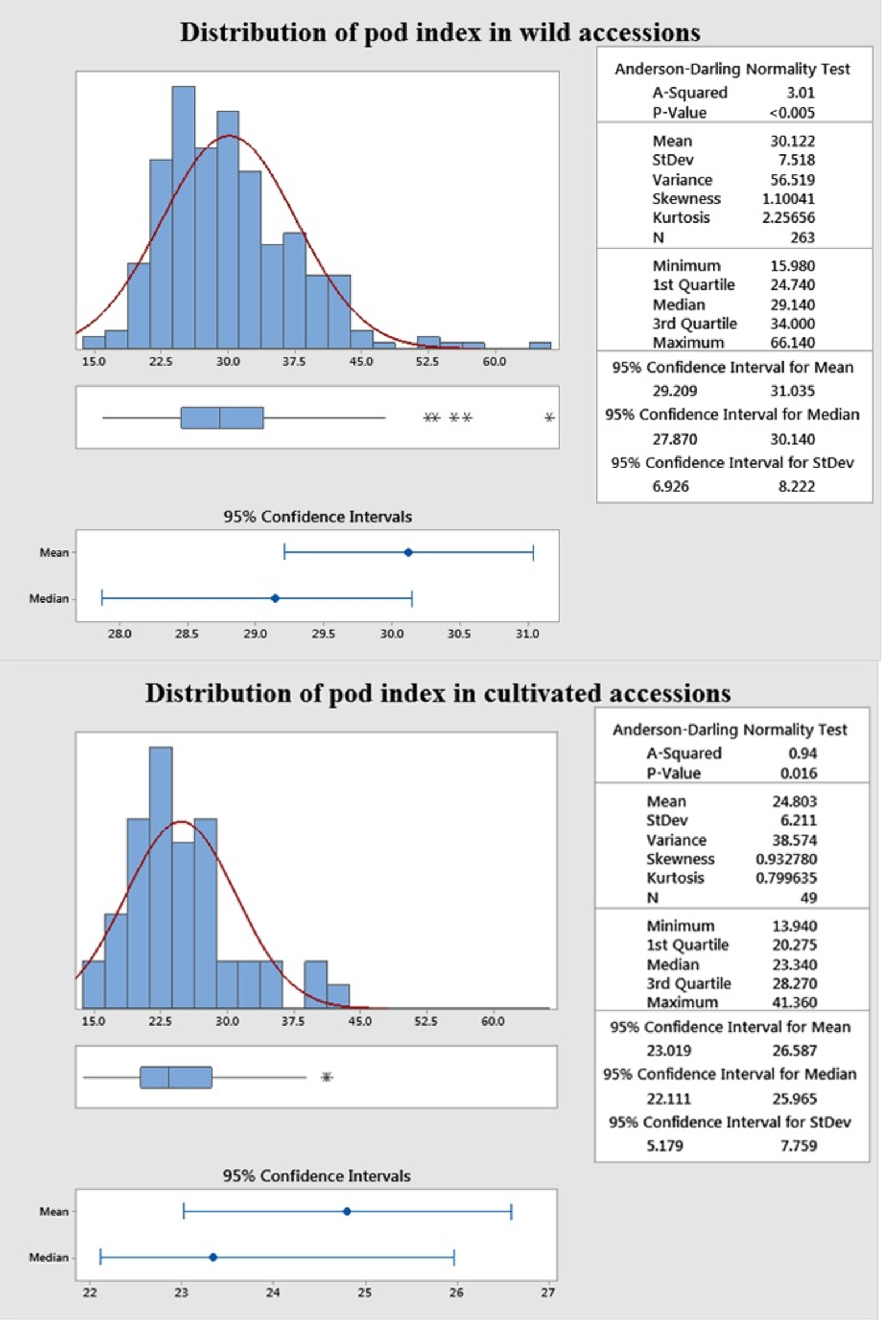

Supplement: S1 Fig — http://dx.doi.org/10.13140/RG.2.2.24148.88966. (TIF) [file pone.0260907.s011.tif]
